# Evaluation of nutritional value of *Asystasia mysorensis* and *Sesamum angustifolia* and their potential contribution to human health

**DOI:** 10.1002/fsn3.1064

**Published:** 2019-05-15

**Authors:** Ernest G. Maina, Edwin S. Madivoli, Josephine A. Ouma, Joel K. Ogilo, Jackson M. Kenya

**Affiliations:** ^1^ Chemistry Department Jomo Kenyatta University of Agriculture and Technology Nairobi Kenya

**Keywords:** ascorbic acid, GC‐MS profile, micronutrient, secondary metabolites, β‐carotene

## Abstract

Wild indigenous vegetables make considerable contributions to food baskets among subsistence farmers in sub‐Saharan Africa. The aim of this study was to evaluate the proximate analysis, mineral composition, vitamin C content, β‐carotene content, and GC‐MS profile of crude methanolic extracts of *Asystasia mysorensis* and *Sesamum angustifolia*. Crude extracts obtained through sequential extraction using ethyl acetate and methanol were screened for the presence of secondary metabolites. Functional groups present were determined with a Shimadzu FT‐IR spectrophotometer, while β‐carotene content and ascorbic acid content were evaluated using a Shimadzu HPLC and Shimadzu UV‐VIS spectrophotometer, respectively. Secondary metabolites present in the extracts were determined qualitatively using a Shimadzu GC‐MS system equipped with a NIST spectral database. From the results obtained, the two plants could supply the recommended daily requirement for micronutrient and vitamin C content needed for a healthy diet. The total phenolic and flavonoid contents in *S. amgustifolia* were higher as compared to *A. myorensis;* hence, their consumption is highly beneficial as some compounds identified in the GC‐MS profile have been reported to have medicinal properties. The findings on the mineral and chemical composition, GC‐MS profile of *A. mysorensis* and *S. angustifolia* indicate that their consumption may provide the recommended nutritional requirements needed for a healthy diet.

## 1 INTRODUCTION

Today, 842 million people around the world are undernourished despite the fact that there is enough food globally. It is estimated that 26 percent of children around the world are stunted, and almost 30 percent of the world's population suffers from micronutrient deficiencies due to uptake of diets that are devoid of necessary micronutrients (Durst & Bayasgalanbat, 2014; Tribaldos, Jacobi, & Rist, 2018). Currently, there are about 1.4 billion overweight adults worldwide, of which 500 million are obese and at risk of contracting lifestyle diseases such as diabetes, high blood pressure, cardiovascular disease, and various cancers (Durst & Bayasgalanbat, 2014). According to an economic survey carried out in 2007, fifty‐one percent of Kenyans lack access to adequate food and poverty, which is estimated to be forty‐six percent nationally, and it has been associated with food security (Joshi, 2012; Muthoni & Nyamongo, 2010). Moreover, Kenya continues to face a challenge in food availability, due to high cost of farm inputs, inadequate rains, postelection violence, and spread of livestock and plant diseases such as rift valley fever and armyworm infestations (Muthoni & Nyamongo, 2010; Oyas et al., 2018). Many communities in developing countries, such as Kenya, consume wild edible plants that have a much higher nutrient content than globally known varieties or species but they are often underutilized (Durst & Bayasgalanbat, 2014). These indigenous green leafy vegetables are climate tolerant; hence, they are less damaging to the environment, address cultural needs, and assist in the preservation of the culture of local communities and they have been reported to be good sources of macro‐ and micronutrients (Maseko et al., 2017; Uusiku, Oelofse, Duodu, Bester, & Faber, 2010). However, there is still a high prevalence of malnutrition, especially micronutrient deficiencies among low‐ or marginal‐income bracket of the population (Maseko et al., 2017). The use of indigenous vegetables has been proposed as part of the solution to the problems of micronutrient malnutrition among these populations (Durst & Bayasgalanbat, 2014). These vegetables have been reported to assist in managing hunger, influence the intake of cereal staples, and play a key role in household food security among the poorer rural groups as they are rich in micronutrients, vitamins, and secondary metabolites (Maseko et al., 2017; Muthoni & Nyamongo, 2010). They contain essential vitamins such as A, B, and C and essential mineral elements such as calcium and iron as well as protein and calories that can eliminate dietary deficiencies. Because of their medicinal value, people suffering from medical conditions such as high blood pressure, HIV/AIDS, cancer, and hypertension are often encouraged to consume them (Muhanji, Roothaert, Webo, & Mwangi, 2011). Moreover, dietary changes from traditional low‐fat, plant‐based proteins toward high‐fat, animal proteins have received considerable attention due to their contributing factor to the increased occurrence of chronic lifestyle diseases (Hung et al., 2004). These vegetables compare well with Swiss chard, cabbage, and spinach in terms of micronutrient levels, while dark green leafy vegetables (DGLV) have been reported to be a rich source of folate and linoleic acids (Van der Walt, Ibrahim, Bezuidenhout, & Loots, 2008). Nutritional data on wild varieties of traditional African green leafy vegetables are fragmentary and almost nonexistent for wild‐growing *Asystasia mysorensis* and *Sesamum angustifolia*. The present study reports on the nutritional value, phytochemical screening, GC‐MS profiles, vitamin C, and β‐carotene content of two wild‐growing indigenous vegetable species *A. mysorensis* and *S. angustifolia*.

## 2 METHODOLOGY

### 2.1 Extraction of plant material

Cold sequential extraction of *A. mysorensis* and *S. angustifolia* was carried out using ethyl acetate and methanol as the extracting solvents. Hundred grams of the plant powders was macerated in 1,000 ml ethyl acetate followed by methanol at room temperature. The extracts were filtered using Whatman No. 1 filter paper and concentrated using a Rota evaporator (BUCHI R 200; Labortechnik) set at 40°C. The crude extracts were then left in a fume chamber to dry, after which they were stored at 4°C until further analysis (Madivoli et al., 2018).

### 2.2 Phytochemical screening of plant extracts

Standard established procedures for identifying metabolites were used to carry phytochemicals screening on the ethyl acetate and methanol extracts as described by Harborne (1998). An aliquot of every plant extract was analyzed for the presence of saponins, alkaloids, terpenoids, phenols, and tannins (Ezeonu & Ejikeme, 2016; Madivoli et al., 2018).

### 2.3 Proximate analysis of plant samples

The powdered plant samples were analyzed for moisture content, protein, fat, and ash content by methods adopted from literature, while carbohydrate content was determined by difference (100−[% moisture + % protein + % fat + % ash + % fiber]) (Olaniyi, Lawal, & Olaniyi, 2018; Rangani, Kumari, Patel, Brahmbhatt, & Parida, 2019; Thangaraj, 2016).

### 2.4 Characterization of crude extracts

The crude extracts were characterized using a Fourier transform infrared spectrophotometer, Shimadzu FTS‐8000 (Shimadzu Corporation). The KBr pellets of the extracts were prepared by mixing 10 mg of finely grounded samples, with 250 mg KBr (FT‐IR grade). The spectral resolution was set at 4 cm^–1^ and the scanning range from 400 to 4,000 cm^–1 ^(Madivoli et al., 2018).

### 2.5 Total phenolic content

The total phenolic content of the crude extracts was evaluated by the Folin–Ciocalteu method with some modifications (Thangaraj, 2016). 0.1 g of plant material was extracted with 4.9 ml 80% methanol and filtered through a Whatman No. 1 filter paper to make the stock sample. Fifty microliter of the stock sample was made to 1 ml with distilled water and then 0.5 ml of 1 N Folin–Ciocalteu reagent and incubated for 5 min. 2.5 ml of 5% Na_2_CO_3 _was then added and the total volume made to 4 ml using distilled water. The resultant solution was incubated at room temperature for 40 min. The same quantity of the reagents was used to prepare the calibration standards. Both distilled water and garlic acid (0–75 µg/ml) were used to produce standard calibration curve, and the total phenolic content was expressed in mg of garlic acid equivalents (GAE)/g of dry weight extract (DW). Absorbance was measured at 769 nm using a Shimadzu 1800 UV‐VIS spectrophotometer (Baba & Malik, 2015; Madivoli et al., 2018; Mburu et al., 2016; Thangaraj, 2016).

### 2.6 Total flavonoid content

Aluminum chloride method was used to determine the total flavonoid content as described in literature (Baba & Malik, 2015; Mburu et al., 2016; Thangaraj, 2016). 0.1 g of plant material was extracted with 4.9 ml 80% methanol and filtered with Whatman No. 1 filter paper to make the stock sample. One hundred microliter of the stock sample was made to 1 ml with distilled water. Then to this solution, 150 µl of 5% sodium nitrite (NaNO_2_) and the contents were vortexed and incubated for 5 min at room temperature. One hundred fifty microliter of 10% aluminum trichloride (AlCl_3_) was then added, and the solution was vortexed and then incubated for 6 min at room temperature. 2.0 ml of sodium hydroxide was then added, and the solution was made to 5.0 ml and incubated at room temperature for 15 min. The same quantity of the reagents was used to prepare the calibration standards. Both distilled water and rutin (0–75 µg/ml) were used to generate the standard calibration curve, and the total flavonoid content was expressed as mg of rutin equivalents (RE)/g of dry weight (DW) extract. Absorption readings were carried out at 511 nm using Shimadzu 1800 UV‐VIS spectrophotometer (Madivoli et al., 2018; Mburu et al., 2016; Thangaraj, 2016).

### 2.7 Estimation of macro‐ and micronutrient content

The concentration of micronutrient, macronutrients, and toxic elements in *A. mysorensis* and *S. angustifolia* was evaluated using Agilent 7900 ICP‐MS (Agilent) after acid digestion. One gram of dried material was digested with 12 ml of HCl: HNO_3_ (1:3) to remove all organic matter from the plant samples. After digestion, the residue was washed with distilled water and filtered into a 50‐ml volumetric flask and topped to the mark to await further analysis using an FAAS (Kumari, Parida, Rangani, & Panda, 2017; Thangaraj, 2016; Uddin et al., 2016; Yami, Chandravanshi, Wondimu, & Abuye, 2016).

### 2.8 Estimation of β‐carotene content

Approximately 2 g of plant samples was extracted using 50 ml of acetone twice followed by concentrating to 1 ml using a Bibby Sterling RE 100B UK rotary evaporator. The extracts were then eluted through a chromatographic column which was packed with silica gel to elute the beta‐carotene as a yellow pigment which was collected in a 25‐ml flask. Five solutions of standard beta‐carotene whose concentration range was 0.5–2.5 µg/ml were then prepared from a stock solution containing 2.5 µg/ml β‐carotene. The concentration of beta‐carotene in the plant samples was then estimated using a Shimadzu 1800 UV‐VIS spectrophotometer (Shimadzu Corporation) set at 440 nm (Fungo et al., 2017; Kumari et al., 2017).

### 2.9 Estimation of ascorbic acid content

The vitamin C content of the plant samples was determined using high‐performance liquid chromatography (HPLC) (LC 6 A, Shimadzu) using a C18 (ODS) column (50 mm i.d × 30 cm) equipped with a UV detector set at 266 nm. The extract was obtained after 2 g of each sample was extracted with 0.8% metaphosphoric acid, followed by centrifugation at 10397.4 *g*, filtering through 0.45‐µM filter and diluted with 10 ml of 0.8% metaphosphoric acid. A calibration curve was obtained by preparing a series of standard solutions comprising of ascorbic acid at different concentrations which were used to estimate the vitamin C content of the plant samples (Rangani et al., 2019; Vikram, Ramesh, & Prapulla, 2005).

### 2.10 GC‐MS analysis

GC‐MS analysis of crude hexane and methanol extracts was evaluated using a Shimadzu GC‐MS QP2010SE. A Shimadzu GC‐MS QP2010SE (Shimadzu Corporation) operating in EI mode at 70 eV equipped with a NIST spectral database was used for the qualitative identification of compounds present in the extracts. A BPX5 capillary column 30 m × 0.25 mm (id) and Helium gas with a flow rate of 1.2 ml/min were used as the carrier gas, while the oven temperature and the mass range were set at 60°C and 40–400 m/z, respectively (Madivoli et al., 2018).

## 3 RESULTS AND DISCUSSIONS

### 3.1 Qualitative phytochemical screening

The phytochemicals tested include saponins, alkaloids, tannins, glycosides, and flavonoids and the results are presented in Table 1.

**Table 1:** Phytochemical screening of crude extracts of *Asystasia mysorensis* and *Sesamum angustifolia*

Plant species	Saponins	Alkaloids	Terpenoids	Steroids	Cardiac glycosides	Tannins
*A. mysorensis*	++	+	++	+	+	+
*S. angustifolia*	−	+	+++	++	+	+

Note

“−”, absent; “+”, present in small amount; “++”, present in large amount; “+++”, present in very large amounts.

Important phytochemicals such as saponins, steroids, flavonoids, phenolic compounds, and tannins were found to be present in *A. mysorensis* and *S. angustifolia* crude extracts. Thus, phytochemical screening serves as the initial step in predicting the types of potentially active compounds present plant samples (Cheruiyot et al., 2015). It has been reported that alkaloids tend to intercalate DNA thereby inhibiting cell division, while phenolics and polyphenols such as flavonoids, quinones, tannins, and coumarins all exert remarkable antifungal activity (Gupta & Birdi, 2017).

### 3.2 Proximate analysis

The analysis of proximate composition of *A. mysorensis* and *S. angustifolia* is depicted in Table 2.

**Table 2:** Proximate composition of *Asystasia mysorensis* and *Sesamum angustifolia* plants on a dry basis

	*A. mysorensis*	*S. angustifolia*
Moisture content (%)	13.56 ± 0.03	14.39 ± 0.32
Ash content (%)	21.06 ± 0.01	22.37 ± 0.71
Crude fat (%)	2.42 ± 0.04	2.20 ± 0.44
Crude protein (%)	25.29 ± 0.35	26.26 ± 0.26
Total carbohydrates (%)	35.79 ± 0.35	34.82 ± 0.26

From the results obtained in this study, *S. angustifolia* had a higher moisture content, ash content, total carbohydrate, and total proteins as compared to *A. mysorensis* which had a higher crude fat content. The difference between the contents of the two plants may be as a result of different growth conditions, genetic variation, stage of maturity, or due to differences in postharvest handling (Fungo et al., 2017). The results obtained indicate that dried *A. mysorensis* and *S. angustifolia* plants are a good source of dietary fibers, minerals (ash), protein, and energy, but not a good source of edible fat given the fact that drying lowers the proximate composition of vegetables as reported elsewhere (Hassan et al., 2007).

### 3.3 Total phenolic and total flavonoid content

**Figure 1:**
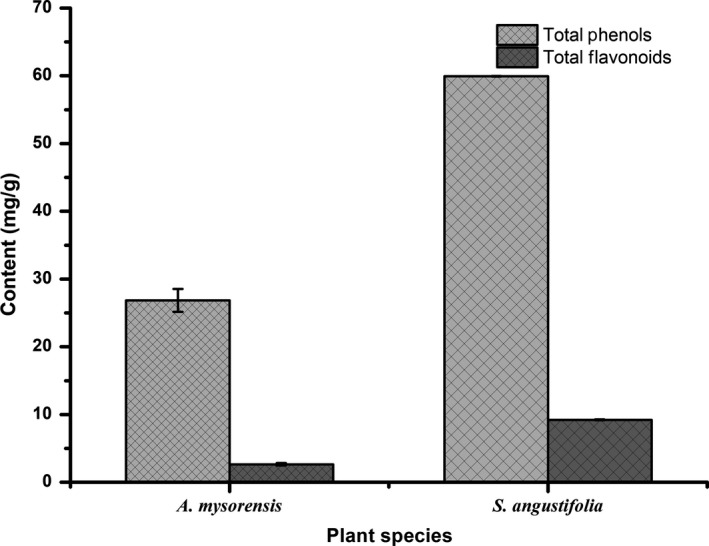
Quantitative total phenolic content (mg GE/g DW) and total flavonoid content (mg RE/g DW) of *Asystasia mysorensis* and *Sesamum angustifolia* plant extracts

The total phenolic content and total flavonoid content of *A. mysorensis* and *S. angustifolia* plant extracts are depicted in Figure 1 as garlic acid equivalent per dry weight (mg GE/g DW) and rutin equivalent per dry weight (mg RE/g DW), respectively.

Phenols are secondary metabolites that are prepared by plants as a defense mechanism to protect themselves against parasitic organisms (Tamokou, Mbaveng, & Kuete, 2017). From the results obtained in this study, *S. angustifolia* had the highest total phenolic content of 59.93 ± 0.05 mg GE/g DW, while *A. mysorensis* had a total phenolic content of 26.5 ± 1.67 mg GE/g DW, respectively. Apart from supplying the required nutrients, green leafy vegetables may provide a host of other components such as non‐nutrient phytochemicals that can have a positive impact on human health. Dietary polyphenols have been associated with lowered risks of chronic lifestyle diseases such as cancer and cardiovascular diseases because they have the ability to neutralize free radicals through electron donation or hydrogen atom (Quinones, Miguel, & Aleixandre, 2013; Tsao, 2010; Zhou et al., 2016). The regular intake of dietary green leafy vegetables that have a high concentration of phenolic compounds has been reported to reduce the risk of lifestyle‐related diseases (Moyo et al., 2018). Generally, plant extracts that contain a high concentration of polyphenols have been reported to exhibit high antioxidant activity (Uusiku et al., 2010). Flavonoids such as myricetin, quercetin, kaempferol, isorhamnetin, and luteolin have been reported to be present in leafy vegetables (Uusiku et al., 2010). Quantitative total flavonoid content of *A. mysorensis* and *S. angustifolia* plant extracts is depicted in Figure 1. From the results obtained in this study, *S. angustifolia* had a higher total flavonoid content of 9.22 ± 0.06 mg RE/g DW as compared to *A. mysorensis* extracts which had total flavonoid content of 2.65 ± 0.17 mg RE/g DW, respectively. Non‐nutritional components of wild green vegetables such as phenolic compounds have been reported to possess powerful radical scavenging properties against reactive oxygen species (ROS) (Podsedek, 2007).

### 3.4 FT‐IR characterization

**Figure 2:**
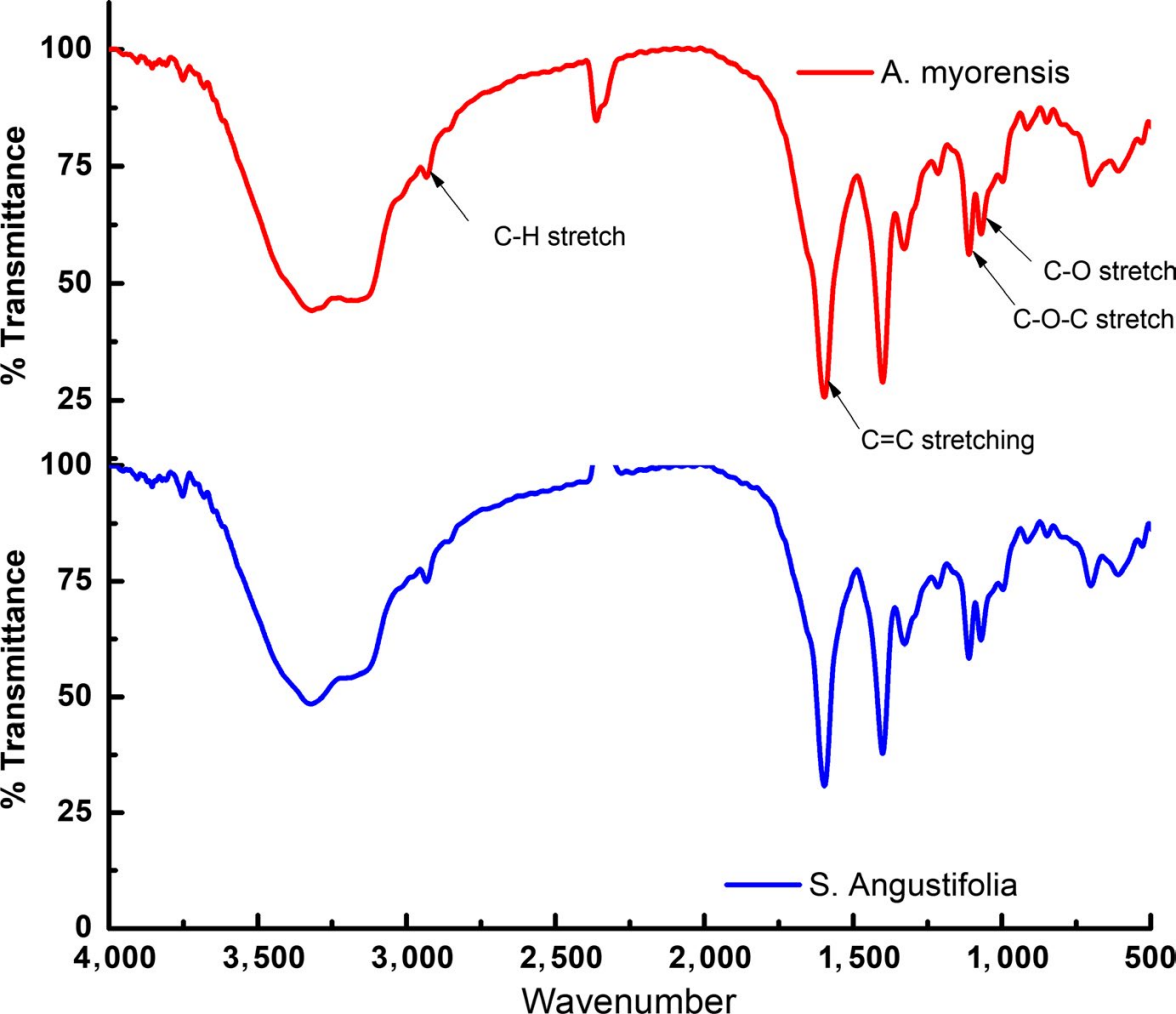
FT‐IR Spectra of *Asystasia mysorensis* and *Sesamum angustifolia*

Crude extracts of both *A. mysorensis* and *S. angustifolia* were assayed to determine the functional groups present and the results are depicted in Figure 2.

From the spectrum obtained, the crude extracts revealed presence of hydroxyl groups that are characteristic of alcohols at around 3,300 cm^−1^, CH_2_ functional group at 2,900 cm^−1^, and C‐O‐C functional groups at 1,100 cm^−1^. The presence of these functional groups is an indication of presence secondary metabolites such as glycosides, tannins, flavonoids, phenols, and saponins (Sasidharan, Chen, Saravanan, Sundram, & Latha, 2011).

### 3.5 Micro‐ and macronutrient content

The micronutrient, macronutrients, and toxic elements present in the samples were determined using a Agilent ICP‐MS, and the results are depicted in Table 3.

**Table 3:** Mean concentrations (mg/g) and standard deviations of macro‐ and micronutrient of *Asystasia mysorensis* (mg/g) and *Sesamum angustifolia* (mg/g)

Micronutrient	*A. mysorensis* (mg/kg)	*S. angustifolia* (mg/kg)	RDA children mg/day	RDA Male mg/day	RDA females mg/day
Aluminum	323.11 ± 0.95	568.61 ± 1.35	Non	Non	Non
Calcium	545.371 ± 0.95	257.93 ± 1.90	700	1,000	1,000
Cesium	10.01 ± 0.05	322.5 ± 3.55	Non	Non	Non
Chromium^a^	11.98 ± 1.5	21.59 ± 0.6	15	35^a^	25^a^
Cobalt	0.14 ± 0.01	0.18 ± 0.01	0.1^a^	0.1^a^	0.1^a^
Copper	0.91 ± 0.01	0.99 ± 0.04	0.7	0.9	0.9
Iron	957.91 ± 0.4	875.77 ± 0.9	11.6	8	18
Magnesium	1,492.22 ± 0.55	721.41 ± 1.35	130	420	320
Manganese	204.29 ± 0.9	400.41 ± 1.3	1.5	2.3	1.8
Molybdenum^a^	0.43 ± 0.001	0.0014 ± 0.0	22	45	45
Nickel	11.157 ± 0.65	14.93 ± 1.6	Non	Non	Non
Potassium	17,466.90 ± 1.05	8,141.06 ± 1.2	3.8	4.7	4.7
Selenium	BDL	0.25 ± 0.1320	30	55	55
Sodium	95.62 ± 1.3	78.92 ± 0.95	1.2	1.5	1.5
Zinc	1.70 ± 0.01	1.22 ± 0.02	4.5	12	8

a µg/kg.

The micro‐ and macronutrient contents of green leafy vegetables vary widely and are influenced by several factors such as stage of maturity and postharvest handling (Moyo et al., 2018). In the present study, the vegetables were obtained from the wild in a fresh state at the sampling point to determine the micronutrient concentration available to consumers. The mineral content in the two vegetables was either in the range or higher than mineral content reported in other indigenous vegetables. In comparison with the estimated FAO/WHO recommended daily intake and the estimated daily portion intake for adults and children, the two indigenous vegetables can have a substantial contribution to the requirements (FAO/WHO, 2004; Fungo et al., 2017). Analysis of the micro‐ and macronutrient content of the two indigenous vegetables against RDA revealed that a portion of the vegetables can supply more than the recommended minimum daily allowance of micro‐ and macronutrients such as potassium, sodium, zinc, iron, chromium, manganese, cobalt, copper, and other essential elements. Of all the minerals studied, the two vegetable can contribute immensely to the RDA requirements for iron, zinc, copper, chromium, and manganese given iron and zinc deficiencies are a serious problem in Kenya and sub‐Saharan Africa region (Pinstrup‐Andersen, 2009). Even though it is needed in very small quantities in the diet, cobalt is another essential mineral that forms part of vitamin B12, cobalamin, which supports production of red blood cell and the formation of myelin nerve coverings. No specific recommended daily allowance has been suggested for cobalt since dietary needs are very low, and they are fulfilled by vitamin B12. Considerably, high iron content has also been reported for some wild, traditional leafy green vegetables (Schonfeldt & Pretorius, 2011). Iron plays a crucial role as an oxygen carrier from lungs to body tissues, as a transport medium for electrons within cells and an integral part of important enzyme systems such as cytochromes (Moyo et al., 2018). The mineral content found in African leafy vegetables has been reported to exceed the levels found in exotic vegetables such as cabbage (Maseko et al., 2017; Rahmdel et al., 2018). Analysis of toxic elements such as lead and mercury also revealed that the two plants had an appreciable amount of these elements which can be as a result of absorption from the environment. The metal concentrations of vegetables in this study were significantly different and could be attributed to the differences in their morphology and physiology for heavy metal uptake, exclusion, accumulation, and retention (Rahmdel et al., 2018).

### 3.6 Vitamin C content

The vitamin C contents in the *A. mysorensis* (mg/g) and *S. angustifolia* (mg/g) were determined using Shimadzu HPLC. *S. angustifolia* recorded the highest vitamin C content of 92.42 mg/g, while *A. mysorensis* had the lowest value of 42.04 mg/g. The two vegetables recorded vitamin C contents that were higher than the recommended daily intake of 40–70 mg/100 g if consumed in large quantity. Analysis of the vitamin C content of the two indigenous vegetables against RDA revealed that a portion of the vegetables can supply more than the recommended minimum daily requirements of 75 mg/day for males and 60 mg/day for females between the ages of 19 and 70 years, respectively. The two vegetables can be a substituent for conventional vegetables such as spinach which are largely consumed to meet the RDA. Moreover, vitamin C is a potent antioxidant which plays an important role as an electron donor for enzymes involved in collagen hydroxylation, tyrosine metabolism, and carnitine biosynthesis (Prockop & Kivirikko, 1995). The high vitamin C content of *A. mysorensis* and *S. angustifolia* makes the two vegetables compatible to use with starchy staples because they contain ascorbic acid, which enhances iron absorption (Maseko et al., 2017). Ascorbic acid promotes absorption of soluble nonheme iron through chelation or by maintaining the iron in the reduced form. In addition, it also significantly counteracts the inhibition of iron absorption by phytates in the diet. Besides its ability to scavenge free radicals, ascorbic acid also plays a part in the regeneration of other antioxidants species such as tocopheroxyl and the carotene radical cation from their radical species (Uusiku et al., 2010).

### 3.7 Beta‐carotene content

Beta‐carotene content was determined calorimetrically after extraction with acetone and separation by column chromatography. Beta‐carotene content was evaluated by taking absorbance readings at 470 nm against a blank sample. The experimental results showed that the amount of β‐carotene in the *S. angustifolia* and *A. mysorensis* were 1.72 ± 0.00 mg/g and 1.12 ± 0.00 mg/g, respectively. Overall *A. mysorensis* and *S. angustifolia* have a much higher β‐carotene content than other globally known species or varieties commonly produced and consumed as green leafy vegetables. Vitamin A is an essential nutrient in humans which plays a vital role in the functioning of the visual system, and maintenance of cell function for growth and epithelial cellular integrity as well as production of red blood cells (World Health Organization, 2009). The main sources of dietary vitamin A include provitamin A carotenoid such as β‐carotene, lutein, violaxanthin, and preformed retinol (mainly as retinyl ester). With regards to plant sources, vitamin A concentrations are dependent on β‐carotene content, its bioavailability, and bioefficacy (West, Eilander, & Van Lieshout, 2002). Dietary diversification through the intake of foods rich in provitamin A such as dark green leafy vegetables is one of the three ways in which prevalence of vitamin A deficiency can be curbed.

### 3.8 GC‐MS profile

The GC‐MS chromatogram of eluted compounds present in the methanolic extracts with their chemical structures and the retention time is depicted in Tables 4 and 5, (Appendix S1 and S2) respectively.

**Table 4:** GC‐MS peak identification for *S. angustofolia*

Retention time	Area	% Peak	Compound	Molecular formulae	Nature of compound
4.82	23,803	1.92	1,1‐Ethanediol, diacetate	C_6_H_10_O_4_	Ester
5.593	49,915	4.03	Glycerin	C_3_H_8_O_3_	Alcohol
5.68	22,794	1.84	Octane, 2,7‐dimethyl	C_10_H_22_	Alkane
5.887	44,304	3.57	But‐3‐enyl (E)‐2‐methylbut‐2‐enoate	C_5_H_7_O_2_	Ester
6.147	28,559	2.3	2,3‐Butanediol, diacetate	C_8_H_14_O_4_	Ester
6.273	25,896	2.09	2,3‐Butanediol, diacetate	C_8_H_14_O_4_	Ester
6.697	48,231	3.89	2‐Butenoic acid, 2‐methyl‐, 2‐methyl‐2‐propenyl ester, (E)‐	C_9_H_14_O_2_	Ester
7.053	22,916	1.85	2,4‐Pentanediol, 3‐nitro‐, diacetate	C_9_H_19_NO_8_	Alcohol
7.307	31,665	2.55	Ethyl 4‐acetoxybutanoate	C_8_H_14_O_4_	Ester
7.367	37,014	2.99	2‐Pyrrolidinone, 5‐(hydroxymethyl)‐	C_5_H_9_NO_2_	Ketone
7.71	26,964	2.17	4‐Penten‐2‐ol, acetate	C_7_H_12_O_2_	Ester
8.183	21,238	1.71	Butanoic acid, 3‐oxo‐, 1‐methylpropyl ester	C_8_H_14_O_3_	Fatty acid ester
10.28	28,065	2.26	phenol, 2‐(1,1,3,3‐tetramethylbutyl)‐	C_14_H_22_O	Alcohol
11.753	67,781	5.47	Ethanol, 2‐[4‐(1,1‐dimethylethyl)phenoxy]‐	C_12_H_18_O_2_	Alcohol
11.81	18,985	1.53	(3S,3aS,5R,6S,7aS)‐3,6,7,7‐Tetramethyloctahydro‐3a,6‐ethanoinden‐5‐ol	C_15_H_26_O	Alcohol
12.263	21,813	1.76	Decane, 1,1'‐oxybis‐	C_20_H_42_O	Ether
13.193	73,646	5.94	Ethanol, 2‐[2‐[4‐(1,1,3,3‐tetramethylbutyl)phenoxy]ethoxy]‐	C_18_H_30_O_3_	Alcohol
13.243	21,881	1.76	1,8‐Dimethyl‐3,6‐diazahomoadamantan‐9‐ol	C_13_H_24_N_2_O	Alcohol
13.813	59,859	4.83	2‐Methylhexacosane	C_27_H_56_	Hydrocarbons
14.39	176,838	14.26	Triacontane, 1‐iodo‐	C_30_H_62_	Hydrocarbon
14.447	77,928	6.28	Ethanol, 2‐[2‐[2‐[4‐(1,1,3,3‐tetramethylbutyl)phenoxy]ethoxy]ethoxy]‐	C_22_H_38_O_5_	Alcohol
16.193	181,381	14.63	Carbonic acid, eicosyl prop‐1‐en‐2‐yl ester	C_24_H_46_O_3_	Ester

**Table 5:** GC‐MS peak identification of *Asystasia mysorensis*

Retention time	Area	% Area	Identity	Molecular Formula	Nature of compound
5.433	18,909	1.44	Hexanoic acid	CH_3_(CH_2_)_4_COOH	Carboxylic acid
5.552	434,841	33.15	Glycerin	C_3_H_8_O_3_	Alcohol
6.476	32,093	2.44	2‐Pyrrolidinone	C_4_H_7_NO	Ketone
6.723	16,338	1.24	1,3‐Butanediol, diacetate	C_8_H_14_O_4_	Ester
6.997	16,079	1.22	3‐Amino‐1‐propanol, N,O‐diacetyl‐	C_7_H_13_NO_3_	Alcohol
7.029	17,227	1.31	Cyclopropylmethanol acetate	C_6_H_10_O_2_	Ester
7.319	85,218	6.49	5‐Methoxypyrrolidin‐2‐one	C_5_H_9_NO_2_	Ketone
9.185	46,994	3.58	Ethanol, 2‐(1‐methylethoxy)‐, acetate	C_7_H_14_O_3_	Ester
9.750	15,274	1.16	1,7‐Diazabicyclo[2.2.0]heptane, 7‐chloro‐	C_5_H_9_ClN_2_	Alkane
9.977	18,404	1.40	2(4H)‐Benzofuranone, 5,6,7,7a‐tetrahydro‐4,4,7a‐trimethyl‐	C_11_H_16_O_2_	Ketone
10.155	20,415	1.56	3‐Deoxy‐d‐mannoic lactone	C_6_H_10_O_5_	Cyclic esther
10.260	23,636	1.80	Phenol, 4‐(1,1,3,3‐tetramethylbutyl)‐	C_14_H_22_O	Alcohol
10.379	16,338	1.24	2,9‐Heptadecadiene‐4,6‐diyn‐8‐ol, (Z,E)‐	C_17_H_24_O	Alcohol
10.784	17,605	1.34	Cyclopropanedecanoic acid, 2‐octyl‐, methyl ester	C_24_H_46_O_2_	Fatty acid ester
11.714	34,490	2.63	Ethanol, 2‐[4‐(1,1‐dimethylethyl)phenoxy]‐	C_12_H_18_O_2_	Alcohol
12.067	15,716	1.20	Heptadecanoic acid	C_17_H_34_O_2_	Fatty acid
12.800	209,145	15.93	Phytol	C_20_H_40_O	Acyclic diterpene
12.963	24,341	1.85	Tridecanoic acid	C_13_H_26_O_2_	Fatty acid
13.146	20,082	1.53	Ethanol, 2‐[2‐[4‐(1,1,3,3‐tetramethylbutyl)phenoxy]ethoxy]‐	C_18_H_30_O_3_	Alcohol
16.122	55,142	4.20	13‐Docosenamide, (Z)‐	C_22_H_43_NO	Amide ester
16.269	22,143	1.69	4‐(5‐Pentyl‐3a,4,5,7a‐tetrahydro‐4‐indanyl)butanoic acid, methyl ester(stereoisomer 1)	C_19_H_32_O_2_	Fatty acid ester

Plant‐derived therapeutic compounds belong to various classes of secondary metabolites that have a wide range of activity which is dependent on the species, climate of the country of origin, the topography and may contain different categories of active components (Tamokou et al., 2017). The GC‐MS chromatogram of *S. angustofolia* and *A. mysorensis* confirmed the presence of several components belonging to different classes of compounds as depicted in Tables 3 and 6 above, respectively. Phytol, a natural linear diterpene alcohol used in the preparation of vitamins E and K1 and a decomposition product of chlorophyll (Peisker, Düggelin, Rentsch, & Matile, 1989), was detected in extracts of both plants. In a study done by Itoh et al. (2018), the phytol isolated from watermelon (*Citrullus lanatus*) sprouts inhibited the growth of a human T‐cell leukemia line Jurkat cell and suppressed tumor progression in a xenograft model of human lung adenocarcinoma epithelial cell line A549 in nude mice (Itoh et al., 2018). Hexanoic acid is a short natural monocarboxylic acid present in some fruits and plants reported to be an effective treatment against several pathogens in tomato plants (Llorens, Camanes, Lapena, & Garca‐Agustin, 2016)**.** Glycerin decreases intracranial pressure in numerous disease states, including stroke, encephalitis, Reye's syndrome, meningitis, pseudo‐central nervous system tumor, tumor cerebri, and space occupying lesions. It is also effective in lowering intraocular pressure in glaucoma and shrinking the brain during neurosurgical procedures (Frank, Nahata, & Hilty, 1981; Ghosh et al., 2015). The compound, 3‐deoxy‐d‐mannoic lactone with % peak area (10.155), has previously been reported to have antibacterial activity (Ghosh et al., 2015; Shobana, Vidhya, & Ramya, 2009).

**Table 6:** Mean concentrations (mg/kg) and standard deviations of toxic elements found in *Asystasia mysorensis* (mg/kg) and *Sesamum angustifolia* (mg/kg)

	*A. mysorensis* (mg/kg)	*S. angustifolia* (mg/kg)	PTWI (mg/kg/body weight)
Arsenic	0.22 ± 0.00	0.36 ± 0.01	0.015
Lead	34.44 ± 1.8	40.29 ± 1.4	0.025
Mercury	0.48 ± 0.0	0.33 ± 0.0	0.001

Abbreviation: TWI, Provisional tolerable weekly intake.

## 4 CONCLUSION

*Asystasia mysorensis* and *S. angustifolia have* nutritional potential that are worthy of exploitation as a dietary resource due to the presence of sufficient amounts of proteins, carbohydrates, fats, and minerals and other micronutrients. The presence of phenolic acids and flavonoids in these wild edible plants in varying amounts has enriched the nutraceutical properties of these plants. Dietary antioxidants such as phenolic compounds provide bioactive mechanisms reported have the ability to reduce lifestyle‐related diseases (Hung et al., 2004; Uusiku et al., 2010). Moreover, the compounds identified from methanolic extract of *A. mysorensis* and *S. angustifolia* have been reported to possess biological activity and further isolation of these phytoconstituents may prove their medicinal importance in future. Encouraging the use of wild edible green leafy vegetables such as *A. mysorensis* and *S. angustifolia* needs to be realized by highlighting their importance in areas where they currently being produced and consumed (Baldermann et al., 2016).

## CONFLICT OF INTEREST

The authors declare that they do not have any conflict of interest.

## ETHICAL STATEMENT

This study does not involve any human or animal testing.
