# Supplementary material for: Evaluation of nutritional value of Asystasia mysorensis and Sesamum angustifolia and their potential contribution to human health
Source: Food Sci Nutr. 2019 May 15;7(6):2176–85. doi: 10.1002/fsn3.1064 (PMC6593372; doi:10.1002/fsn3.1064)

Library

<< Target >>

Line#:1   R.Time:4.793(Scan#:89) MassPeaks:249
RawMode:Averaged 4.790-4.797(88-90) BasePeak:43.05(2326)
BG Mode:Calc. from Peak Group 1 - Event 1 Scan

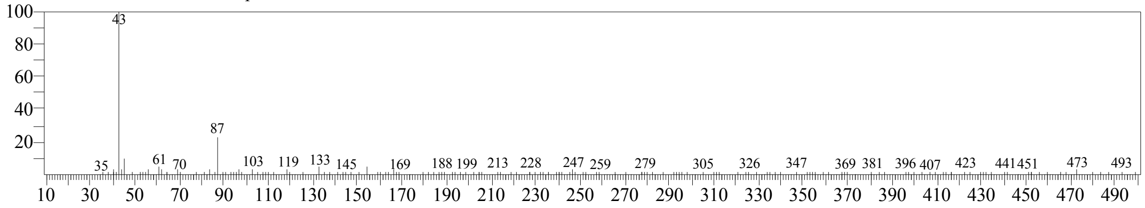

Hit#:1 Entry:39249 Library:NIST14.lib
SI:70 Formula:C8H14O5 CAS:10526-21-3 MolWeight:190 RetIndex:1099
CompName:Ethanol, 1,1'-oxybis-, diacetate $$ Ethanol, 1,1'-oxydi-, diacetate $$ di(1-Acetoxyethyl) ether $$ 1-[1-(Acetyloxy)ethoxy]ethyl acetate # $$

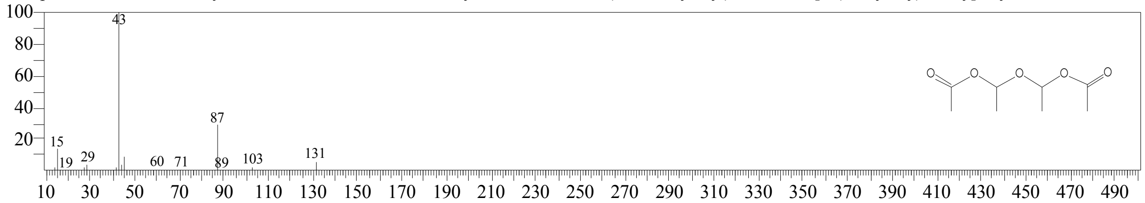

Hit#:2 Entry:13859 Library:NIST14.lib
SI:70 Formula:C6H10O4 CAS:542-10-9 MolWeight:146 RetIndex:888
CompName:1,1-Ethanediol, diacetate $$ Ethylidene acetate $$ Ethylidene diacetate $$ 1,1-Diacetoxyethane $$ 1,1'-Diacetoxy-ethane $$ 1-(Acetyloxy)ethyl

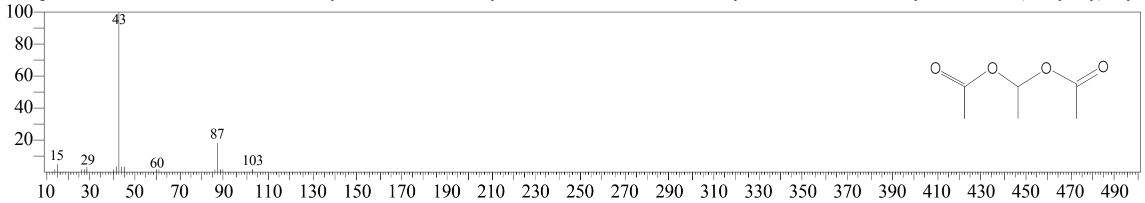

Hit#:3 Entry:7964 Library:NIST14.lib
SI:69 Formula:C6H10O3 CAS:4906-24-5 MolWeight:130 RetIndex:857
CompName:CH3C(O)OCH(CH3)C(O)CH3 $$ 2-Acetoxy-3-butanone $$

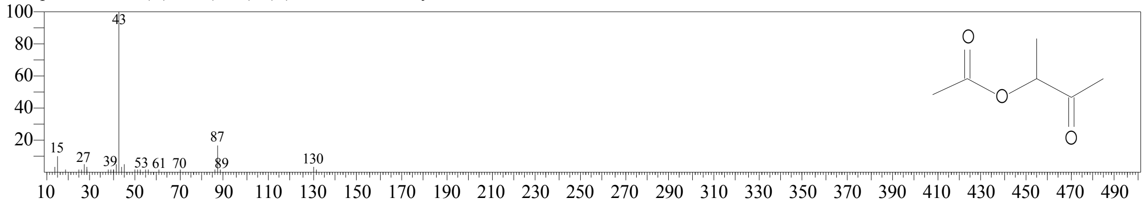

Hit#:4 Entry:15428 Library:NIST14.lib
SI:68 Formula:C6H11ClO2 CAS:54192-20-0 MolWeight:150 RetIndex:862
CompName:2-Butanol, 3-chloro-, acetate, (R*,R*)- $$ 2-Chloro-1-methylpropyl acetate # $$

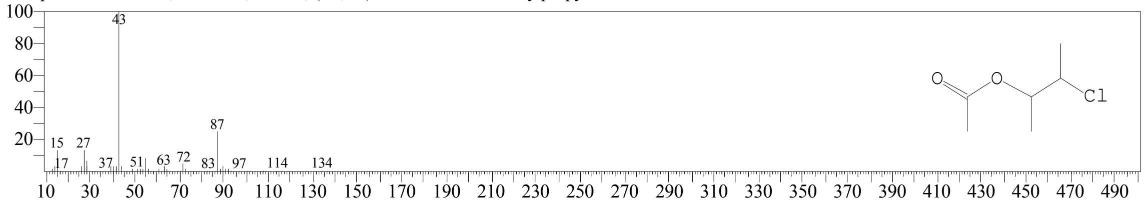

Hit#:5 Entry:4611 Library:NIST14.lib
SI:67 Formula:C6H12O2 CAS:105-46-4 MolWeight:116 RetIndex:721
CompName:sec-Butyl acetate $$ Acetic acid, 1-methylpropyl ester $$ Acetic acid, sec-butyl ester $$ sec-Butyl alcohol acetate $$ 2-Butyl acetate $$ CH3CC

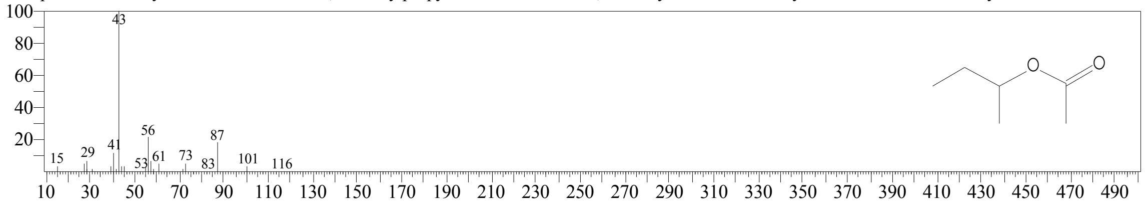

<< Target >>
Line#:2 R.Time:5.553(Scan#:317) MassPeaks:274
RawMode:Averaged 5.550-5.557(316-318) BasePeak:61.05(31297)
BG Mode:Calc. from Peak Group 1 - Event 1 Scan

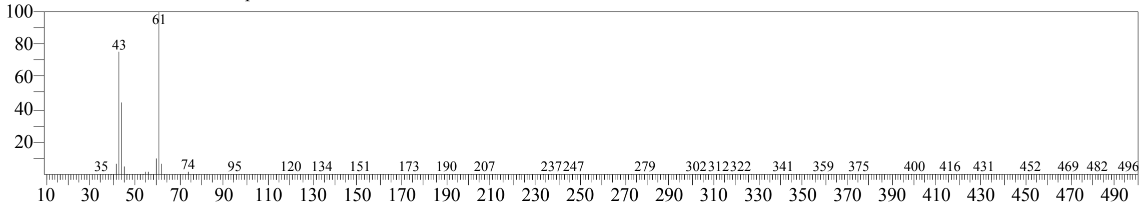

Hit#:1 Entry:1200 Library:NIST14.lib
SI:96 Formula:C3H8O3 CAS:56-81-5 MolWeight:92 RetIndex:967
CompName:Glycerin $$ 1,2,3-Propanetriol $$ Glycerol $$ Glycerine $$ Glyceritol $$ Glycyl alcohol $$ Glyrol $$ Glysanin $$ Osmoglyn $$ Propanetriol $

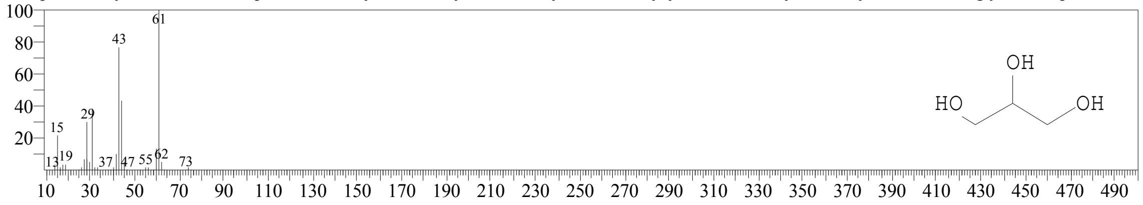

Hit#:2 Entry:1126 Library:NIST14.lib
SI:86 Formula:C3H6O3 CAS:56-82-6 MolWeight:90 RetIndex:913
CompName:Glyceraldehyde $$ dl-Glyceraldehyde $$ Propanal, 2,3-dihydroxy-, (.+/-.)- $$ Glyceraldehyde, dl- $$ Glyceraldehyde, (.+/-.)- $$ DL-Glyceric al

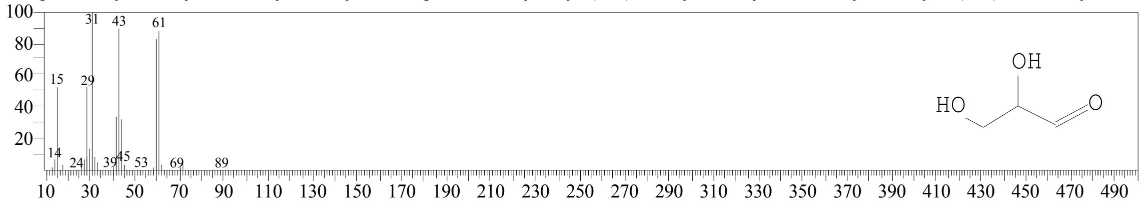

Hit#:3 Entry:2413 Library:NIST14.lib
SI:85 Formula:C4H9NO2 CAS:78191-00-1 MolWeight:103 RetIndex:696
CompName:N-Methoxy-N-methylacetamide $$ N-Methyl-N-methoxyacetamide $$

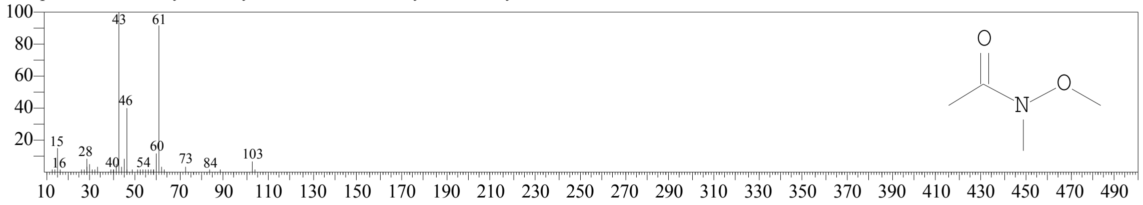

Hit#:4 Entry:151 Library:NIST14.lib
SI:84 Formula:C2H7NO CAS:624-86-2 MolWeight:61 RetIndex:0
CompName:o-Ethylhydroxylamine $$ Hydroxylamine, O-ethyl- $$

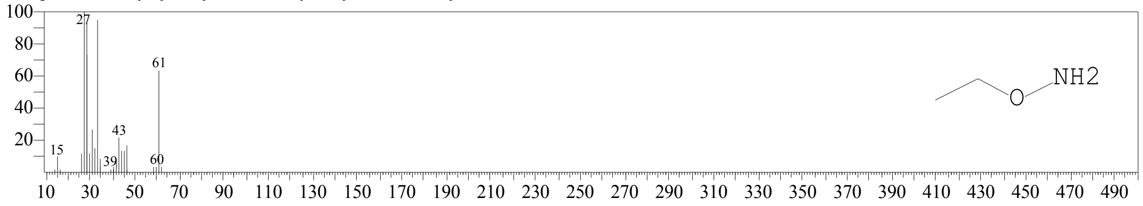

Hit#:5 Entry:4579 Library:NIST14.lib
SI:83 Formula:C5H12N2O CAS:57536-14-8 MolWeight:116 RetIndex:796
CompName:O-Butylisourea $$ Butyl imidocarbamate # $$

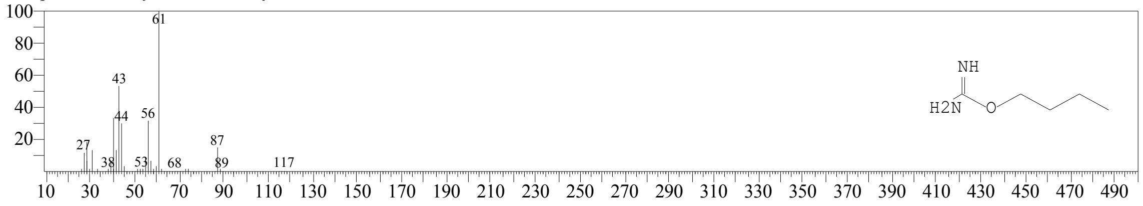

<< Target >>
Line#:3 R.Time:6.473(Scan#:593) MassPeaks:255
RawMode:Averaged 6.470-6.477(592-594) BasePeak:85.10(4213)
BG Mode:Calc. from Peak Group 1 - Event 1 Scan

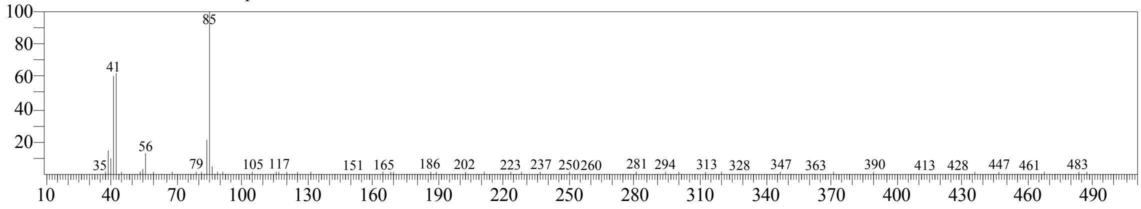

Hit#:1 Entry:770 Library:NIST14.lib
SI:89 Formula:C4H7NO CAS:616-45-5 MolWeight:85 RetIndex:763
CompName:2-Pyrrolidinone $$ .gamma.-Aminobutyrolactam $$ .alpha.-Pyrrolidinone $$ .alpha.-Pyrrolidone $$ .gamma.-Aminobutyric lactam $$ .gamma.-

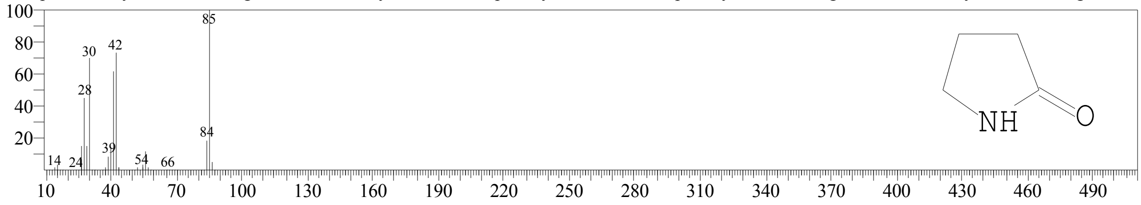

Hit#:2 Entry:11799 Library:NIST14.lib
SI:79 Formula:C6H7NO3 CAS:182881-06-7 MolWeight:141 RetIndex:1172
CompName:Cyclobutene-3,4-dione, 1-dimethylamino-2-hydroxy- $$ 3-(Dimethylamino)-4-hydroxy-3-cyclobutene-1,2-dione # $$

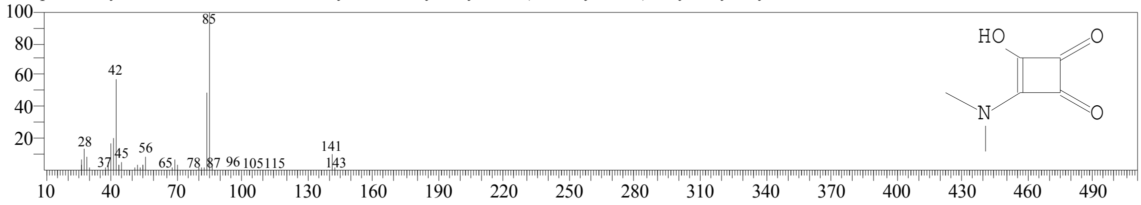

Hit#:3 Entry:751 Library:NIST14.lib
SI:75 Formula:C2H3N3O CAS:930-33-6 MolWeight:85 RetIndex:1012
CompName:3H-1,2,4-Triazol-3-one, 1,2-dihydro- $$ s-Triazol-3-ol $$ 2,4-Dihydro-3H-1,2,4-triazol-3-one # $$

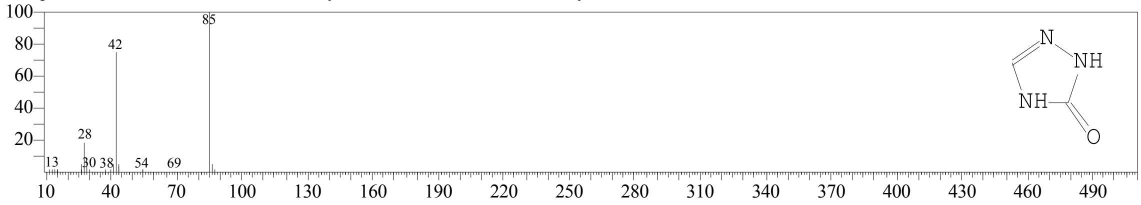

Hit#:4 Entry:70699 Library:NIST14.lib
SI:74 Formula:C13H26O3 CAS:7523-15-1 MolWeight:230 RetIndex:1557
CompName:Carbonic acid, dihexyl ester $$ Dihexyl carbonate $$ (n-C6H13)OC(O)O(n-C6H13) $$ Di-n-hexyl carbonate $$

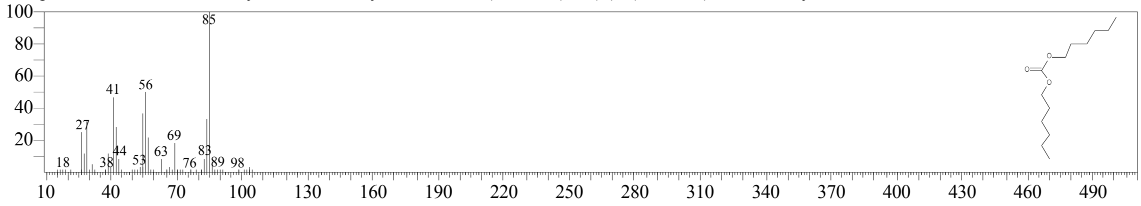

Hit#:5 Entry:31287 Library:NIST14.lib
SI:73 Formula:C6H11BrO CAS:34723-82-5 MolWeight:178 RetIndex:1066
CompName:2H-Pyran, 2-(bromomethyl)tetrahydro- $$ 2-(Bromomethyl)tetrahydro-2H-pyran $$ Pyran, 2-(bromomethyl)tetrahydro- $$

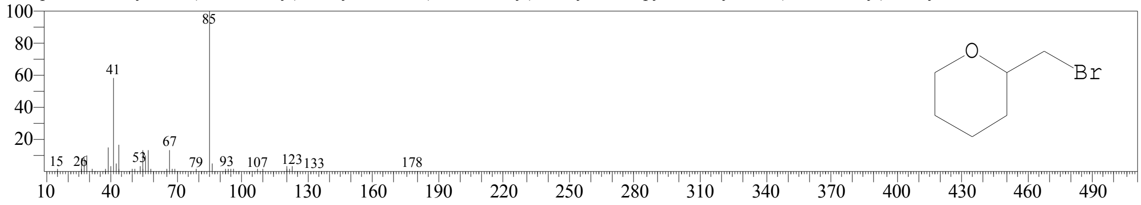

<< Target >>
Line#:4 R.Time:6.653(Scan#:647) MassPeaks:231
RawMode:Averaged 6.650-6.657(646-648) BasePeak:83.00(2912)
BG Mode:Calc. from Peak Group 1 - Event 1 Scan

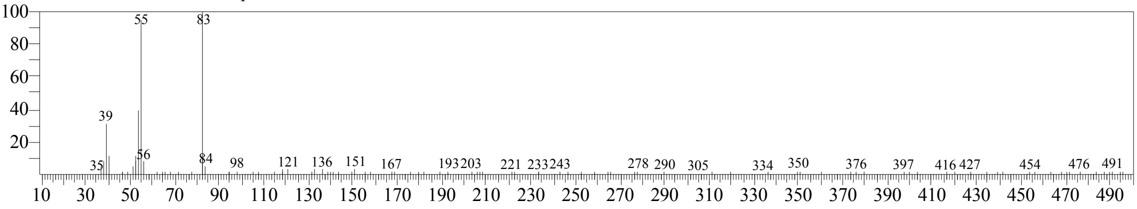

Hit#:1 Entry:17751 Library:NIST14.lib
SI:80 Formula:C9H14O2 CAS:61692-78-2 MolWeight:154 RetIndex:0
CompName:2-Butenoic acid, 2-methyl-, 2-methyl-2-propenyl ester, (Z)-

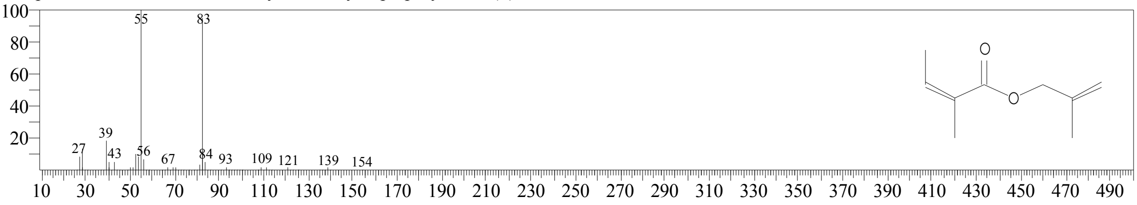

Hit#:2 Entry:4958 Library:NIST14.lib
SI:77 Formula:C5H7ClO CAS:3350-78-5 MolWeight:118 RetIndex:816
CompName:3,3-Dimethylacryloyl chloride $$ 3,3-Dimethylacrylyl chloride $$ 2-Butenoyl chloride, 3-methyl- $$ 3-Methyl-2-butenoyl chloride # $$

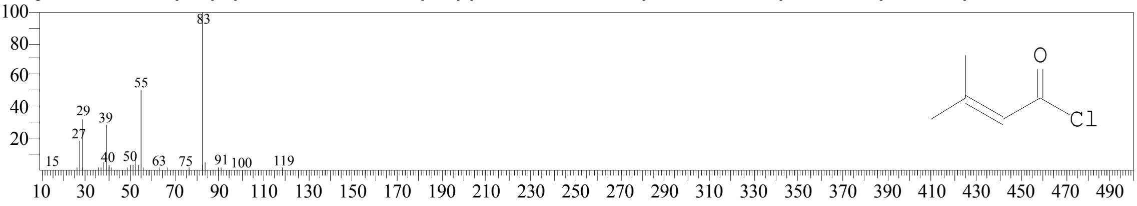

Hit#:3 Entry:17802 Library:NIST14.lib
SI:77 Formula:C9H14O2 CAS:61692-82-8 MolWeight:154 RetIndex:0
CompName:2-Butenoic acid, 2-methyl-, 2-methyl-2-propenyl ester, (E)-

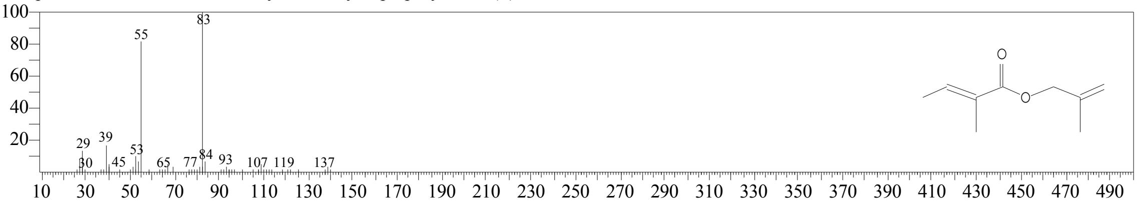

Hit#:4 Entry:17804 Library:NIST14.lib
SI:77 Formula:C9H14O2 CAS:0-00-0 MolWeight:154 RetIndex:1058
CompName:But-3-enyl (E)-2-methylbut-2-enoate

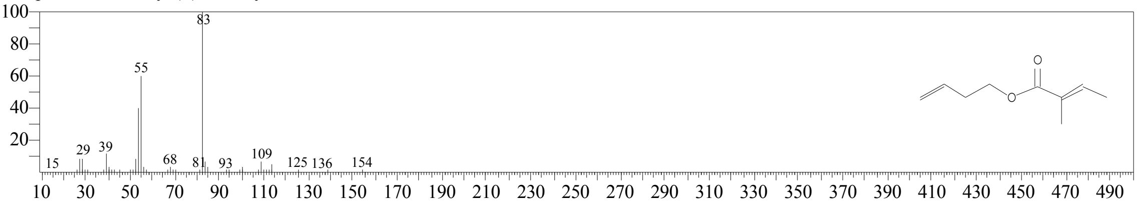

Hit#:5 Entry:105658 Library:NIST14.lib
SI:76 Formula:C11H10BrFO2 CAS:0-00-0 MolWeight:272 RetIndex:1638
CompName:3-Methyl-2-butenoic acid, 2-bromo-4-fluorophenyl ester $$ 2-Bromo-4-fluorophenyl 3-methyl-2-butenoate # $$

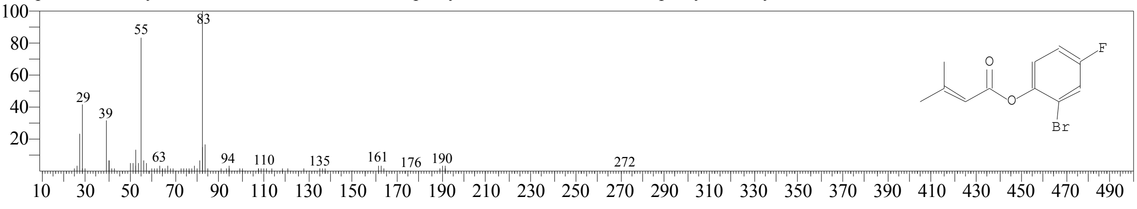

<< Target >>
Line#:5 R.Time:6.723(Scan#:668) MassPeaks:252
RawMode:Averaged 6.720-6.727(667-669) BasePeak:43.00(4592)
BG Mode:Calc. from Peak Group 1 - Event 1 Scan

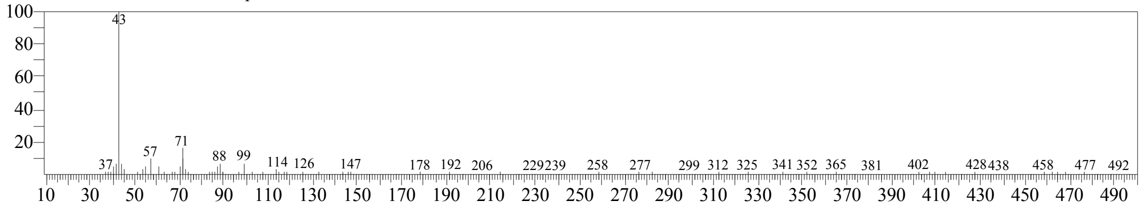

Hit#:1 Entry:29102 Library:NIST14.lib
SI:87 Formula:C8H14O4 CAS:1117-31-3 MolWeight:174 RetIndex:1087
CompName:1,3-Butanediol, diacetate $$ 1,3-Butylene diacetate $$ 1,3-Butylene glycol diacetate $$ 1,3-Diacetoxybutane $$ 1,3-Butandiol diacetate $$ 3-(

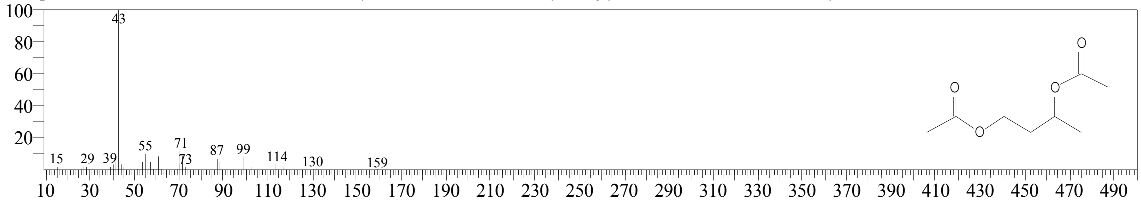

Hit#:2 Entry:29247 Library:NIST14.lib
SI:82 Formula:C9H18O3 CAS:204652-53-9 MolWeight:174 RetIndex:1095
CompName:Isopentyloxyethyl acetate $$ 2-(Isopentyloxy)ethyl acetate # $$ 2-Isopentoxyethyl acetate $$

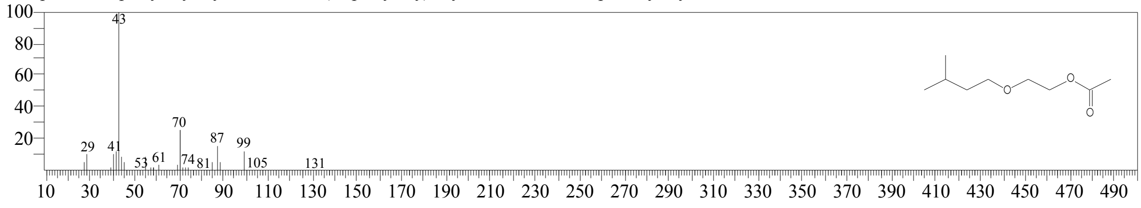

Hit#:3 Entry:8065 Library:NIST14.lib
SI:81 Formula:C7H14O2 CAS:89975-71-3 MolWeight:130 RetIndex:929
CompName:4-Propoxy-2-butanone

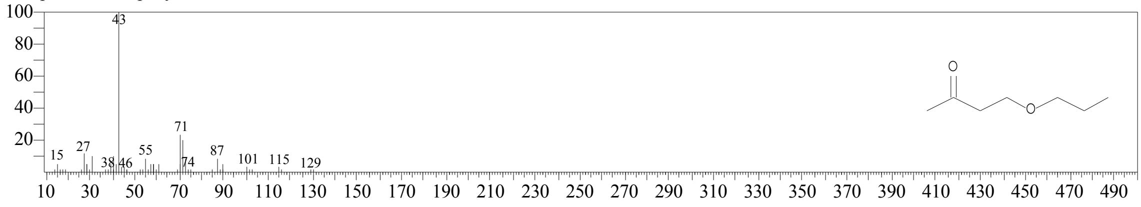

Hit#:4 Entry:29246 Library:NIST14.lib
SI:80 Formula:C9H18O3 CAS:5312-09-4 MolWeight:174 RetIndex:1159
CompName:Ethanol, 2-(pentyloxy)-, acetate $$ 2-(Pentyloxy)ethyl acetate $$ 2-Pentoxyethyl acetate $$

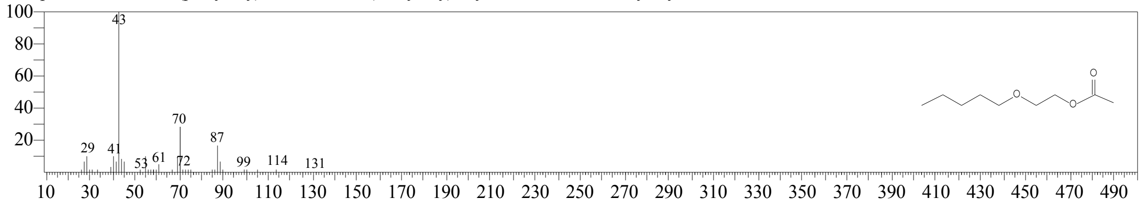

Hit#:5 Entry:29099 Library:NIST14.lib
SI:80 Formula:C8H14O4 CAS:628-67-1 MolWeight:174 RetIndex:1151
CompName:1,4-Butanediol, diacetate $$ Butylene glycol diacetate $$ Tetramethylene acetate $$ Tetramethylene diacetate $$ 1,4-Butylene glycol diacetate

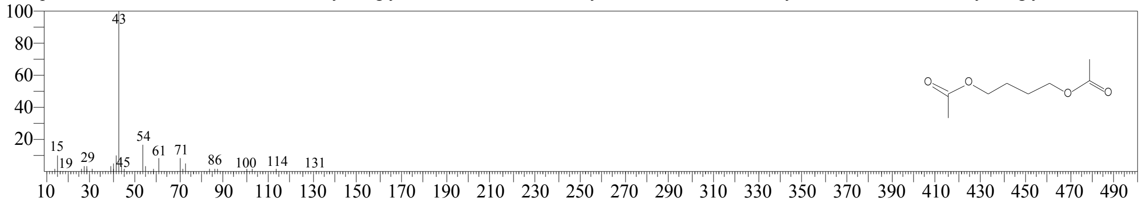

<< Target >>
Line#:6 R.Time:7.027(Scan#:759) MassPeaks:250
RawMode:Averaged 7.023-7.030(758-760) BasePeak:43.05(2045)
BG Mode:Calc. from Peak Group 1 - Event 1 Scan

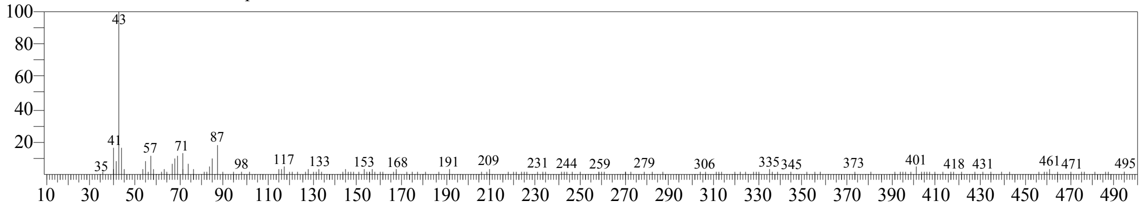

Hit#:1 Entry:7565 Library:NIST14.lib
SI:73 Formula:C9H20 CAS:1069-53-0 MolWeight:128 RetIndex:724
CompName:Hexane, 2,3,5-trimethyl- $$ 2,3,5-Trimethylhexane $$

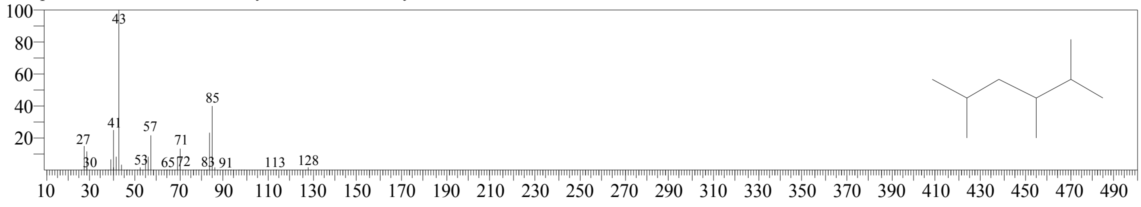

Hit#:2 Entry:7275 Library:NIST14.lib
SI:72 Formula:C7H12O2 CAS:0-00-0 MolWeight:128 RetIndex:810
CompName:4-Penten-2-ol, acetate

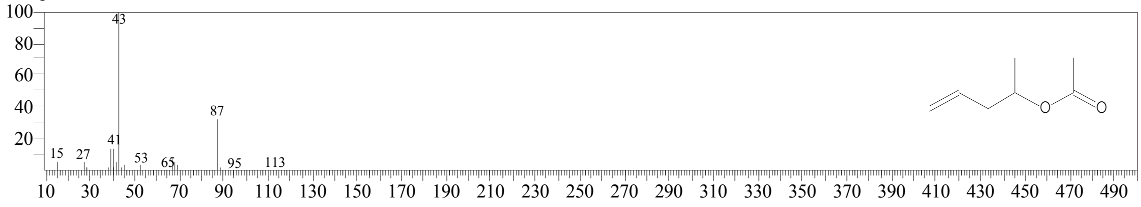

Hit#:3 Entry:81857 Library:NIST14.lib
SI:72 Formula:C13H24O4 CAS:0-00-0 MolWeight:244 RetIndex:1584
CompName:Oxalic acid, isohexyl pentyl ester

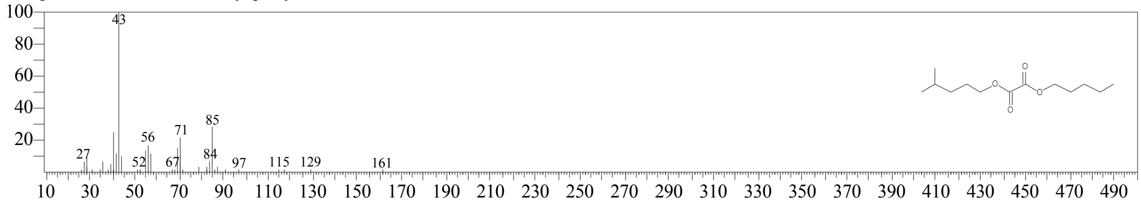

Hit#:4 Entry:928 Library:NIST14.lib
SI:71 Formula:C4H9NO CAS:40499-83-0 MolWeight:87 RetIndex:933
CompName:3-Pyrrolidinol $$ 3-Hydroxypyrrolidine $$

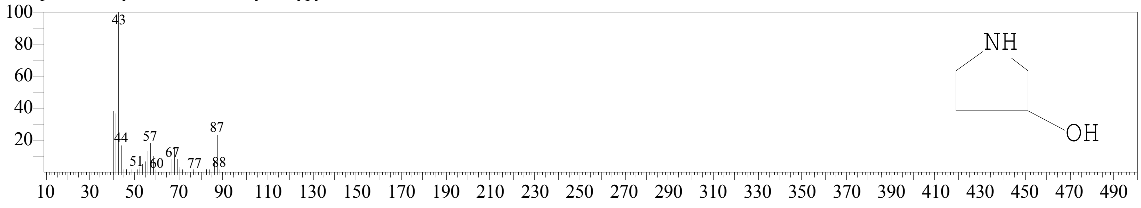

Hit#:5 Entry:7440 Library:NIST14.lib
SI:71 Formula:C8H16O CAS:24230-08-8 MolWeight:128 RetIndex:910
CompName:Cyclopropanemethanol, .alpha.-methyl-.alpha.-propyl- $$ 2-Pentanol, 2-cyclopropyl- $$ 2-Cyclopropyl-2-pentanol $$

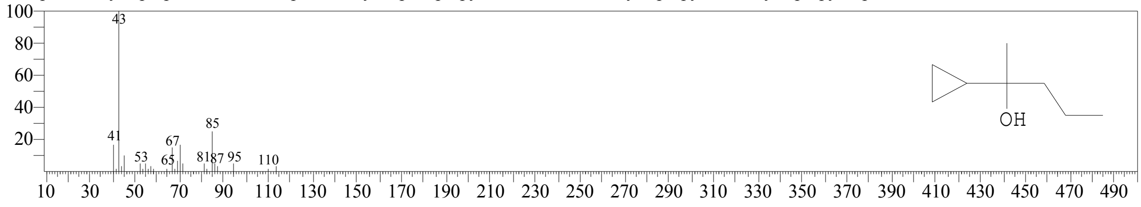

<< Target >>
Line#:7 R.Time:7.317(Scan#:846) MassPeaks:287
RawMode:Averaged 7.313-7.320(845-847) BasePeak:84.05(9458)
BG Mode:Calc. from Peak Group 1 - Event 1 Scan

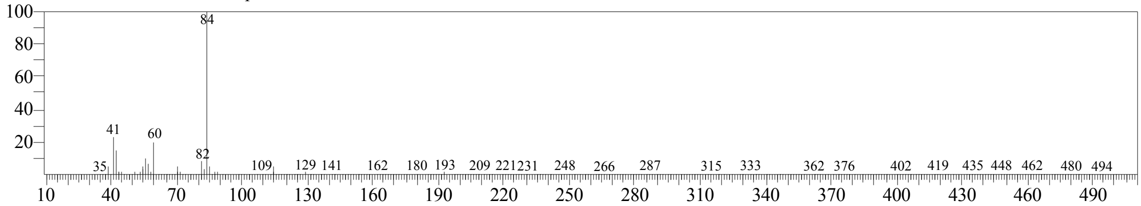

Hit#:1 Entry:4370 Library:NIST14.lib
SI:88 Formula:C5H9NO2 CAS:63853-74-7 MolWeight:115 RetIndex:900
CompName:5-Methoxypyrrolidin-2-one $$ 5-Methoxy-2-pyrrolidinone # $$

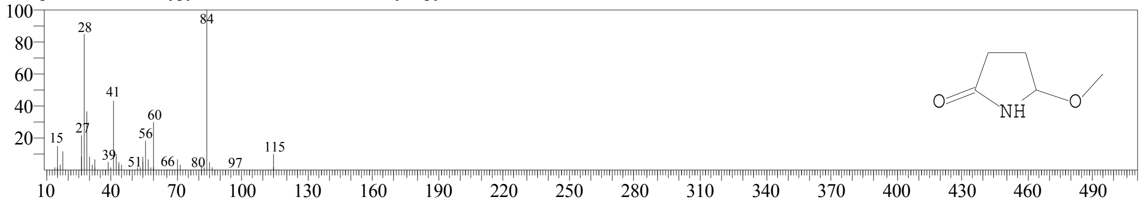

Hit#:2 Entry:4371 Library:NIST14.lib
SI:86 Formula:C5H9NO2 CAS:62400-75-3 MolWeight:115 RetIndex:1067
CompName:2-Pyrrolidinone, 5-(hydroxymethyl)- $$ Pyrrolid-2-one-5-methanol $$ 5-(Hydroxymethyl)-2-pyrrolidinone # $$

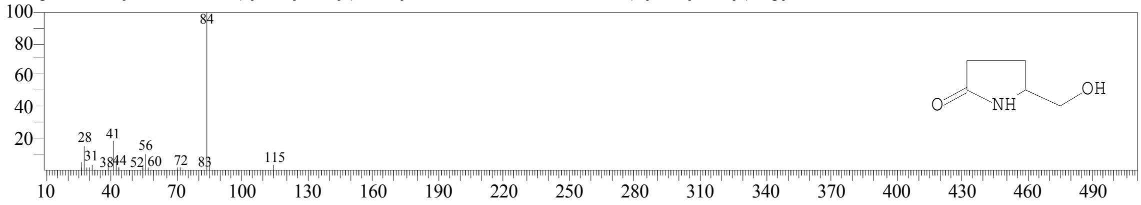

Hit#:3 Entry:12722 Library:NIST14.lib
SI:84 Formula:C6H9NO3 CAS:54571-66-3 MolWeight:143 RetIndex:1091
CompName:DL-Proline, 5-oxo-, methyl ester $$ Methyl 5-oxo-2-pyrrolidinecarboxylate # $$ 2-Pyrrolidone-5-carboxylic acid, methyl ester $$

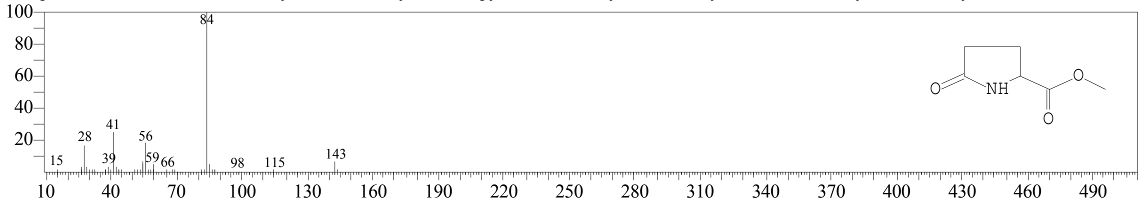

Hit#:4 Entry:12721 Library:NIST14.lib
SI:84 Formula:C6H9NO3 CAS:4931-66-2 MolWeight:143 RetIndex:1091
CompName:L-Proline, 5-oxo-, methyl ester $$ Proline, 5-oxo-, methyl ester $$ (S)-5-(Methoxycarbonyl)-2-pyrrolidone $$ Methyl pyroglutamate $$ Methyl

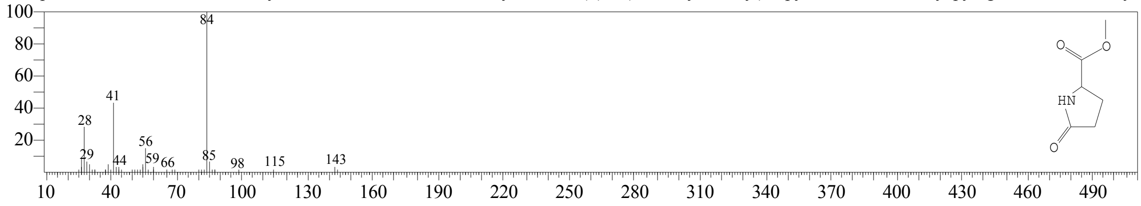

Hit#:5 Entry:12775 Library:NIST14.lib
SI:84 Formula:C7H13NO2 CAS:80243-73-8 MolWeight:143 RetIndex:1265
CompName:Pyrrolidine-5-one, 2-[3-hydroxypropyl]- $$ Pyrrolidin-5-one, 2-[3-hydroxypropyl]- $$ 5-(3-Hydroxypropyl)-2-pyrrolidinone # $$

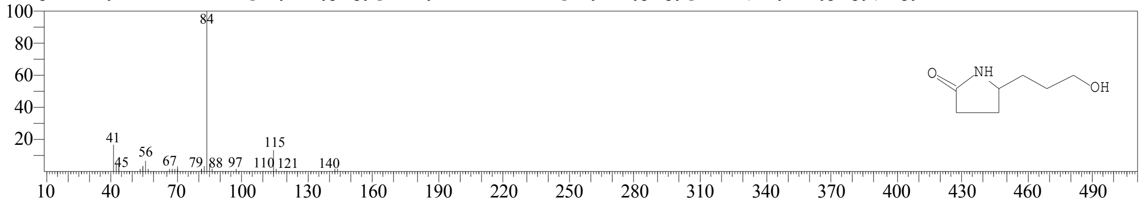

<< Target >>
Line#:8 R.Time:8.160(Scan#:1099) MassPeaks:278
RawMode:Averaged 8.157-8.163(1098-1100) BasePeak:43.05(2875)
BG Mode:Calc. from Peak Group 1 - Event 1 Scan

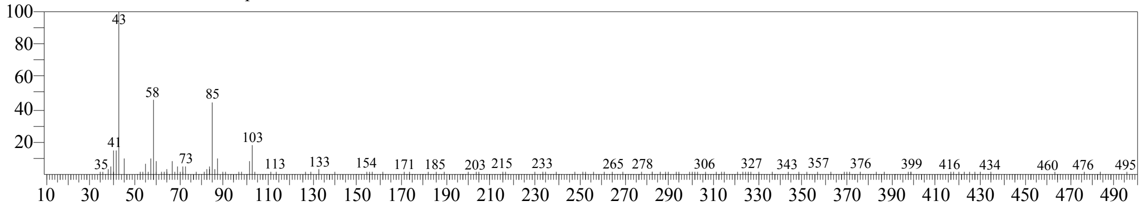

Hit#:1 Entry:19879 Library:NIST14.lib
SI:81 Formula:C8H14O3 CAS:13562-76-0 MolWeight:158 RetIndex:1056
CompName:Butanoic acid, 3-oxo-, 1-methylpropyl ester $$ Acetoacetic acid, sec-butyl ester $$ sec-Butyl Acetoacetate $$ sec-Butyl 3-oxobutanoate # $$

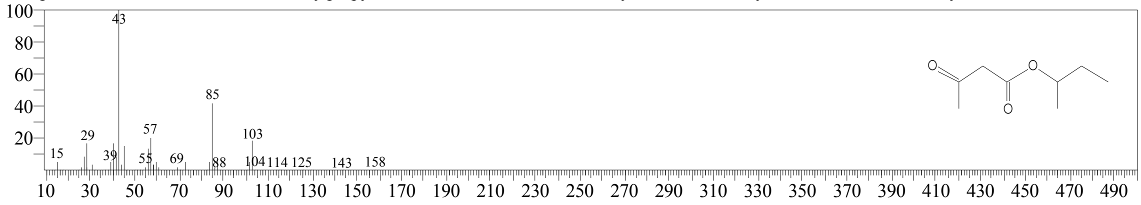

Hit#:2 Entry:28090 Library:NIST14.lib
SI:80 Formula:C9H16O3 CAS:6830-12-2 MolWeight:172 RetIndex:1155
CompName:1-Methylbutyl acetoacetate $$ 1-Methylbutyl 3-oxobutanoate # $$

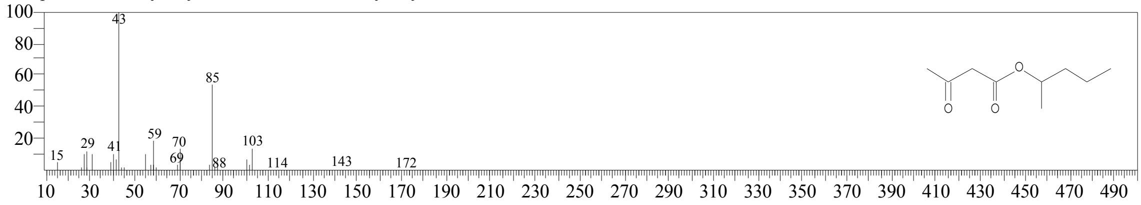

Hit#:3 Entry:8069 Library:NIST14.lib
SI:79 Formula:C7H14O2 CAS:19889-37-3 MolWeight:130 RetIndex:989
CompName:Butanoic acid, 2-ethyl-2-methyl- $$ Butyric acid, 2-ethyl-2-methyl- $$ 2-Ethyl-2-methylbutanoic acid $$ 2-Methyl-2-ethylbutanoic acid $$

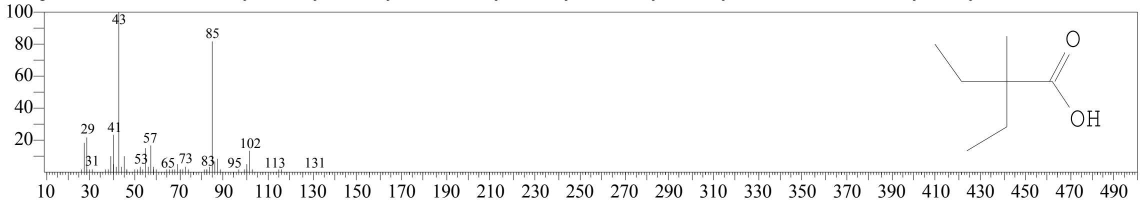

Hit#:4 Entry:13996 Library:NIST14.lib
SI:78 Formula:C7H14O3 CAS:0-00-0 MolWeight:146 RetIndex:1165
CompName:3-hydroxy-3-methyl-hexanoic acid

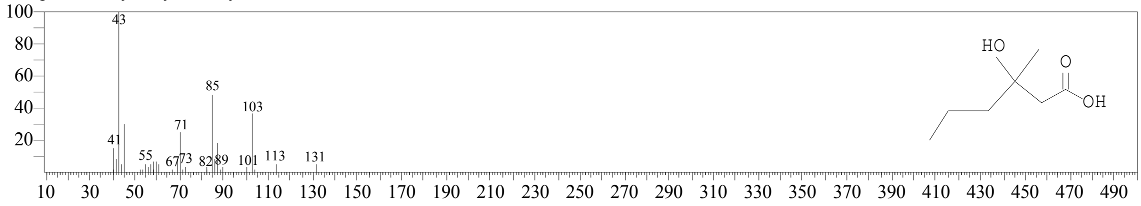

Hit#:5 Entry:28087 Library:NIST14.lib
SI:78 Formula:C9H16O3 CAS:6624-84-6 MolWeight:172 RetIndex:1219
CompName:Pentyl acetoacetate $$ Pentyl 3-oxobutanoate $$ Acetoacetic acid, n-amyl ester $$

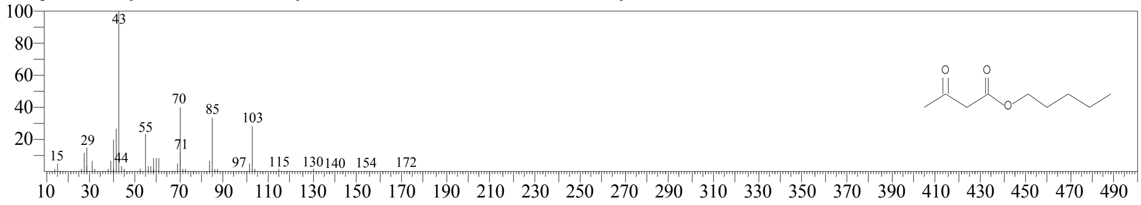

<< Target >>
Line#:9 R.Time:11.710(Scan#:2164) MassPeaks:255
RawMode:Averaged 11.707-11.713(2163-2165) BasePeak:179.10(3533)
BG Mode:Calc. from Peak Group 1 - Event 1 Scan

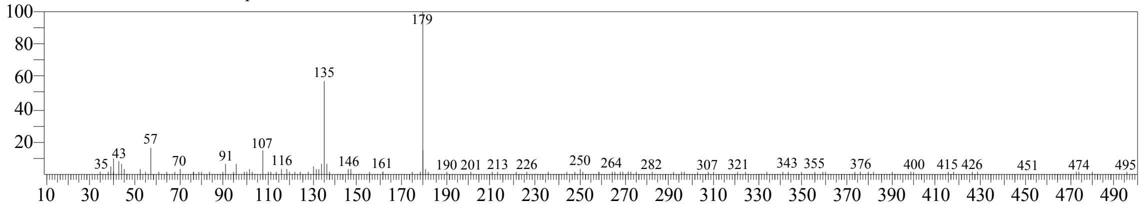

Hit#:1 Entry:42734 Library:NIST14.lib
SI:77 Formula:C12H18O2 CAS:713-46-2 MolWeight:194 RetIndex:1539
CompName:Ethanol, 2-[4-(1,1-dimethylethyl)phenoxy]- $$ Ethanol, 2-(p-tert-butylphenoxy)- $$ 2-(p-tert-Butylphenoxy)ethanol $$ 2-(p-tert-Butylphenyloxy

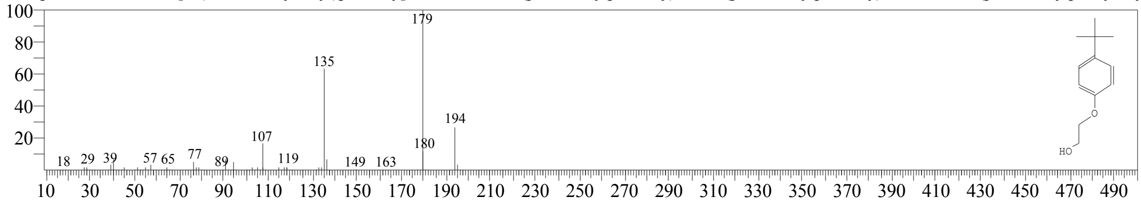

Hit#:2 Entry:53163 Library:NIST14.lib
SI:71 Formula:C13H20O2 CAS:6382-07-6 MolWeight:208 RetIndex:1638
CompName:Ethanol, 2-[4-(1,1-dimethylpropyl)phenoxy]- $$ Ethanol, 2-(p-tert-pentylphenoxy)- $$ 2-(p-tert-Amylphenoxy)ethanol $$ 2-(p-tert-Pentylpheno

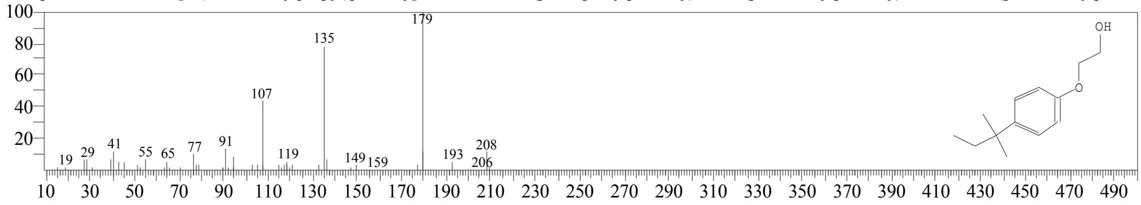

Hit#:3 Entry:42511 Library:NIST14.lib
SI:68 Formula:C11H14O3 CAS:36881-00-2 MolWeight:194 RetIndex:1475
CompName:1,3-Dioxolane, 2-(4-methoxyphenyl)-2-methyl- $$ Methyl 4-(2-methyl-1,3-dioxolan-2-yl)phenyl ether # $$

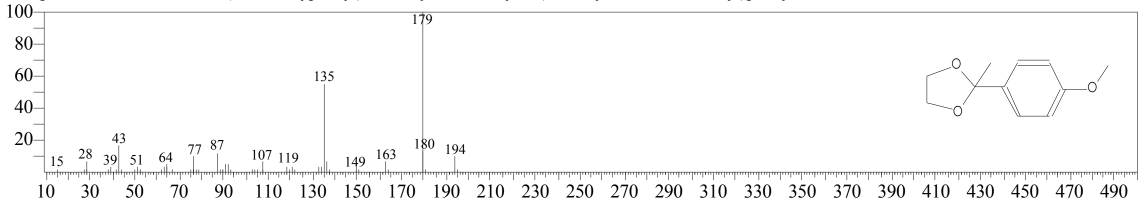

Hit#:4 Entry:76733 Library:NIST14.lib
SI:68 Formula:C12H14O5 CAS:0-00-0 MolWeight:238 RetIndex:1931
CompName:[2-(4-Methoxy-phenyl)-[1,3]dioxolan-2-yl]-acetic acid

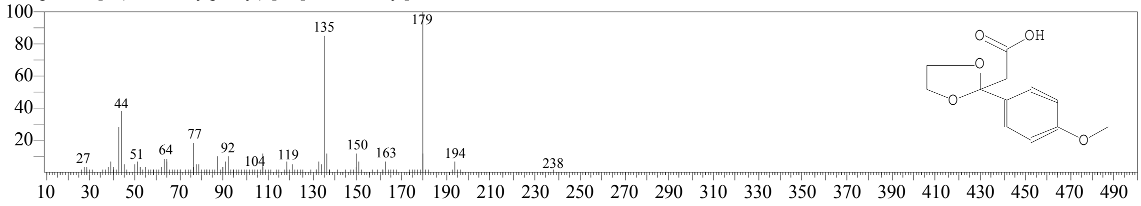

Hit#:5 Entry:42303 Library:NIST14.lib
SI:67 Formula:C10H14O2Si CAS:15290-29-6 MolWeight:194 RetIndex:0
CompName:Benzoic acid, 4-(trimethylsilyl)- $$ Benzoic acid, p-(trimethylsilyl)-, $$

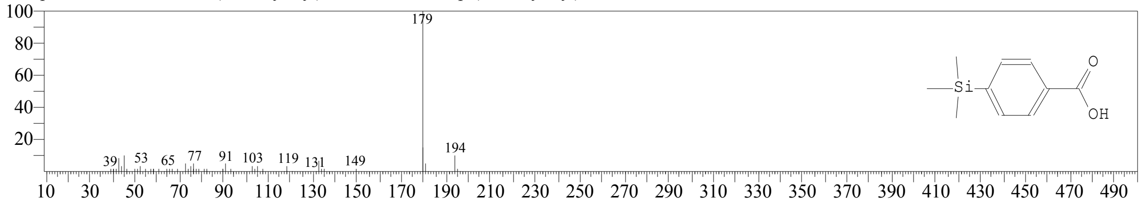

<< Target >>
Line#:10 R.Time:12.800(Scan#:2491) MassPeaks:276
RawMode:Averaged 12.797-12.803(2490-2492) BasePeak:71.05(12460)
BG Mode:Calc. from Peak Group 1 - Event 1 Scan

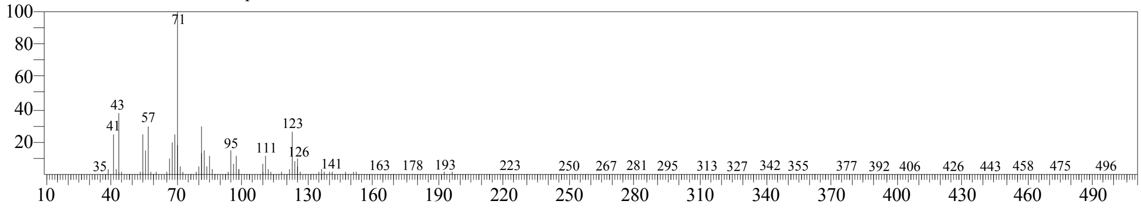

Hit#:1 Entry:127772 Library:NIST14.lib
SI:96 Formula:C20H40O CAS:150-86-7 MolWeight:296 RetIndex:2045
CompName:Phytol $$ 2-Hexadecen-1-ol, 3,7,11,15-tetramethyl-, [R-[R*,R*-(E)]]- $$ trans-Phytol $$ 3,7,11,15-Tetramethyl-2-hexadecen-1-ol-, (2E,7R,11I

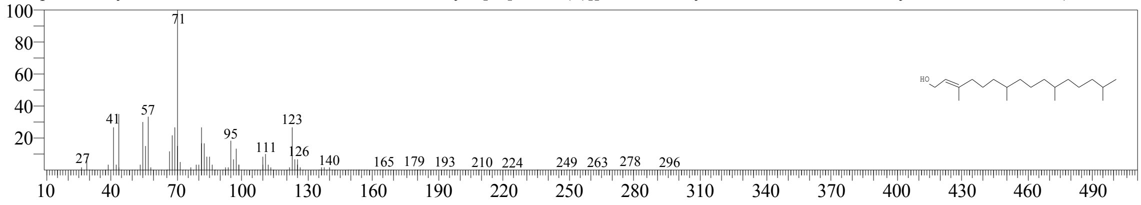

Hit#:2 Entry:19242 Library:NIST14.lib
SI:87 Formula:C10H20O CAS:23283-97-8 MolWeight:156 RetIndex:1164
CompName:Cyclohexanol, 5-methyl-2-(1-methylethyl)-, [1S-(1.alpha.,2.beta.,5.beta.)]- $$ (1S,2R,5R)-(+)-Isomenthol $$ 2-Isopropyl-5-methylcyclohexanol

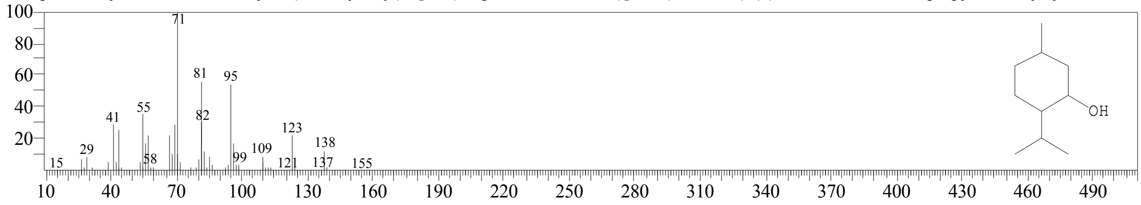

Hit#:3 Entry:19250 Library:NIST14.lib
SI:86 Formula:C10H20O CAS:491-01-0 MolWeight:156 RetIndex:1164
CompName:Cyclohexanol, 5-methyl-2-(1-methylethyl)-, (1.alpha.,2.alpha.,5.beta.)- $$ Menthol, trans-1,3,trans-1,4- $$ Neo-Menthol $$ Neomenthol $$ 2-Is

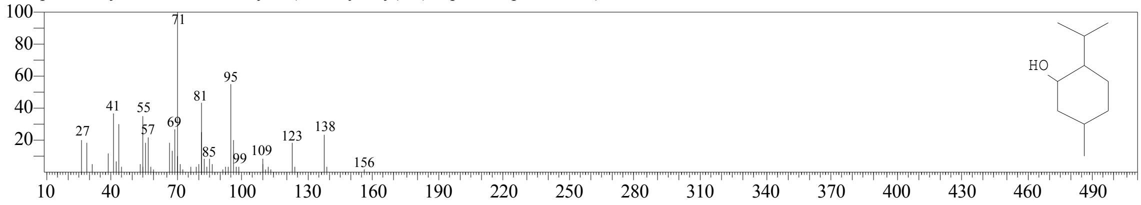

Hit#:4 Entry:142418 Library:NIST14.lib
SI:85 Formula:C21H44O CAS:0-00-0 MolWeight:312 RetIndex:0
CompName:Hexadecyl pentyl ether

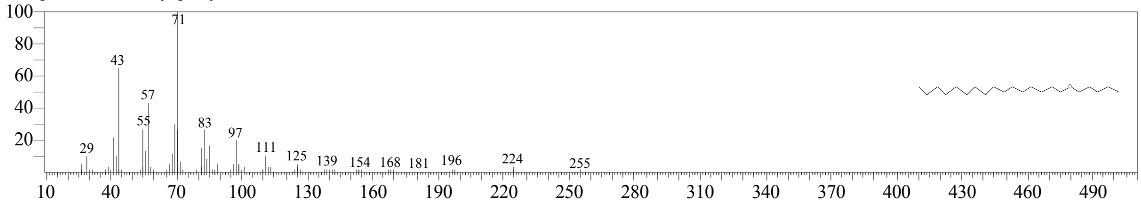

Hit#:5 Entry:117198 Library:NIST14.lib
SI:85 Formula:C19H40O CAS:0-00-0 MolWeight:284 RetIndex:0
CompName:Pentyl tetradecyl ether

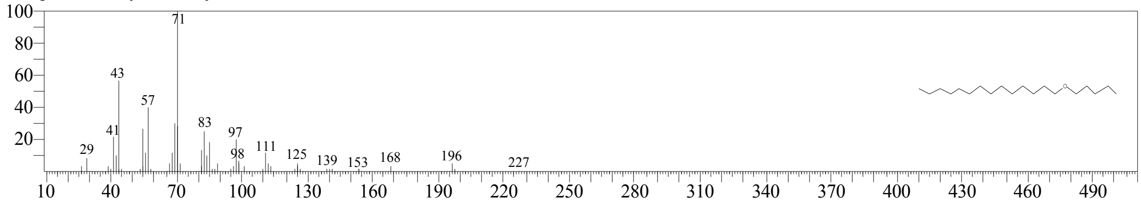

<< Target >>
Line#:11 R.Time:14.410(Scan#:2974) MassPeaks:297
RawMode:Averaged 14.407-14.413(2973-2975) BasePeak:73.00(1015)
BG Mode:Calc. from Peak Group 1 - Event 1 Scan

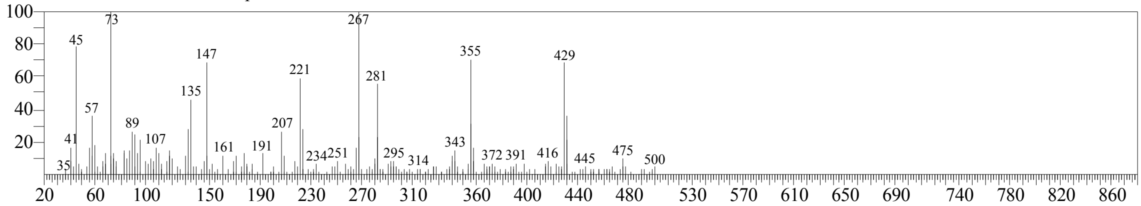

Hit#:1 Entry:221920 Library:NIST14.lib
SI:60 Formula:C13H40O5Si6 CAS:38147-00-1 MolWeight:444 RetIndex:1297
CompName:1,1,1,5,7,7,7-Heptamethyl-3,3-bis(trimethylsiloxy)tetrasiloxane $$ 1,3,3,3-Tetramethyldisiloxanyl tris(trimethylsilyl) orthosilicate # $$

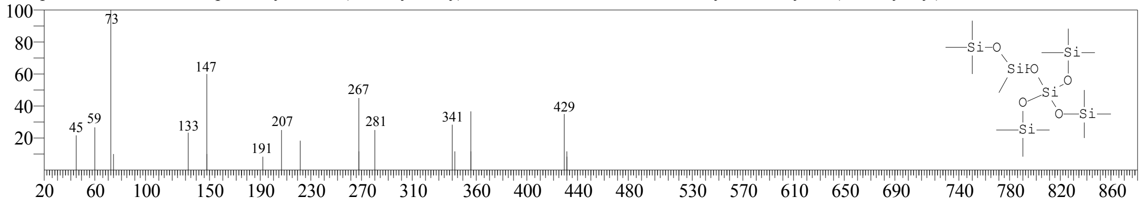

Hit#:2 Entry:240916 Library:NIST14.lib
SI:60 Formula:C18H54O9Si9 CAS:556-71-8 MolWeight:666 RetIndex:1860
CompName:Cyclononasiloxane, octadecamethyl- $$ Octadeamethyl-cyclononasiloxane $$ 2,2,4,4,6,6,8,8,10,10,12,12,14,14,16,16,18,18-Octadecamethylcy

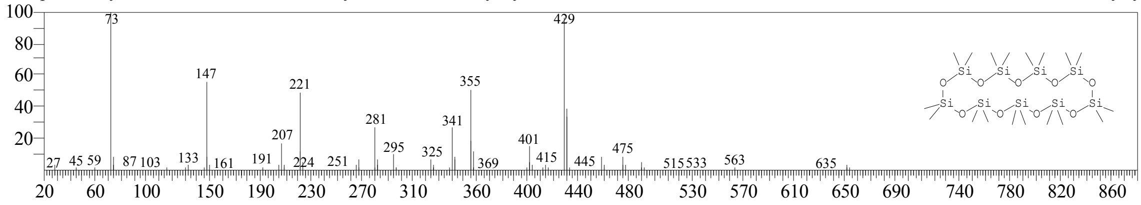

Hit#:3 Entry:242281 Library:NIST14.lib
SI:58 Formula:C24H72O12Si12 CAS:18919-94-3 MolWeight:888 RetIndex:2480
CompName:Tetracosamethyl-cyclododecasiloxane $$ 2,2,4,4,6,6,8,8,10,10,12,12,14,14,16,16,18,18,20,20,22,22,24,24-Tetracosamethylcyclododecasiloxa

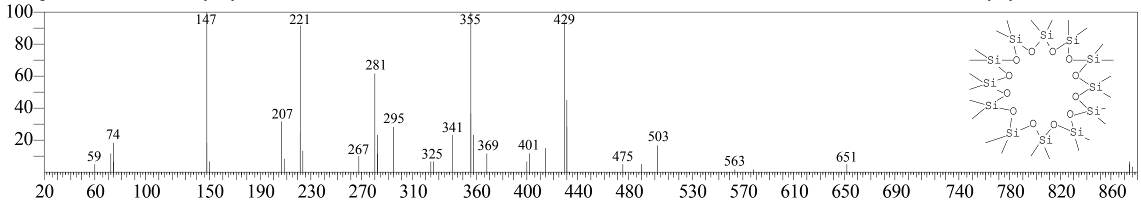

Hit#:4 Entry:241761 Library:NIST14.lib
SI:57 Formula:C20H60O10Si10 CAS:18772-36-6 MolWeight:740 RetIndex:2067
CompName:Cyclodecasiloxane, eicosamethyl- $$ 2,2,4,4,6,6,8,8,10,10,12,12,14,14,16,16,18,18,20,20-Icosamethylcyclodecasiloxane # $$ Eicosamethyl-cy

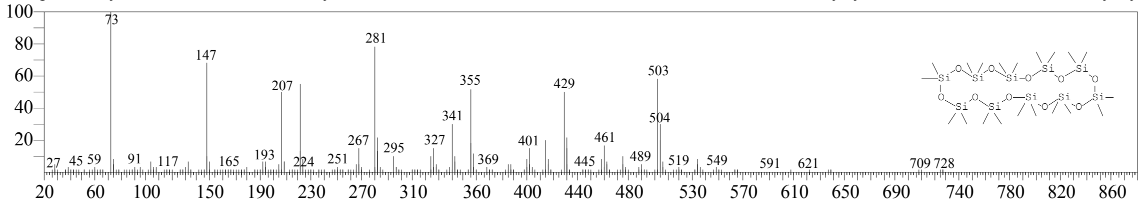

Hit#:5 Entry:236030 Library:NIST14.lib
SI:55 Formula:C16H48O6Si7 CAS:541-01-5 MolWeight:532 RetIndex:1437
CompName:Heptasiloxane, hexadecamethyl- $$ Hexadecamethylheptasiloxane $$ 1,1,1,3,3,5,5,7,7,9,9,11,11,13,13,13-Hexadecamethylheptasiloxane # $$

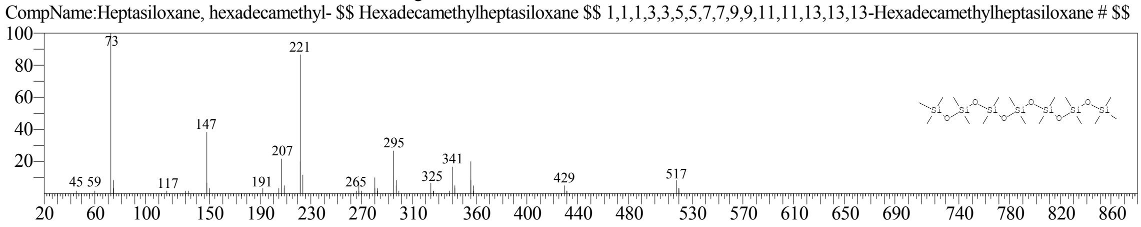

<< Target >>
Line#:12 R.Time:16.120(Scan#:3487) MassPeaks:300
RawMode:Averaged 16.117-16.123(3486-3488) BasePeak:59.05(1515)
BG Mode:Calc. from Peak Group 1 - Event 1 Scan

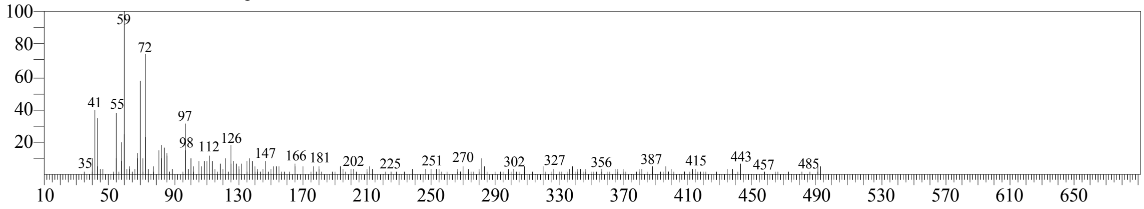

Hit#:1 Entry:164178 Library:NIST14.lib
SI:76 Formula:C22H43NO CAS:112-84-5 MolWeight:337 RetIndex:2625
CompName:13-Docosenamide, (Z)- $$ Erucylamide $$ Erucyl amide $$ (Z)-13-Docosenamide $$ 13-Docosenamide, cis- $$ Armid E $$ cis-13-Docosenan

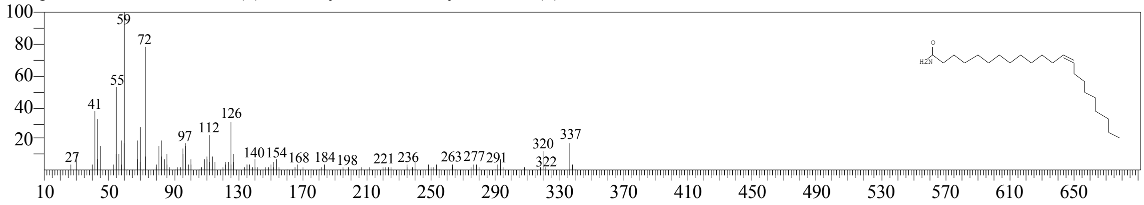

Hit#:2 Entry:241225 Library:NIST14.lib
SI:73 Formula:C45H86N2O2 CAS:10436-19-8 MolWeight:686 RetIndex:5311
CompName:Bis(cis-13-docosenamido)methane $$ (13Z)-N-([(13Z)-13-Docosenoylamino]methyl)-13-docosenamide # $$

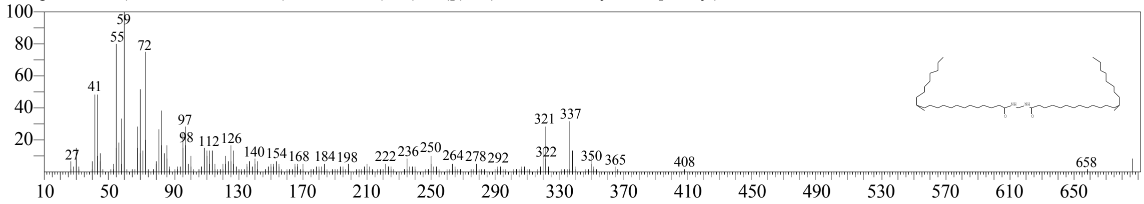

Hit#:3 Entry:139276 Library:NIST14.lib
SI:72 Formula:C20H39NO CAS:10436-08-5 MolWeight:309 RetIndex:2427
CompName:cis-11-Eicosenamide $$ (11Z)-11-Icosenamide # $$

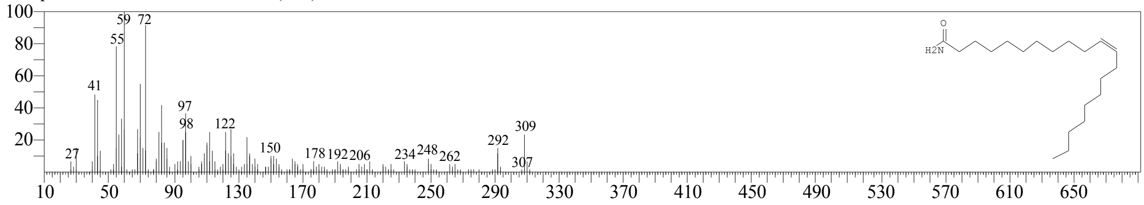

Hit#:4 Entry:139275 Library:NIST14.lib
SI:71 Formula:C20H39NO CAS:10586-57-9 MolWeight:309 RetIndex:2427
CompName:trans-11-Icosenamide $$ (11E)-11-Icosenamide # $$

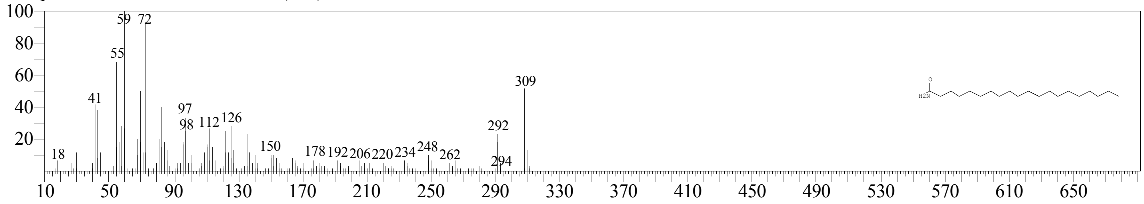

Hit#:5 Entry:128462 Library:NIST14.lib
SI:71 Formula:C19H39NO CAS:58185-32-3 MolWeight:297 RetIndex:2319
CompName:Nonadecanamide

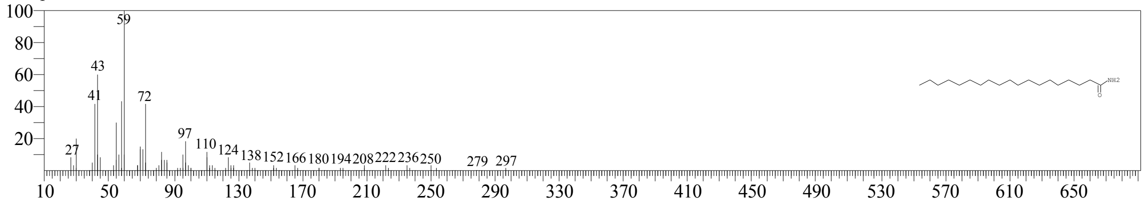

Supplement: Supplementary file 1 [file FSN3-7-2176-s001.pdf]
