# Supplementary material for: Evaluation of nutritional value of Asystasia mysorensis and Sesamum angustifolia and their potential contribution to human health
Source: Food Sci Nutr. 2019 May 15;7(6):2176–85. doi: 10.1002/fsn3.1064 (PMC6593372; doi:10.1002/fsn3.1064)

Library

<< Target >>
Line#:1 R.Time:6.227(Scan#:519) MassPeaks:256
RawMode:Averaged 6.223-6.230(518-520) BasePeak:43.05(13234)
BG Mode:Calc. from Peak Group 1 - Event 1 Scan

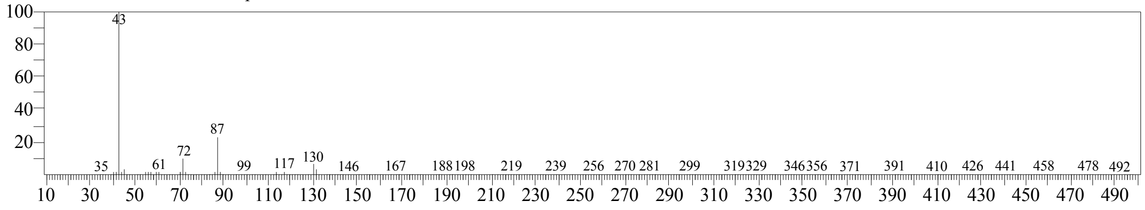

Hit#:1 Entry:29103 Library:NIST14.lib
SI:94 Formula:C8H14O4 CAS:1114-92-7 MolWeight:174 RetIndex:1023
CompName:2,3-Butanediol, diacetate $$ 2-(Acetyloxy)-1-methylpropyl acetate # $$ 2,3-Butanediyl diacetate $$ Butane-2,3-diol, diacetate $$ 2,3-Diacetoxy

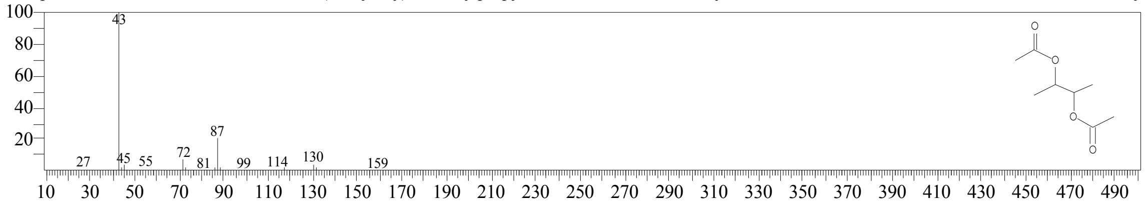

Hit#:2 Entry:7964 Library:NIST14.lib
SI:89 Formula:C6H10O3 CAS:4906-24-5 MolWeight:130 RetIndex:857
CompName:CH3C(O)OCH(CH3)C(O)CH3 $$ 2-Acetoxy-3-butanone $$

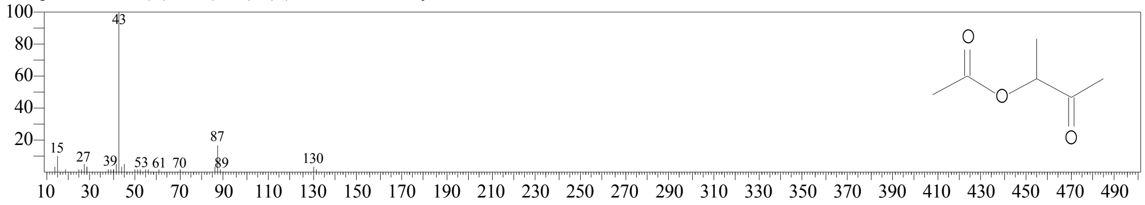

Hit#:3 Entry:7965 Library:NIST14.lib
SI:86 Formula:C6H10O3 CAS:10150-87-5 MolWeight:130 RetIndex:921
CompName:2-Butanone, 4-(acetyloxy)- $$ 2-Butanone, 4-hydroxy-, acetate $$ 1-Acetoxybutan-3-one $$ 4-Acetoxy-2-butanone $$ CH3C(O)OCH2CH2C((

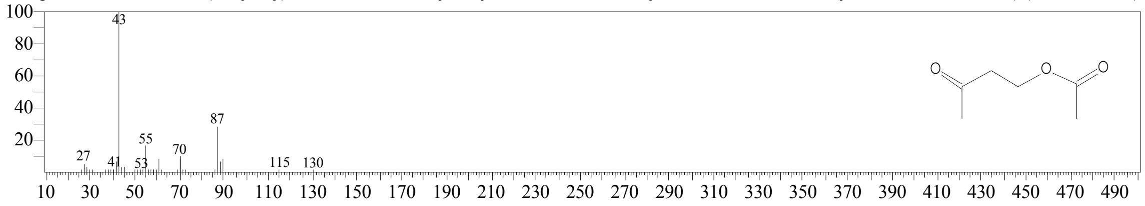

Hit#:4 Entry:15428 Library:NIST14.lib
SI:85 Formula:C6H11ClO2 CAS:54192-20-0 MolWeight:150 RetIndex:862
CompName:2-Butanol, 3-chloro-, acetate, (R*,R*)- $$ 2-Chloro-1-methylpropyl acetate # $$

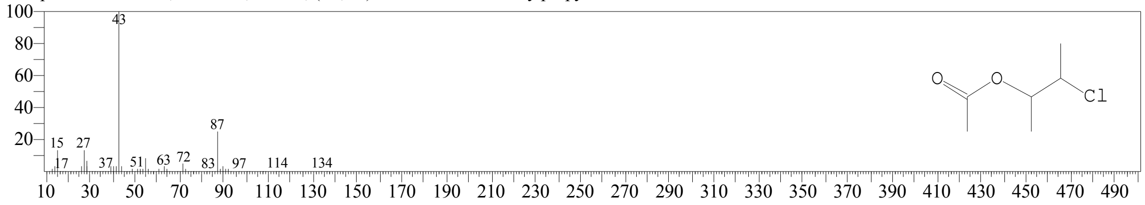

Hit#:5 Entry:39249 Library:NIST14.lib
SI:85 Formula:C8H14O5 CAS:10526-21-3 MolWeight:190 RetIndex:1099
CompName:Ethanol, 1,1'-oxybis-, diacetate $$ Ethanol, 1,1'-oxydi-, diacetate $$ di(1-Acetoxyethyl) ether $$ 1-[1-(Acetyloxy)ethoxy]ethyl acetate # $$

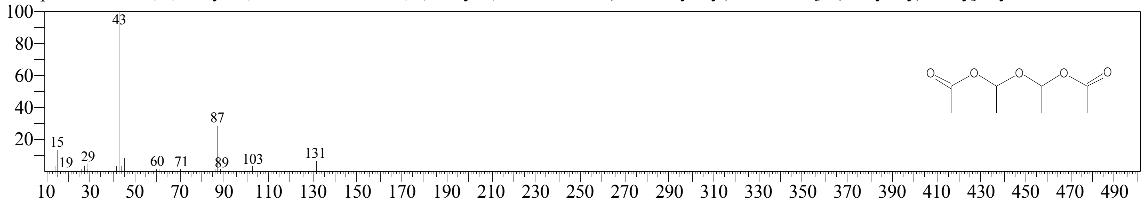

<< Target >>
Line#:2 R.Time:4.797(Scan#:90) MassPeaks:249
RawMode:Averaged 4.793-4.800(89-91) BasePeak:43.00(12123)
BG Mode:Calc. from Peak Group 1 - Event 1 Scan

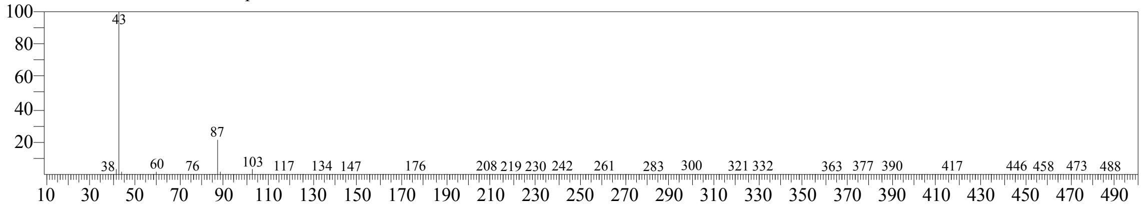

Hit#:1 Entry:13859 Library:NIST14.lib
SI:93 Formula:C6H10O4 CAS:542-10-9 MolWeight:146 RetIndex:888
CompName:1,1-Ethanediol, diacetate $$ Ethylidene acetate $$ Ethylidene diacetate $$ 1,1-Diacetoxyethane $$ 1,1'-Diacetoxy-ethane $$ 1-(Acetyloxy)ethyl

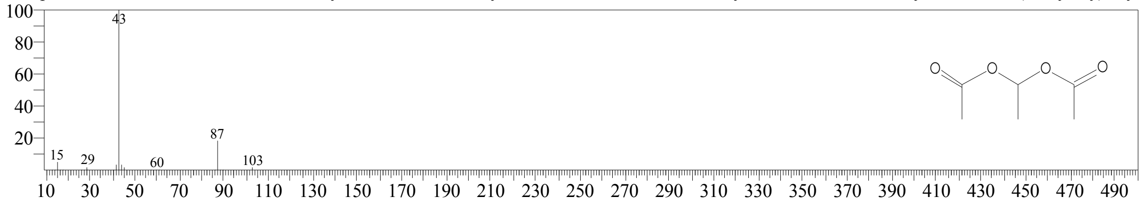

Hit#:2 Entry:39249 Library:NIST14.lib
SI:89 Formula:C8H14O5 CAS:10526-21-3 MolWeight:190 RetIndex:1099
CompName:Ethanol, 1,1'-oxybis-, diacetate $$ Ethanol, 1,1'-oxydi-, diacetate $$ di(1-Acetoxyethyl) ether $$ 1-[1-(Acetyloxy)ethoxy]ethyl acetate # $$

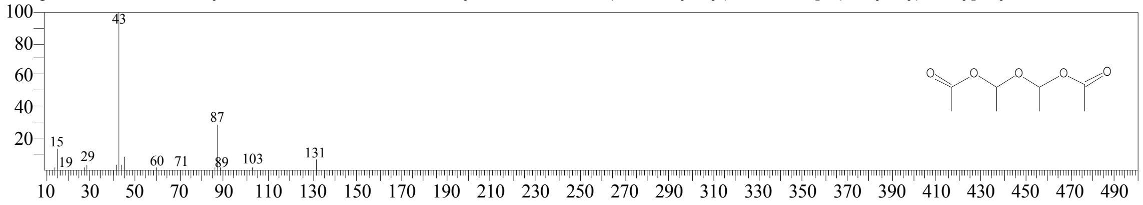

Hit#:3 Entry:7964 Library:NIST14.lib
SI:89 Formula:C6H10O3 CAS:4906-24-5 MolWeight:130 RetIndex:857
CompName:CH3C(O)OCH(CH3)C(O)CH3 $$ 2-Acetoxy-3-butanone $$

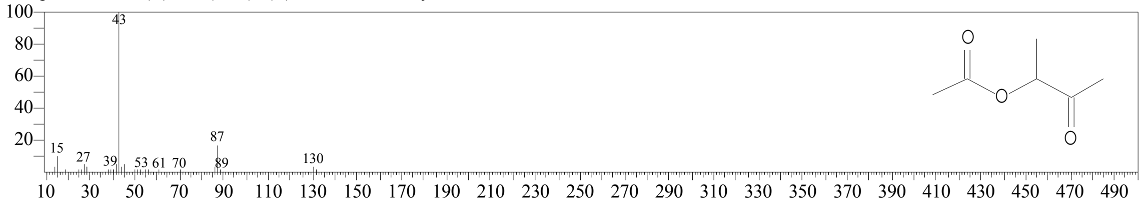

Hit#:4 Entry:2242 Library:NIST14.lib
SI:86 Formula:C4H6O3 CAS:108-24-7 MolWeight:102 RetIndex:722
CompName:Acetic anhydride $$ Acetic acid, anhydride $$ Acetic oxide $$ Acetyl anhydride $$ Acetyl ether $$ Acetyl oxide $$ Ethanoic anhydride $$ (CH

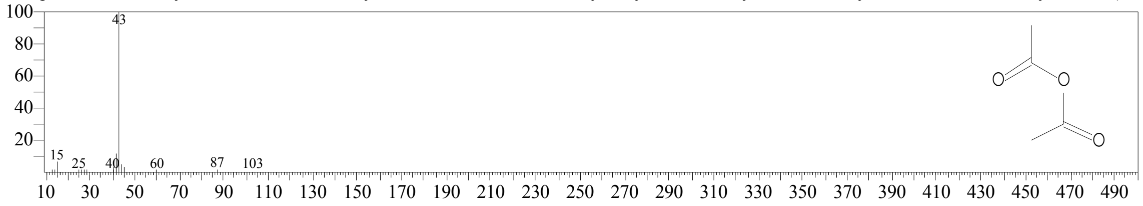

Hit#:5 Entry:813 Library:NIST14.lib
SI:86 Formula:C4H6O2 CAS:108-05-4 MolWeight:86 RetIndex:576
CompName:Acetic acid ethenyl ester $$ Acetic acid vinyl ester $$ Vinyl acetate $$ Vinyl A monomer $$ VyAc $$ 1-Acetoxyethylene $$ CH3CO2CH=CH2

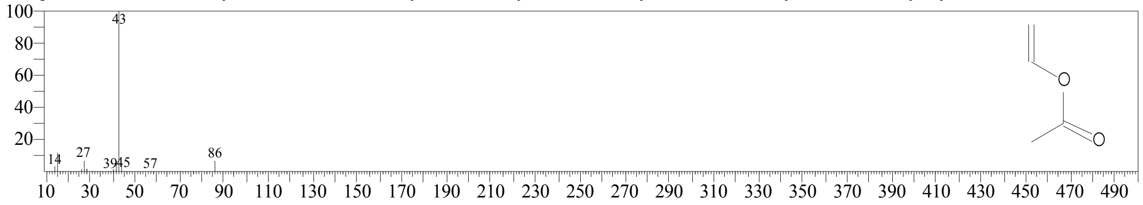

<< Target >>
Line#:3   R.Time:5.830(Scan#:400) MassPeaks:242
RawMode:Averaged 5.827-5.833(399-401) BasePeak:83.05(9292)
BG Mode:Calc. from Peak Group 1 - Event 1 Scan

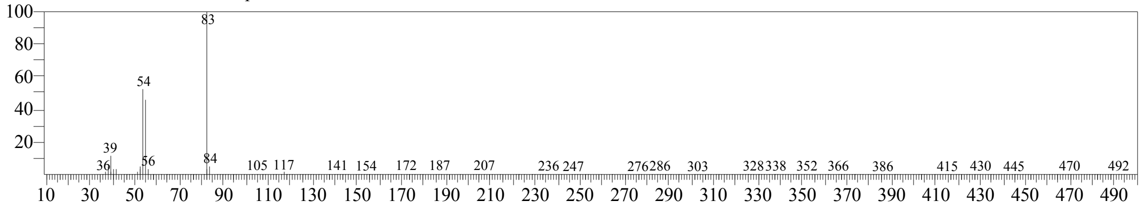

Hit#:1 Entry:17804 Library:NIST14.lib
SI:87 Formula:C9H14O2 CAS:0-00-0 MolWeight:154 RetIndex:1058
CompName:But-3-enyl (E)-2-methylbut-2-enoate

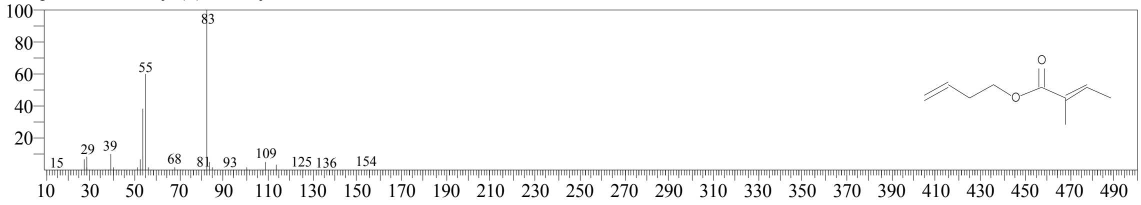

Hit#:2 Entry:15525 Library:NIST14.lib
SI:84 Formula:C8H10N2O CAS:61985-22-6 MolWeight:150 RetIndex:1234
CompName:1-Imidazol-1-yl-3-methylbut-2-en-1-one $$ 1-(3-Methyl-2-butenoyl)-1H-imidazole # $$

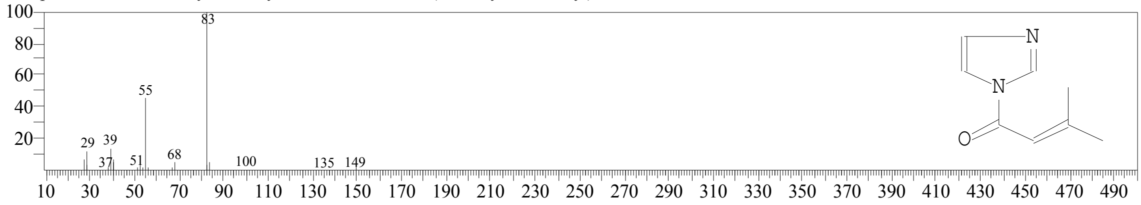

Hit#:3 Entry:4958 Library:NIST14.lib
SI:84 Formula:C5H7ClO CAS:3350-78-5 MolWeight:118 RetIndex:816
CompName:3,3-Dimethylacryloyl chloride $$ 3,3-Dimethylacrylyl chloride $$ 2-Butenoyl chloride, 3-methyl- $$ 3-Methyl-2-butenoyl chloride # $$

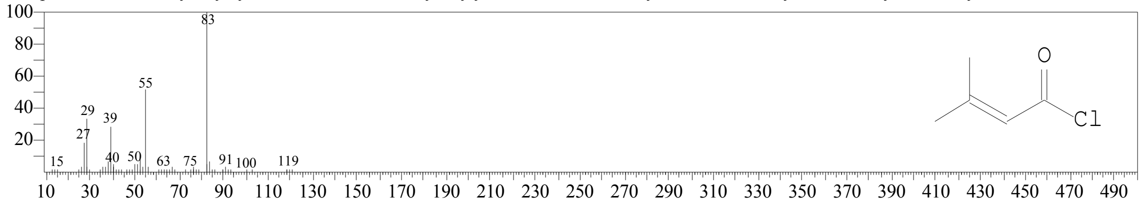

Hit#:4 Entry:16862 Library:NIST14.lib
SI:84 Formula:C10H16O CAS:546-49-6 MolWeight:152 RetIndex:1042
CompName:1,5-Heptadien-4-one, 3,3,6-trimethyl- $$ Artemisia ketone $$ Isoartemisia ketone $$ 2,5,5-Trimethyl-2,6-heptadien-4-one $$ Artemesia $$ 3,3,

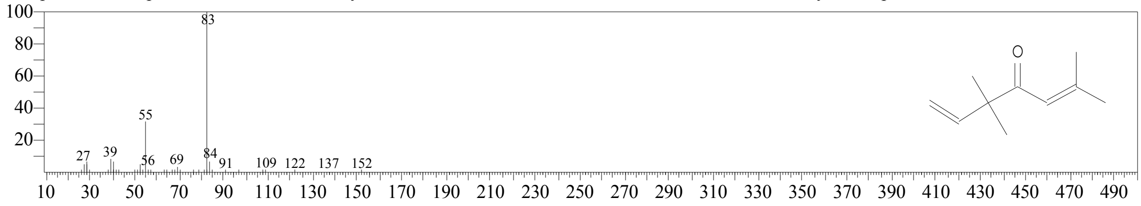

Hit#:5 Entry:21701 Library:NIST14.lib
SI:83 Formula:C6H11Br CAS:108-85-0 MolWeight:162 RetIndex:977
CompName:Cyclohexane, bromo- $$ Bromocyclohexane $$ Cyclohexyl bromide $$ 1-Bromocyclohexane $$

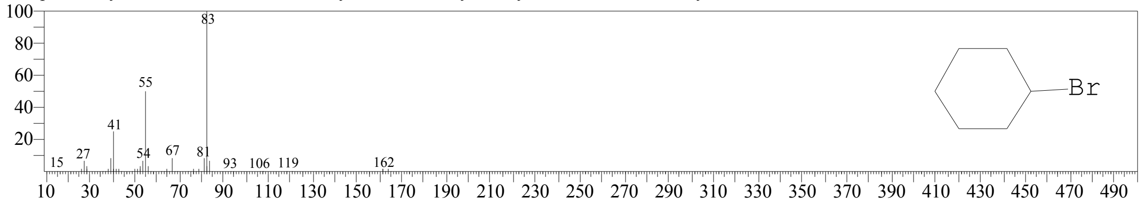

<< Target >>
Line#:4   R.Time:6.110(Scan#:484) MassPeaks:261
RawMode:Averaged 6.107-6.113(483-485) BasePeak:43.00(13356)
BG Mode:Calc. from Peak Group 1 - Event 1 Scan

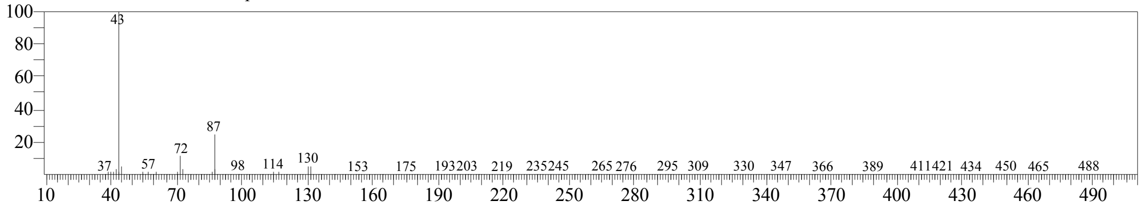

Hit#:1 Entry:29103 Library:NIST14.lib
SI:94 Formula:C8H14O4 CAS:1114-92-7 MolWeight:174 RetIndex:1023
CompName:2,3-Butanediol, diacetate $$ 2-(Acetyloxy)-1-methylpropyl acetate # $$ 2,3-Butanediyl diacetate $$ Butane-2,3-diol, diacetate $$ 2,3-Diacetoxy

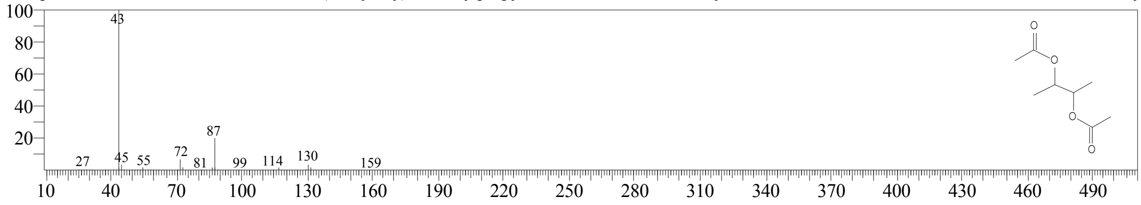

Hit#:2 Entry:7964 Library:NIST14.lib
SI:89 Formula:C6H10O3 CAS:4906-24-5 MolWeight:130 RetIndex:857
CompName:CH3C(O)OCH(CH3)C(O)CH3 $$ 2-Acetoxy-3-butanone $$

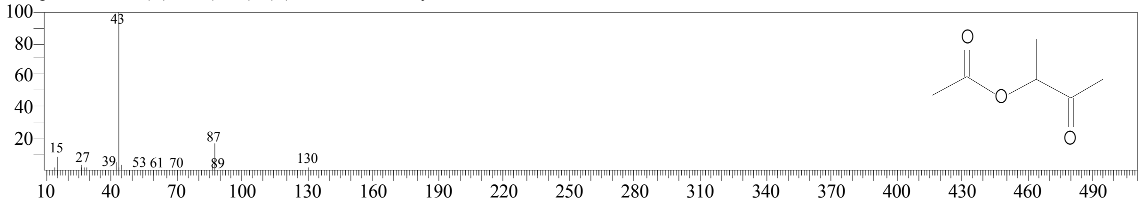

Hit#:3 Entry:15428 Library:NIST14.lib
SI:86 Formula:C6H11ClO2 CAS:54192-20-0 MolWeight:150 RetIndex:862
CompName:2-Butanol, 3-chloro-, acetate, (R*,R*)- $$ 2-Chloro-1-methylpropyl acetate # $$

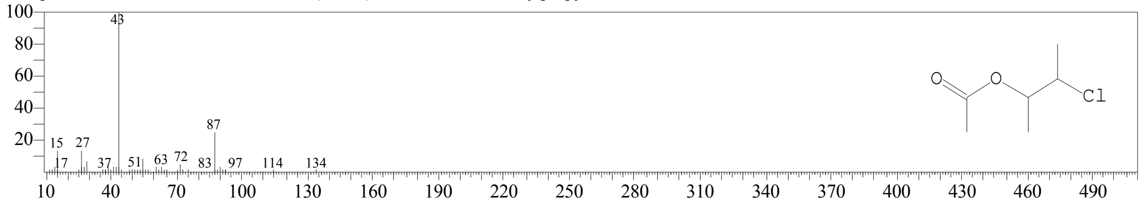

Hit#:4 Entry:7965 Library:NIST14.lib
SI:85 Formula:C6H10O3 CAS:10150-87-5 MolWeight:130 RetIndex:921
CompName:2-Butanone, 4-(acetyloxy)- $$ 2-Butanone, 4-hydroxy-, acetate $$ 1-Acetoxybutan-3-one $$ 4-Acetoxy-2-butanone $$ CH3C(O)OCH2CH2C(

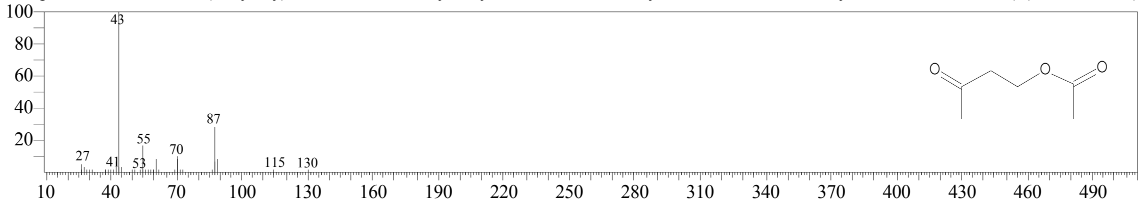

Hit#:5 Entry:39249 Library:NIST14.lib
SI:84 Formula:C8H14O5 CAS:10526-21-3 MolWeight:190 RetIndex:1099
CompName:Ethanol, 1,1'-oxybis-, diacetate $$ Ethanol, 1,1'-oxydi-, diacetate $$ di(1-Acetoxyethyl) ether $$ 1-[1-(Acetyloxy)ethoxy]ethyl acetate # $$

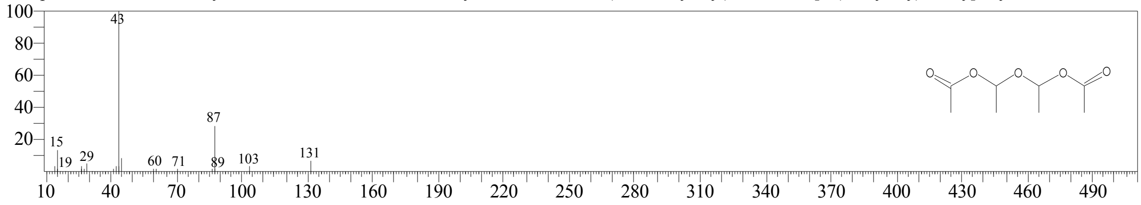

<< Target >>
Line#:5   R.Time:6.227(Scan#:519) MassPeaks:256
RawMode:Averaged 6.223-6.230(518-520) BasePeak:43.05(13234)
BG Mode:Calc. from Peak Group 1 - Event 1 Scan

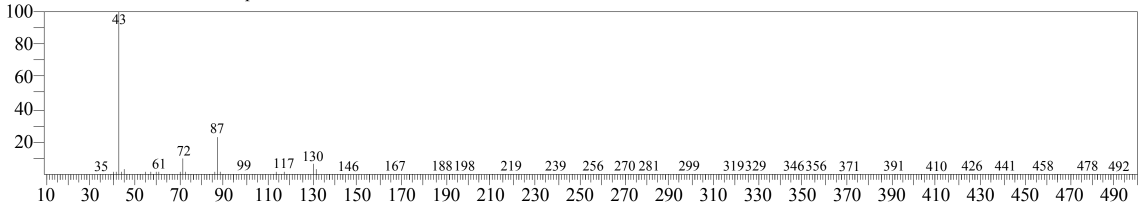

Hit#:1 Entry:29103 Library:NIST14.lib
SI:94 Formula:C8H14O4 CAS:1114-92-7 MolWeight:174 RetIndex:1023
CompName:2,3-Butanediol, diacetate $$ 2-(Acetyloxy)-1-methylpropyl acetate # $$ 2,3-Butanediyl diacetate $$ Butane-2,3-diol, diacetate $$ 2,3-Diacetoxy

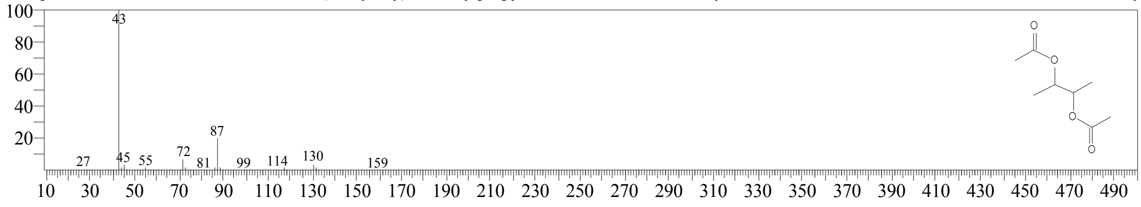

Hit#:2 Entry:7964 Library:NIST14.lib
SI:89 Formula:C6H10O3 CAS:4906-24-5 MolWeight:130 RetIndex:857
CompName:CH3C(O)OCH(CH3)C(O)CH3 $$ 2-Acetoxy-3-butanone $$

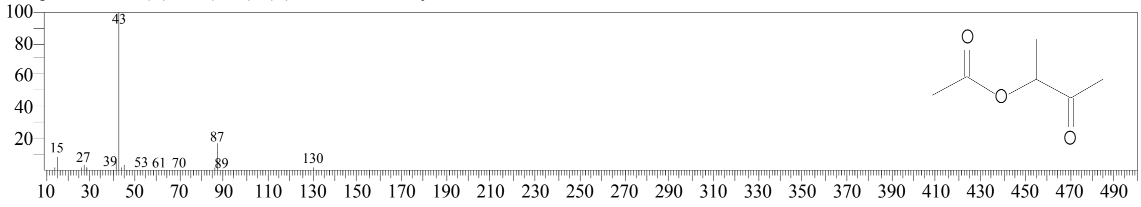

Hit#:3 Entry:7965 Library:NIST14.lib
SI:86 Formula:C6H10O3 CAS:10150-87-5 MolWeight:130 RetIndex:921
CompName:2-Butanone, 4-(acetyloxy)- $$ 2-Butanone, 4-hydroxy-, acetate $$ 1-Acetoxybutan-3-one $$ 4-Acetoxy-2-butanone $$ CH3C(O)OCH2CH2C((

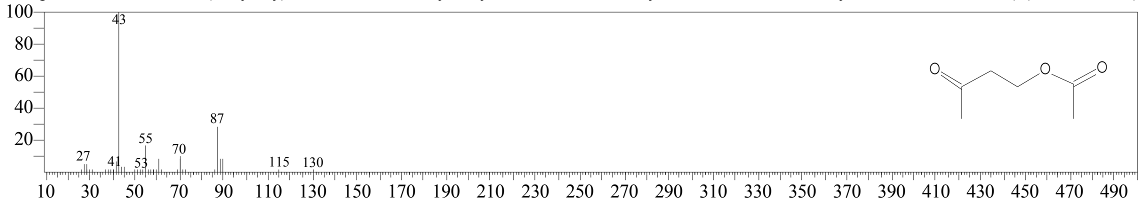

Hit#:4 Entry:15428 Library:NIST14.lib
SI:85 Formula:C6H11ClO2 CAS:54192-20-0 MolWeight:150 RetIndex:862
CompName:2-Butanol, 3-chloro-, acetate, (R*,R*)- $$ 2-Chloro-1-methylpropyl acetate # $$

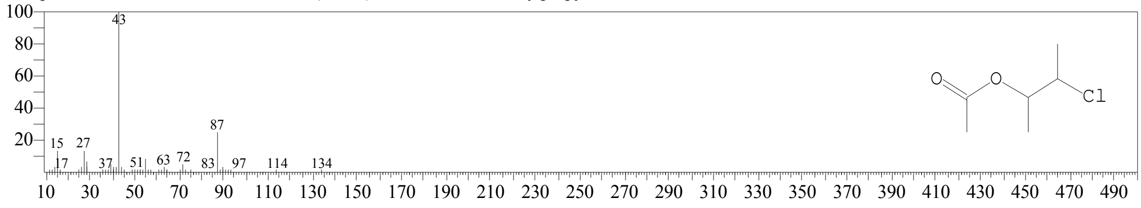

Hit#:5 Entry:39249 Library:NIST14.lib
SI:85 Formula:C8H14O5 CAS:10526-21-3 MolWeight:190 RetIndex:1099
CompName:Ethanol, 1,1'-oxybis-, diacetate $$ Ethanol, 1,1'-oxydi-, diacetate $$ di(1-Acetoxyethyl) ether $$ 1-[1-(Acetyloxy)ethoxy]ethyl acetate # $$

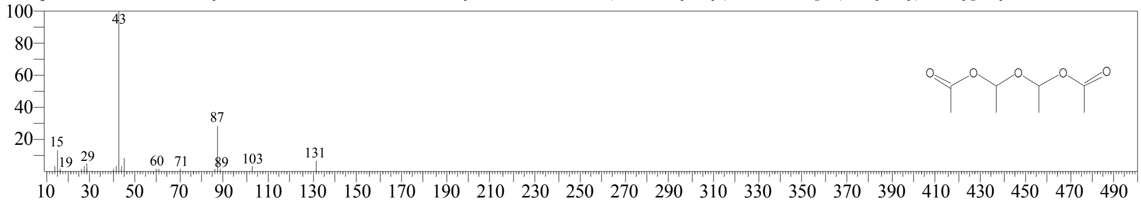

<< Target >>
Line#:6 R.Time:6.590(Scan#:628) MassPeaks:253
RawMode:Averaged 6.587-6.593(627-629) BasePeak:43.05(2114)
BG Mode:Calc. from Peak Group 1 - Event 1 Scan

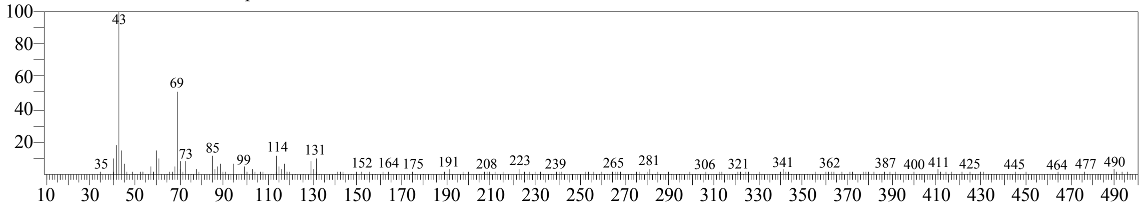

Hit#:1 Entry:29101 Library:NIST14.lib
SI:83 Formula:C8H14O4 CAS:27846-49-7 MolWeight:174 RetIndex:1087
CompName:Ethyl 3-acetoxybutyrate $$ Ethyl 3-(acetyloxy)butanoate $$ Ethyl 3-acetoxybutanoate $$ Butyric acid, 3-hydroxy-, ethyl ester, acetate $$

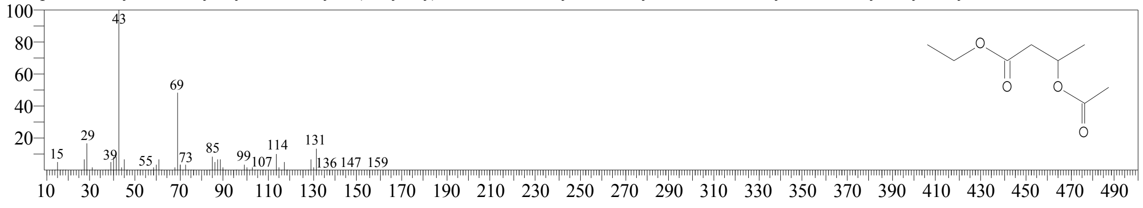

Hit#:2 Entry:50748 Library:NIST14.lib
SI:73 Formula:C8H14O6 CAS:5987-62-2 MolWeight:206 RetIndex:1549
CompName:.beta.-d-Ribopyranoside, methyl, 3-acetate $$ Methyl 3-O-acetylpentopyranoside # $$

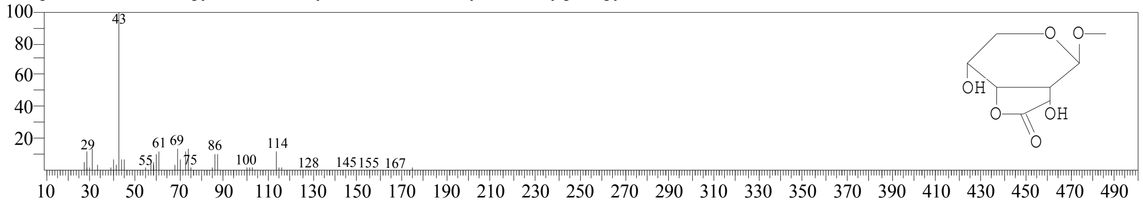

Hit#:3 Entry:27942 Library:NIST14.lib
SI:73 Formula:C8H12O4 CAS:10476-95-6 MolWeight:172 RetIndex:1054
CompName:2-Propene-1,1-diol, 2-methyl-, diacetate $$ Methacrolein diacetate $$ Methallylidene diacetate $$ Acetic acid, 2-methyl-2-propene-1,1-diol dies

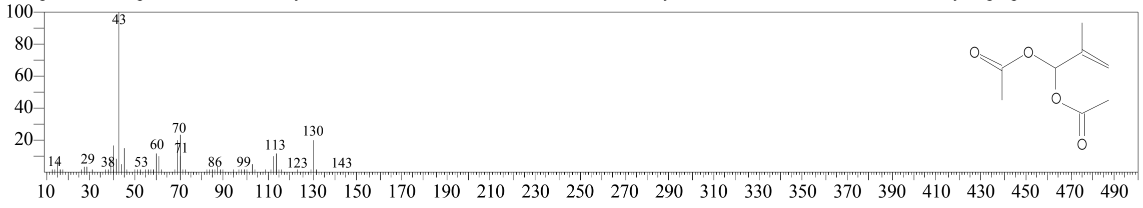

Hit#:4 Entry:13856 Library:NIST14.lib
SI:71 Formula:C6H10O4 CAS:0-00-0 MolWeight:146 RetIndex:1077
CompName:(+-)-3-hydroxybutyric acid, acetate

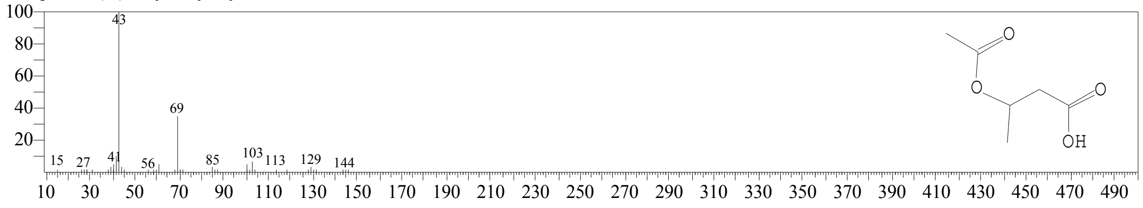

Hit#:5 Entry:14674 Library:NIST14.lib
SI:71 Formula:C6H12O4 CAS:0-00-0 MolWeight:148 RetIndex:1062
CompName:3-Methoxymethoxybutyric acid $$ 3-(Methoxymethoxy)butanoic acid # $$

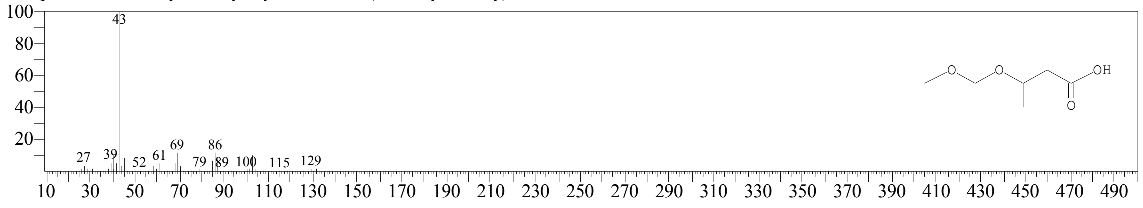

<< Target >>
Line#:7 R.Time:6.650(Scan#:646) MassPeaks:251
RawMode:Averaged 6.647-6.653(645-647) BasePeak:83.05(4053)
BG Mode:Calc. from Peak Group 1 - Event 1 Scan

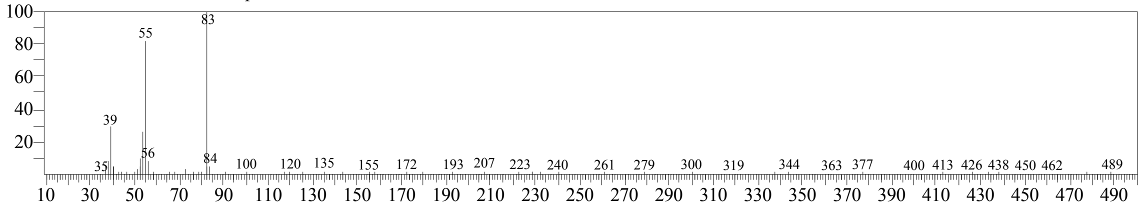

Hit#:1 Entry:4958 Library:NIST14.lib
SI:83 Formula:C5H7ClO CAS:3350-78-5 MolWeight:118 RetIndex:816
CompName:3,3-Dimethylacryloyl chloride $$ 3,3-Dimethylacrylyl chloride $$ 2-Butenoyl chloride, 3-methyl- $$ 3-Methyl-2-butenoyl chloride # $$

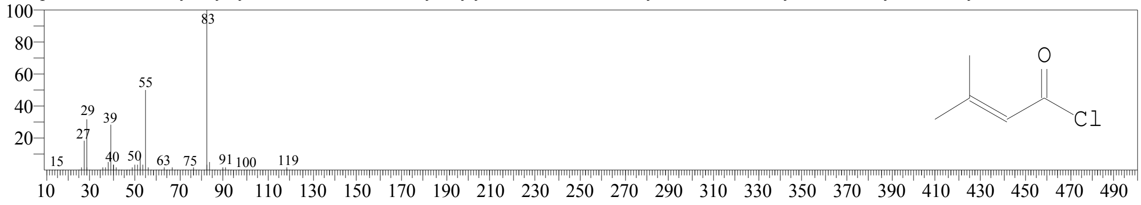

Hit#:2 Entry:17751 Library:NIST14.lib
SI:83 Formula:C9H14O2 CAS:61692-78-2 MolWeight:154 RetIndex:0
CompName:2-Butenoic acid, 2-methyl-, 2-methyl-2-propenyl ester, (Z)-

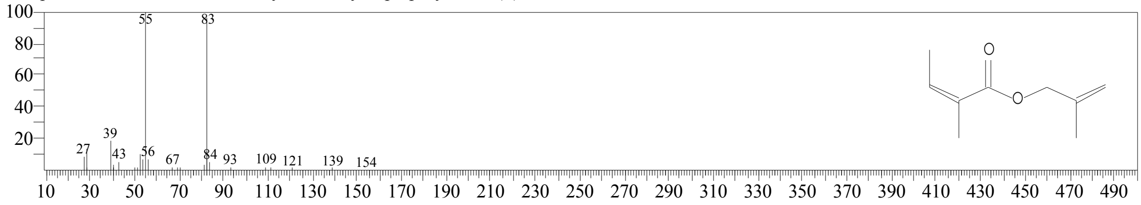

Hit#:3 Entry:17802 Library:NIST14.lib
SI:82 Formula:C9H14O2 CAS:61692-82-8 MolWeight:154 RetIndex:0
CompName:2-Butenoic acid, 2-methyl-, 2-methyl-2-propenyl ester, (E)-

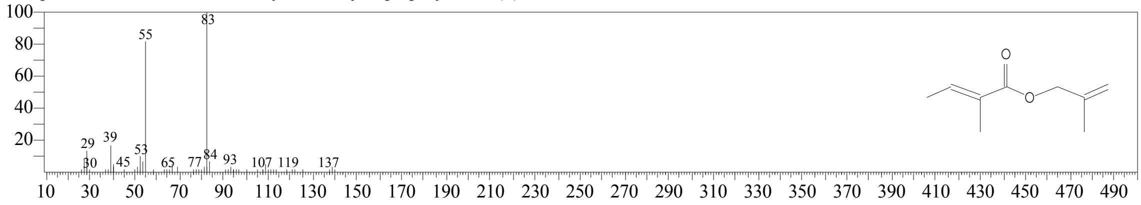

Hit#:4 Entry:17804 Library:NIST14.lib
SI:81 Formula:C9H14O2 CAS:0-00-0 MolWeight:154 RetIndex:1058
CompName:But-3-enyl (E)-2-methylbut-2-enoate

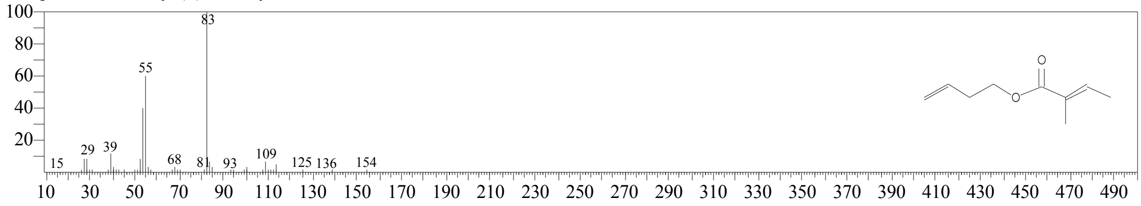

Hit#:5 Entry:6283 Library:NIST14.lib
SI:81 Formula:C6H11N3 CAS:19573-22-9 MolWeight:125 RetIndex:0
CompName:Cyclohexane, azido- $$ Cyclohexyl azide $$ Azidocyclohexane $$

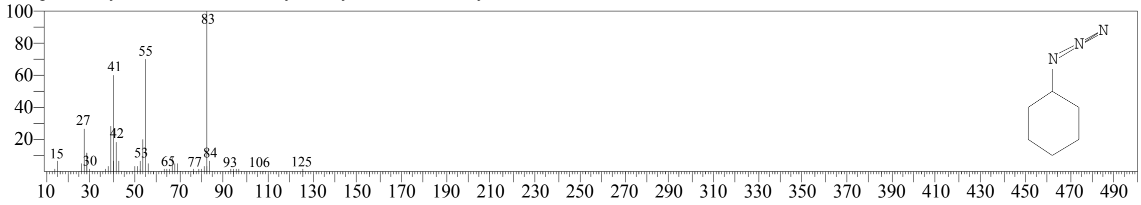

<< Target >>
Line#:8   R.Time:6.723(Scan#:668)   MassPeaks:272
RawMode:Averaged 6.720-6.727(667-669) BasePeak:43.00(17998)
BG Mode:Calc. from Peak   Group 1 - Event 1   Scan

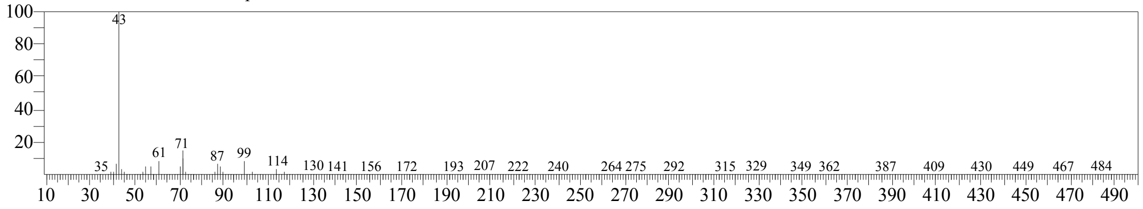

Hit#:1   Entry:29102   Library:NIST14.lib
SI:96   Formula:C8H14O4   CAS:1117-31-3   MolWeight:174   RetIndex:1087
CompName:1,3-Butanediol, diacetate $$ 1,3-Butylene diacetate $$ 1,3-Butylene glycol diacetate $$ 1,3-Diacetoxybutane $$ 1,3-Butandiol diacetate $$ 3-(A

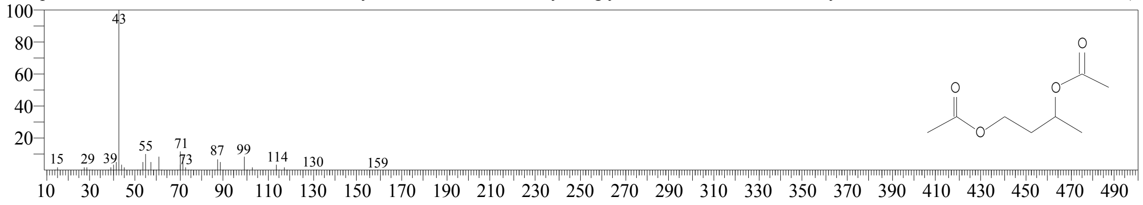

Hit#:2   Entry:29099   Library:NIST14.lib
SI:87   Formula:C8H14O4   CAS:628-67-1   MolWeight:174   RetIndex:1151
CompName:1,4-Butanediol, diacetate $$ Butylene glycol diacetate $$ Tetramethylene acetate $$ Tetramethylene diacetate $$ 1,4-Butylene glycol diacetate $

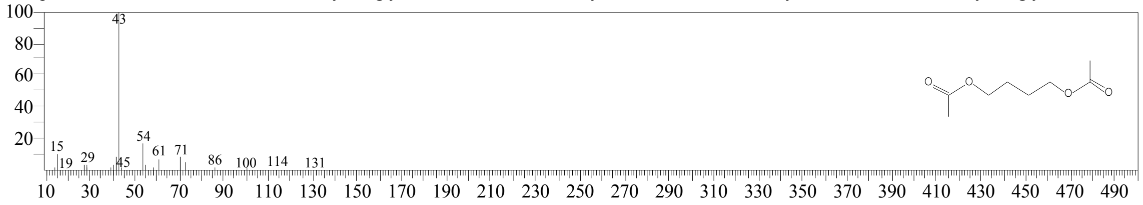

Hit#:3   Entry:20717   Library:NIST14.lib
SI:86   Formula:C7H12O4   CAS:628-66-0   MolWeight:160   RetIndex:1052
CompName:1,3-Propanediol, diacetate $$ Trimethylene acetate $$ 1,3-Diacetoxypropane $$ 1,3-Propylene diacetate $$ 1,3-Propylene glycol diacetate $$ 3-

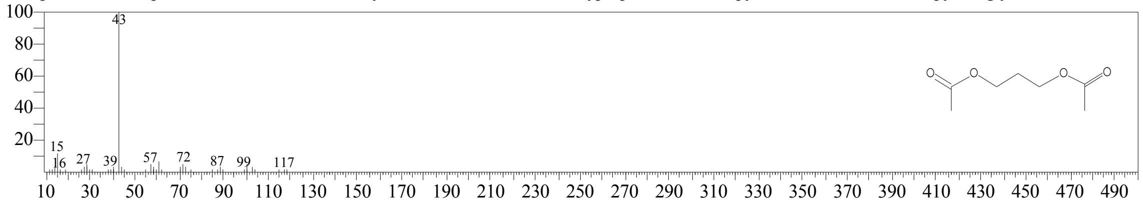

Hit#:4   Entry:29103   Library:NIST14.lib
SI:86   Formula:C8H14O4   CAS:1114-92-7   MolWeight:174   RetIndex:1023
CompName:2,3-Butanediol, diacetate $$ 2-(Acetyloxy)-1-methylpropyl acetate # $$ 2,3-Butanediyl diacetate $$ Butane-2,3-diol, diacetate $$ 2,3-Diacetoxy

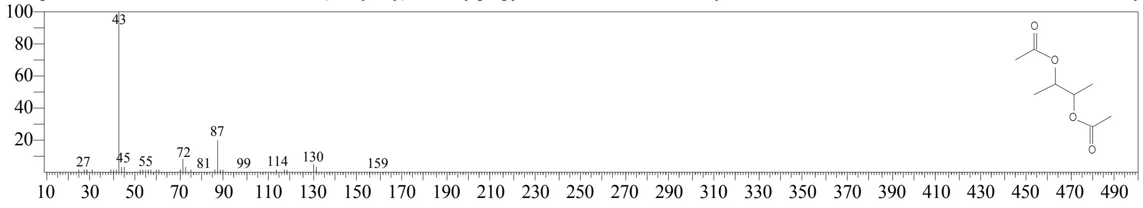

Hit#:5   Entry:4062   Library:NIST14.lib
SI:85   Formula:C6H10O2   CAS:110-13-4   MolWeight:114   RetIndex:890
CompName:2,5-Hexanedione $$ .alpha.,.beta.-Diacetylethane $$ Acetonyl acetone $$ Diacetonyl $$ 1,2-Diacetylethane $$ 2,5-Hexadione $$ Hexane-2,5-d

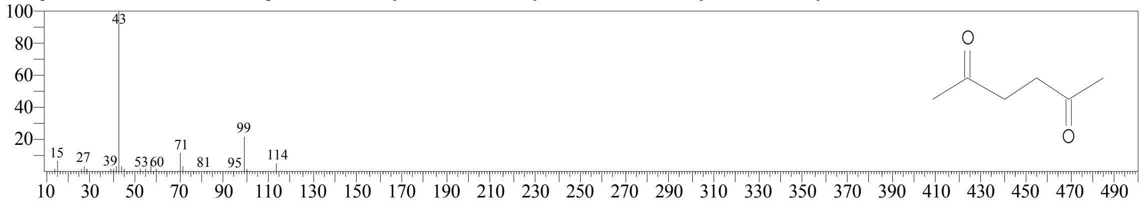

<< Target >>
Line#:9 R.Time:7.030(Scan#:760) MassPeaks:261
RawMode:Averaged 7.027-7.033(759-761) BasePeak:43.05(8965)
BG Mode:Calc. from Peak Group 1 - Event 1 Scan

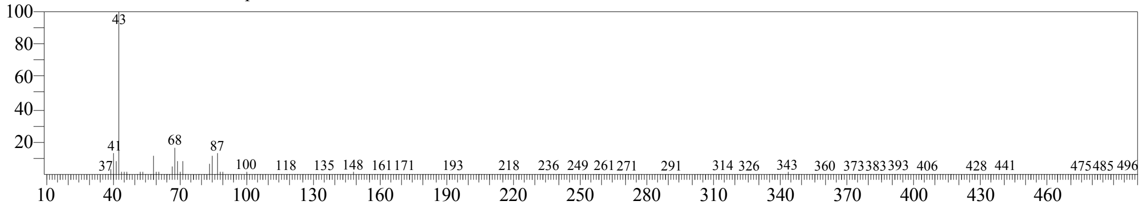

Hit#:1 Entry:72563 Library:NIST14.lib
SI:84 Formula:C9H15NO6 CAS:78651-66-8 MolWeight:233 RetIndex:1440
CompName:2,4-Pentanediol, 3-nitro-, diacetate $$ 2,4-Di-O-acetyl-1,3,5-trideoxy-3-nitropentitol # $$

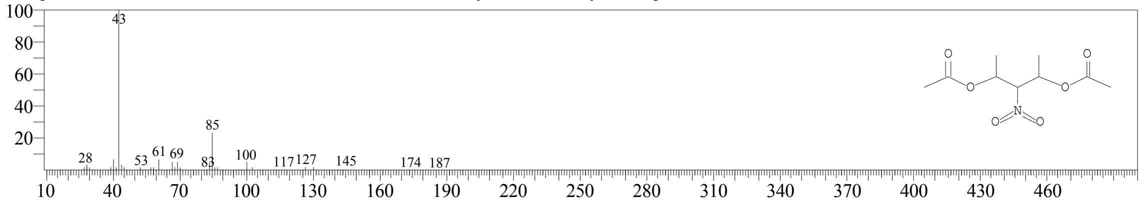

Hit#:2 Entry:7275 Library:NIST14.lib
SI:84 Formula:C7H12O2 CAS:0-00-0 MolWeight:128 RetIndex:810
CompName:4-Penten-2-ol, acetate

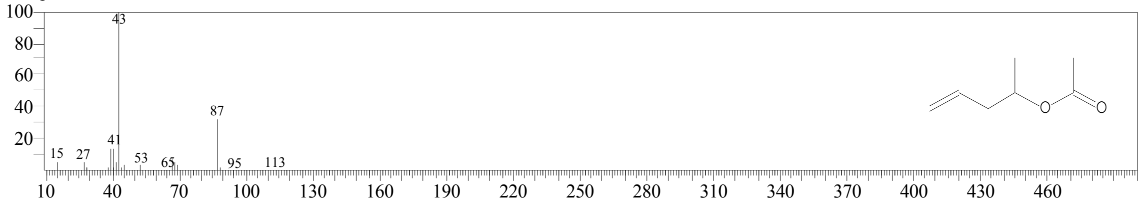

Hit#:3 Entry:862 Library:NIST14.lib
SI:83 Formula:C5H10O CAS:107-87-9 MolWeight:86 RetIndex:654
CompName:2-Pentanone $$ Ethyl acetone $$ Methyl n-propyl ketone $$ Methyl propyl ketone $$ Propyl methyl ketone $$ n-C3H7COCH3 $$ Pentan-2-on

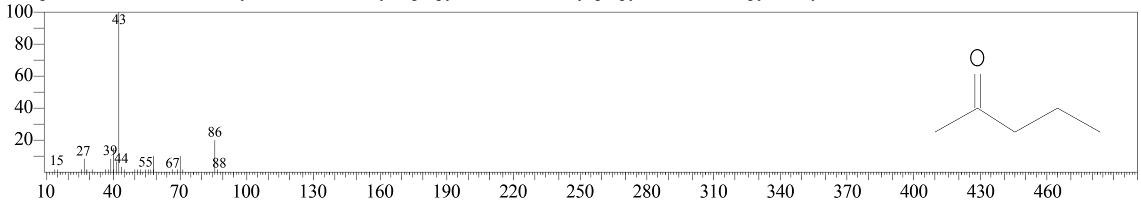

Hit#:4 Entry:14674 Library:NIST14.lib
SI:82 Formula:C6H12O4 CAS:0-00-0 MolWeight:148 RetIndex:1062
CompName:3-Methoxymethoxybutyric acid $$ 3-(Methoxymethoxy)butanoic acid # $$

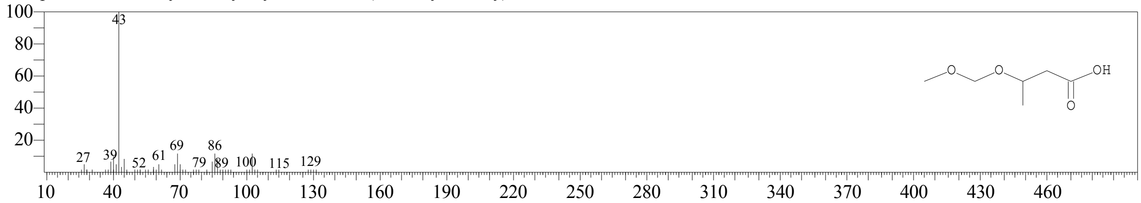

Hit#:5 Entry:2272 Library:NIST14.lib
SI:82 Formula:C5H10O2 CAS:4161-60-8 MolWeight:102 RetIndex:817
CompName:2-Pentanone, 4-hydroxy- $$ CH3CH(OH)CH2C(O)CH3 $$ 4-Hydroxy-2-pentanone # $$ 4-Hydroxy-4-methyl-2-butanone $$

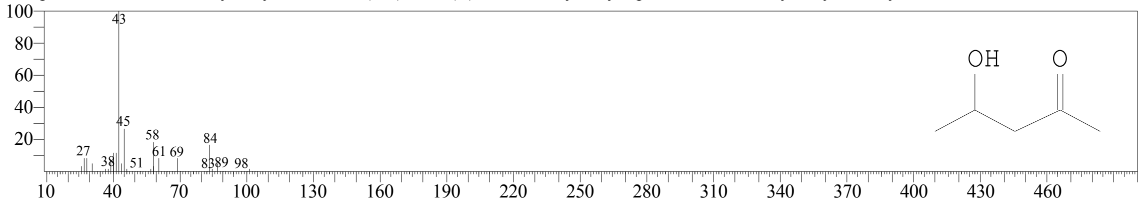

<< Target >>
Line#:10 R.Time:7.293(Scan#:839) MassPeaks:244
RawMode:Averaged 7.290-7.297(838-840) BasePeak:43.05(4931)
BG Mode:Calc. from Peak Group 1 - Event 1 Scan

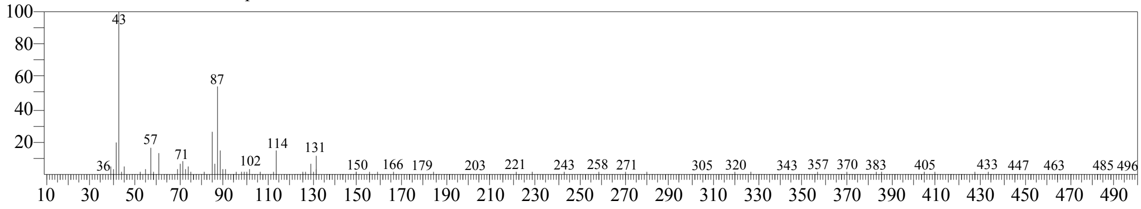

Hit#:1 Entry:29105 Library:NIST14.lib
SI:82 Formula:C8H14O4 CAS:25560-91-2 MolWeight:174 RetIndex:0
CompName:Ethyl 4-acetoxybutanoate $$ Butanoic acid, 4-(acetyloxy)-, ethyl ester $$ Butyric acid, 4-hydroxy-, ethyl ester, acetate $$ 4-Acetoxybutyric acid

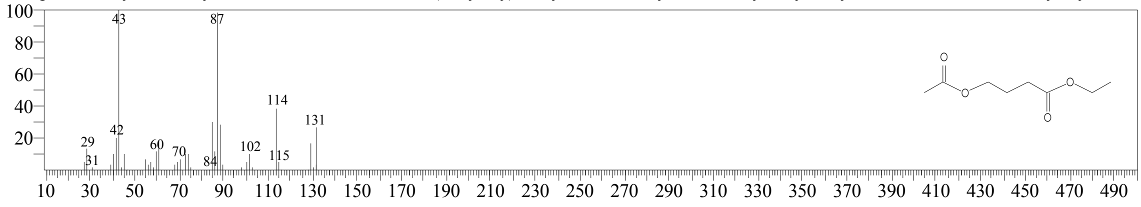

Hit#:2 Entry:29101 Library:NIST14.lib
SI:77 Formula:C8H14O4 CAS:27846-49-7 MolWeight:174 RetIndex:1087
CompName:Ethyl 3-acetoxybutyrate $$ Ethyl 3-(acetyloxy)butanoate $$ Ethyl 3-acetoxybutanoate $$ Butyric acid, 3-hydroxy-, ethyl ester, acetate $$

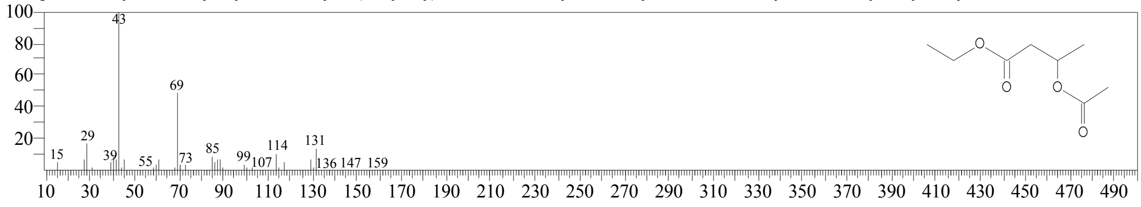

Hit#:3 Entry:13166 Library:NIST14.lib
SI:77 Formula:C7H12O3 CAS:14255-36-8 MolWeight:144 RetIndex:1047
CompName:4,4-(Ethylenedioxy)-2-pentanone $$ 1-(2-Methyl-1,3-dioxolan-2-yl)acetone # $$

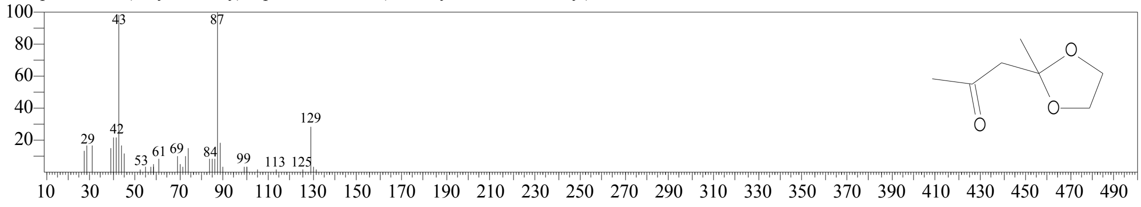

Hit#:4 Entry:29247 Library:NIST14.lib
SI:75 Formula:C9H18O3 CAS:204652-53-9 MolWeight:174 RetIndex:1095
CompName:Isopentyloxyethyl acetate $$ 2-(Isopentyloxy)ethyl acetate # $$ 2-Isopentoxyethyl acetate $$

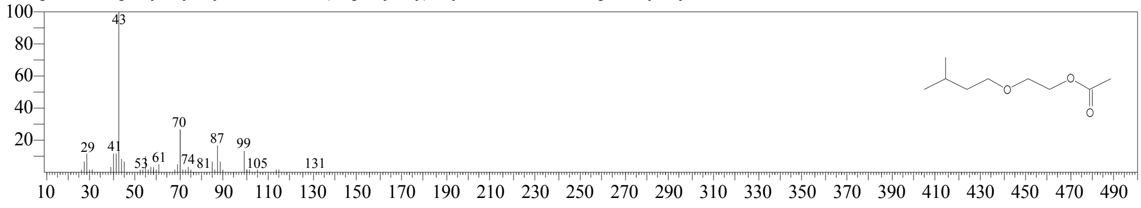

Hit#:5 Entry:29106 Library:NIST14.lib
SI:74 Formula:C8H14O4 CAS:28123-56-0 MolWeight:174 RetIndex:1272
CompName:2,5-Hexanedione, 3,4-dihydroxy-3,4-dimethyl- $$ 1,6-Dideoxy-3,4-di-c-methylhexo-2,5-diulose # $$

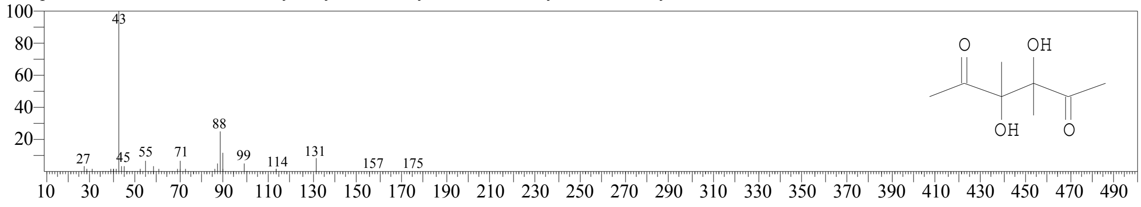

<< Target >>
Line#:11 R.Time:7.320(Scan#:847) MassPeaks:244
RawMode:Averaged 7.317-7.323(846-848) BasePeak:84.05(5959)
BG Mode:Calc. from Peak Group 1 - Event 1 Scan

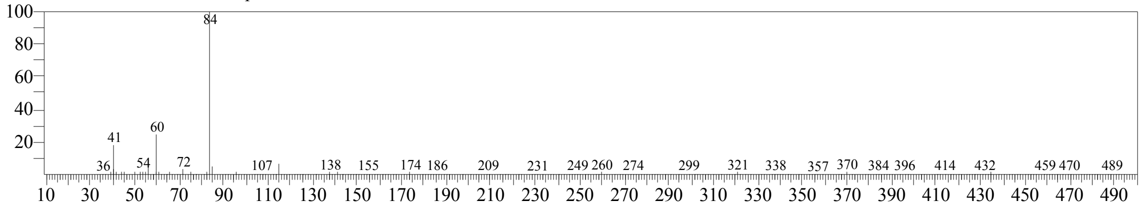

Hit#:1 Entry:4371 Library:NIST14.lib
SI:85 Formula:C5H9NO2 CAS:62400-75-3 MolWeight:115 RetIndex:1067
CompName:2-Pyrrolidinone, 5-(hydroxymethyl)- $$ Pyrrolid-2-one-5-methanol $$ 5-(Hydroxymethyl)-2-pyrrolidinone # $$

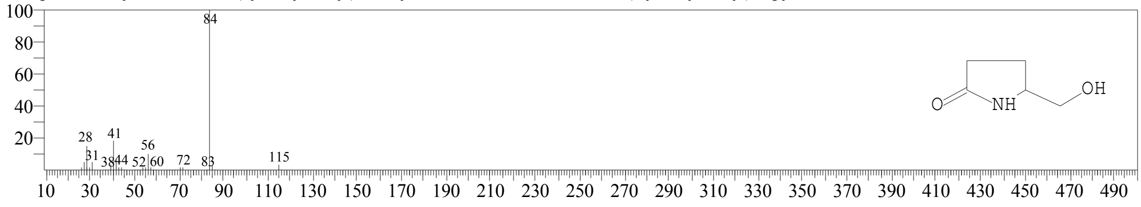

Hit#:2 Entry:12722 Library:NIST14.lib
SI:82 Formula:C6H9NO3 CAS:54571-66-3 MolWeight:143 RetIndex:1091
CompName:DL-Proline, 5-oxo-, methyl ester $$ Methyl 5-oxo-2-pyrrolidinecarboxylate # $$ 2-Pyrrolidone-5-carboxylic acid, methyl ester $$

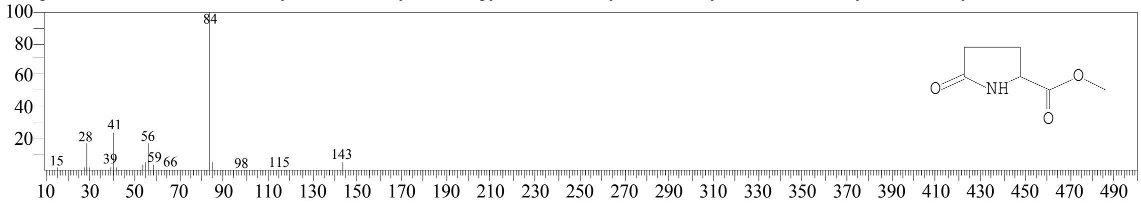

Hit#:3 Entry:12721 Library:NIST14.lib
SI:82 Formula:C6H9NO3 CAS:4931-66-2 MolWeight:143 RetIndex:1091
CompName:L-Proline, 5-oxo-, methyl ester $$ Proline, 5-oxo-, methyl ester $$ (S)-5-(Methoxycarbonyl)-2-pyrrolidone $$ Methyl pyroglutamate $$ Methyl

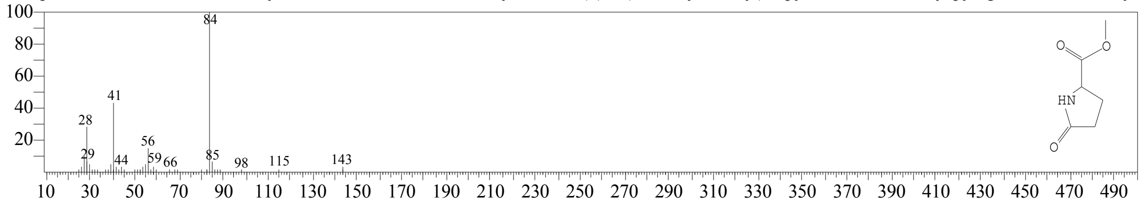

Hit#:4 Entry:4370 Library:NIST14.lib
SI:82 Formula:C5H9NO2 CAS:63853-74-7 MolWeight:115 RetIndex:900
CompName:5-Methoxypyrrolidin-2-one $$ 5-Methoxy-2-pyrrolidinone # $$

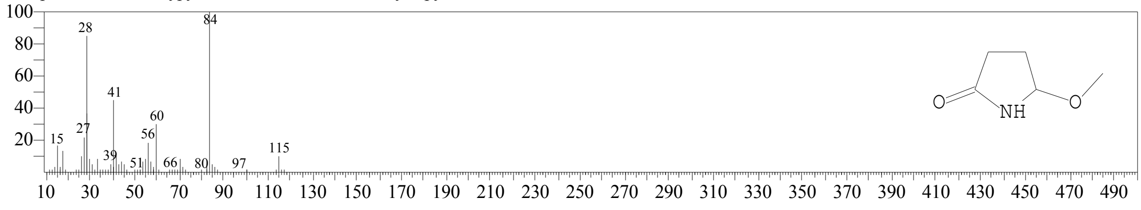

Hit#:5 Entry:7659 Library:NIST14.lib
SI:81 Formula:C5H7NO3 CAS:149-87-1 MolWeight:129 RetIndex:1180
CompName:DL-Proline, 5-oxo- $$ DL-Pyroglutamic acid $$ 5-Oxoproline # $$ 2-Pyrrolidone-5-carboxylic acid $$ DL-5-Oxoproline $$

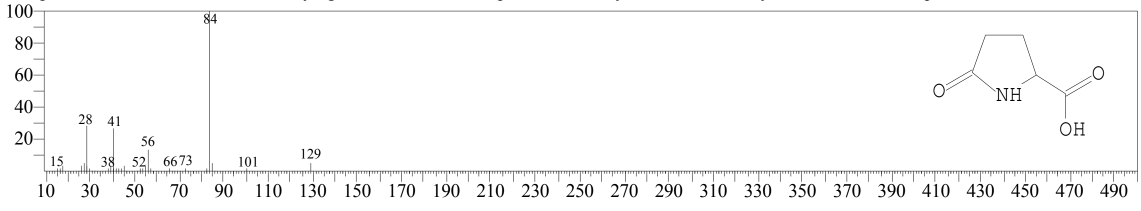

<< Target >>
Line#:12   R.Time:8.157(Scan#:1098) MassPeaks:249
RawMode:Averaged 8.153-8.160(1097-1099) BasePeak:43.00(4187)
BG Mode:Calc. from Peak Group 1 - Event 1 Scan

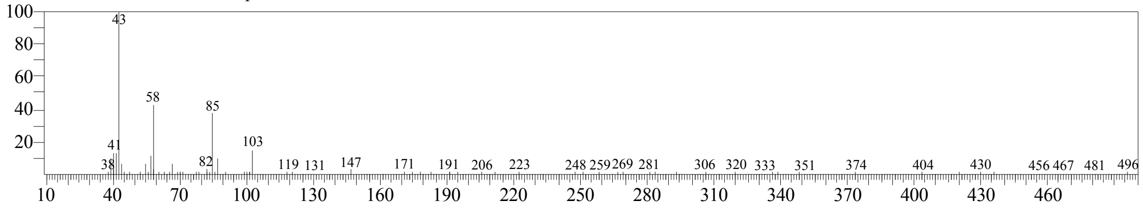

Hit#:1 Entry:7434 Library:NIST14.lib
SI:82 Formula:C8H16O CAS:6137-06-0 MolWeight:128 RetIndex:888
CompName:2-Heptanone, 4-methyl- $$ 4-Methyl-2-heptanone $$

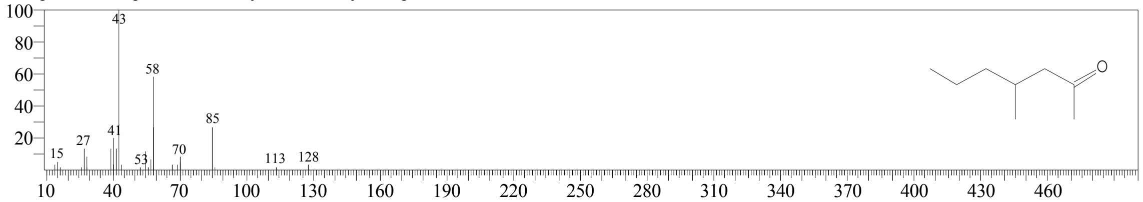

Hit#:2 Entry:28090 Library:NIST14.lib
SI:81 Formula:C9H16O3 CAS:6830-12-2 MolWeight:172 RetIndex:1155
CompName:1-Methylbutyl acetoacetate $$ 1-Methylbutyl 3-oxobutanoate # $$

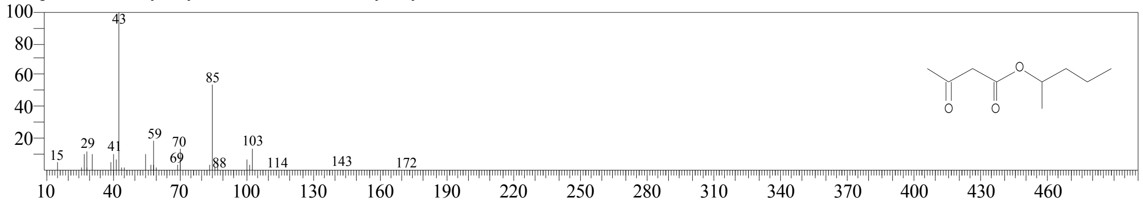

Hit#:3 Entry:36970 Library:NIST14.lib
SI:81 Formula:C10H18O3 CAS:0-00-0 MolWeight:186 RetIndex:0
CompName:Carbonic acid, hexyl prop-1-en-2-yl ester

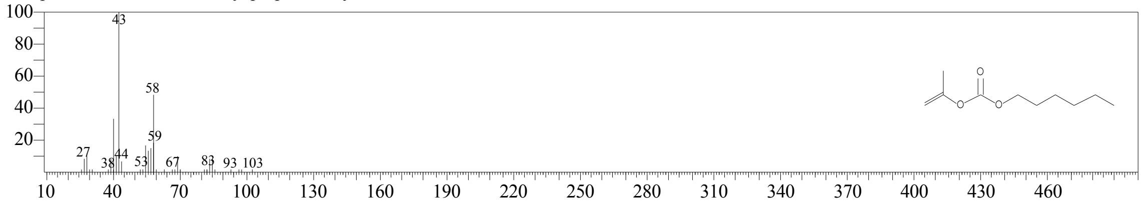

Hit#:4 Entry:12204 Library:NIST14.lib
SI:81 Formula:C7H10O3 CAS:1118-84-9 MolWeight:142 RetIndex:1010
CompName:Butanoic acid, 3-oxo-, 2-propenyl ester $$ Acetoacetic acid, allyl ester $$ Allyl acetoacetate $$ Allyl acetylacetate $$ Allyl 3-oxobutanoate # $$

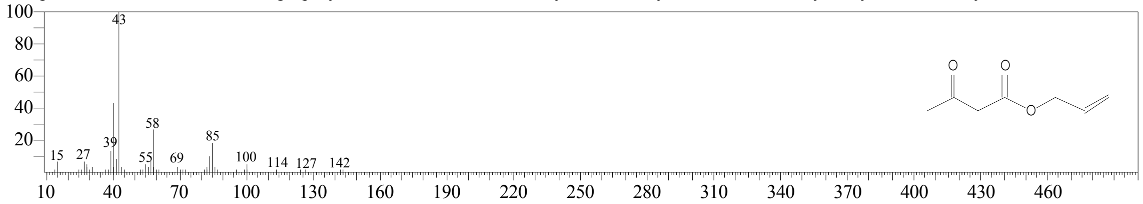

Hit#:5 Entry:20010 Library:NIST14.lib
SI:81 Formula:C9H18O2 CAS:62338-17-4 MolWeight:158 RetIndex:1150
CompName:2-Hexanone, 4-hydroxy-3-propyl- $$ 4-Hydroxy-3-propyl-2-hexanone # $$

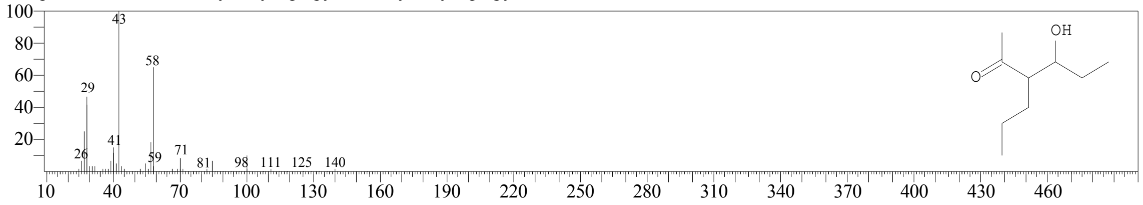

<< Target >>
Line#:13 R.Time:10.253(Scan#:1727) MassPeaks:246
RawMode:Averaged 10.250-10.257(1726-1728) BasePeak:135.10(9153)
BG Mode:Calc. from Peak Group 1 - Event 1 Scan

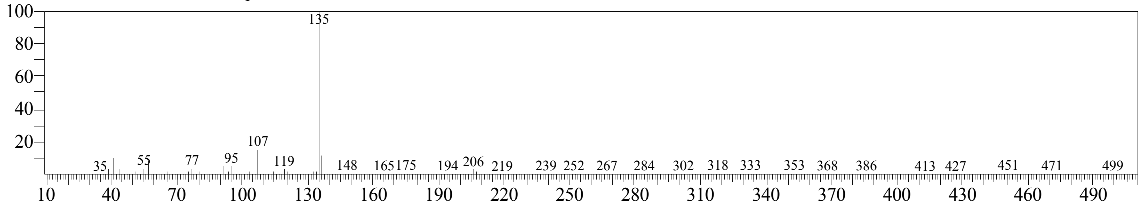

Hit#:1 Entry:51510 Library:NIST14.lib
SI:91 Formula:C14H22O CAS:0-00-0 MolWeight:206 RetIndex:0
CompName:phenol, 2-(1,1,3,3-tetramethylbutyl)- $$ 2-(2,4,4-trimethylpentan-2-yl)phenol $$

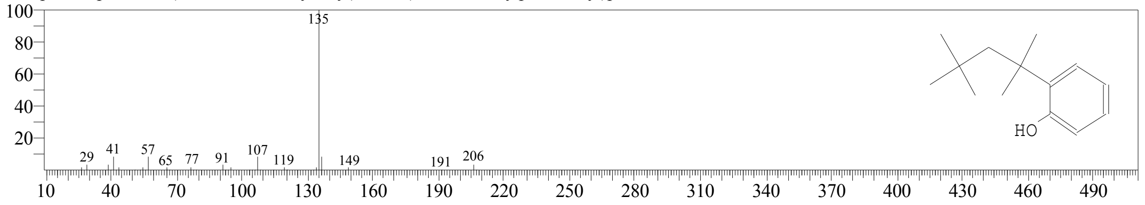

Hit#:2 Entry:51512 Library:NIST14.lib
SI:90 Formula:C14H22O CAS:140-66-9 MolWeight:206 RetIndex:1541
CompName:Phenol, 4-(1,1,3,3-tetramethylbutyl)- $$ Phenol, p-(1,1,3,3-tetramethylbutyl)- $$ p-(1,1,3,3-Tetramethylbutyl)phenol $$ p-tert-Octylphenol $$ 4

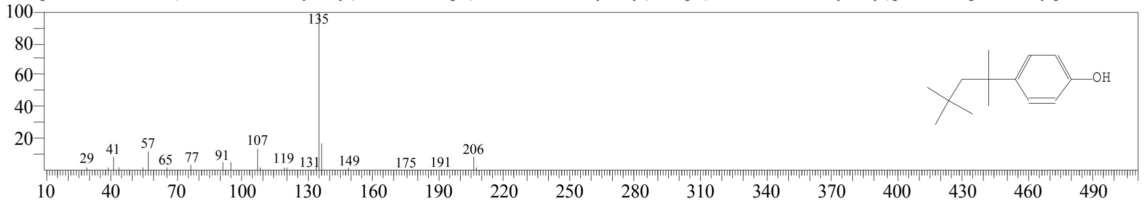

Hit#:3 Entry:211630 Library:NIST14.lib
SI:86 Formula:C19H25F6NO2 CAS:296242-69-8 MolWeight:413 RetIndex:1781
CompName:Carbamic acid, N-[1,1-bis(trifluoromethyl)ethyl]-, 4-(1,1,3,3-tetramethylbutyl)phenyl ester $$ 4-(1,1,3,3-Tetramethylbutyl)phenyl 2,2,2-trifluoro

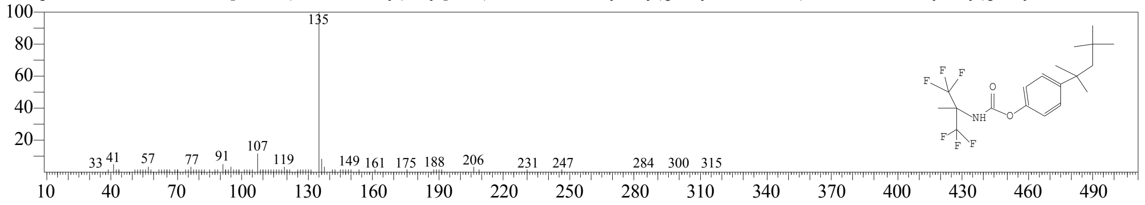

Hit#:4 Entry:23320 Library:NIST14.lib
SI:84 Formula:C11H16O CAS:80-46-6 MolWeight:164 RetIndex:1327
CompName:Phenol, 4-(1,1-dimethylpropyl)- $$ Phenol, p-tert-pentyl- $$ p-(.alpha.,.alpha.-Dimethylpropyl)phenol $$ p-tert-Amylphenol $$ p-tert-Pentylphe

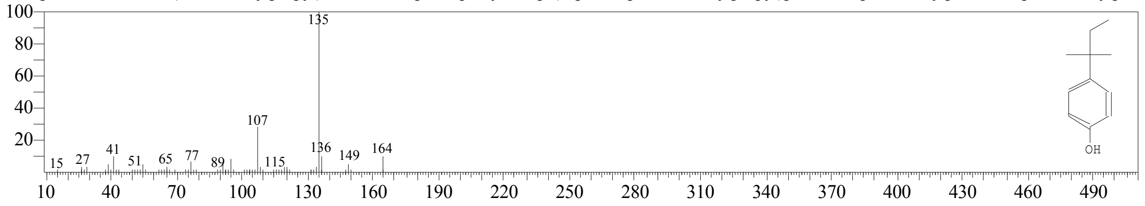

Hit#:5 Entry:104701 Library:NIST14.lib
SI:84 Formula:C18H22O2 CAS:5635-50-7 MolWeight:270 RetIndex:2277
CompName:Hexestrol $$ Phenol, 4,4'-(1,2-diethyl-1,2-ethanediyl)bis- $$ Phenol, 4,4'-(1,2-diethylethylene)di- $$ Dihydrodiethylstilbestrol $$ Hexane, 3,4-b

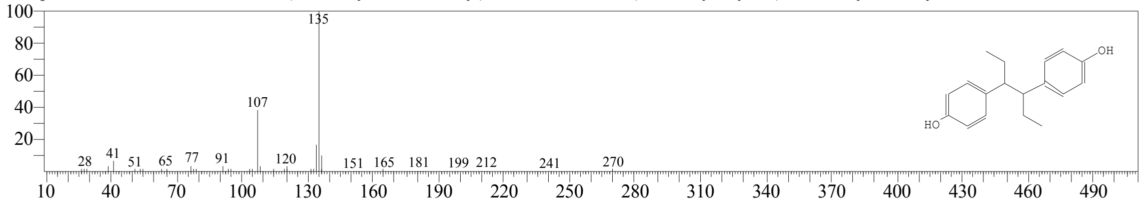

<< Target >>
Line#:14 R.Time:11.710(Scan#:2164) MassPeaks:287
RawMode:Averaged 11.707-11.713(2163-2165) BasePeak:179.10(14172)
BG Mode:Calc. from Peak Group 1 - Event 1 Scan

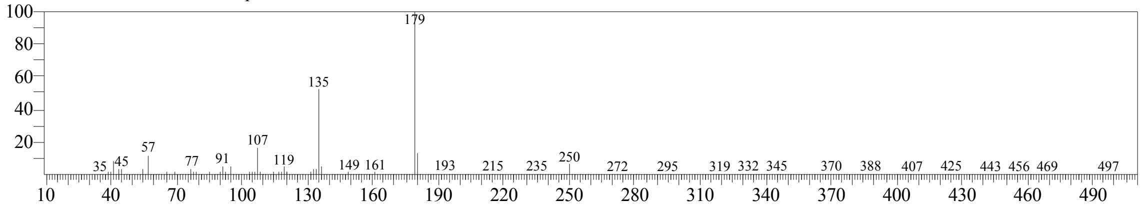

Hit#:1 Entry:42734 Library:NIST14.lib
SI:87 Formula:C12H18O2 CAS:713-46-2 MolWeight:194 RetIndex:1539
CompName:Ethanol, 2-[4-(1,1-dimethylethyl)phenoxy]- $$ Ethanol, 2-(p-tert-butylphenoxy)- $$ 2-(p-tert-Butylphenoxy)ethanol $$ 2-(p-tert-Butylphenyloxy

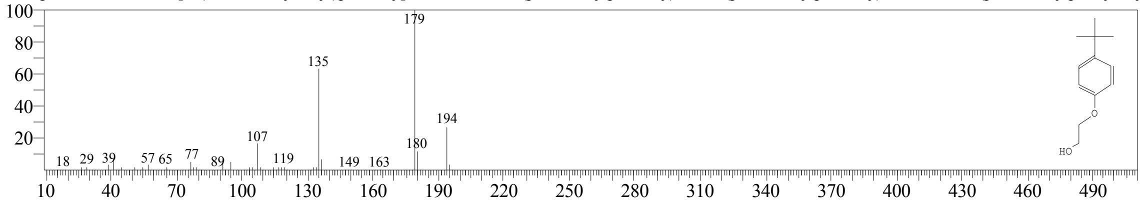

Hit#:2 Entry:53163 Library:NIST14.lib
SI:81 Formula:C13H20O2 CAS:6382-07-6 MolWeight:208 RetIndex:1638
CompName:Ethanol, 2-[4-(1,1-dimethylpropyl)phenoxy]- $$ Ethanol, 2-(p-tert-pentylphenoxy)- $$ 2-(p-tert-Amylphenoxy)ethanol $$ 2-(p-tert-Pentylpheno

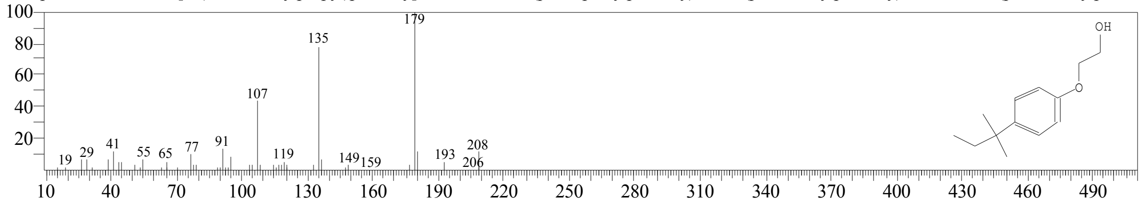

Hit#:3 Entry:42511 Library:NIST14.lib
SI:77 Formula:C11H14O3 CAS:36881-00-2 MolWeight:194 RetIndex:1475
CompName:1,3-Dioxolane, 2-(4-methoxyphenyl)-2-methyl- $$ Methyl 4-(2-methyl-1,3-dioxolan-2-yl)phenyl ether # $$

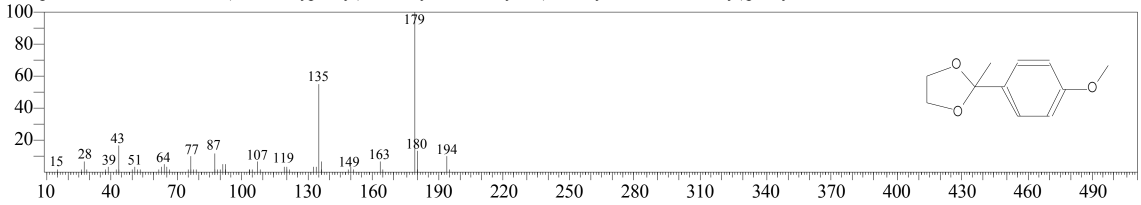

Hit#:4 Entry:143696 Library:NIST14.lib
SI:76 Formula:C18H18O5 CAS:170241-39-1 MolWeight:314 RetIndex:2668
CompName:Ethanone, 1-(5-ethyl-2-hydroxy-4-methoxyphenyl)-2-(3,4-methylenedioxyphenyl)- $$ 2-(1,3-Benzodioxol-5-yl)-1-(5-ethyl-2-hydroxy-4-methox

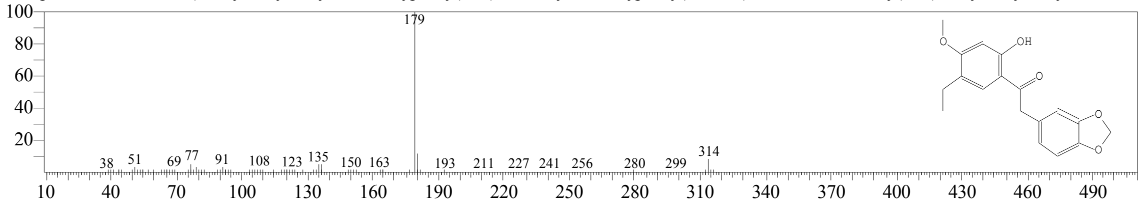

Hit#:5 Entry:52972 Library:NIST14.lib
SI:76 Formula:C12H16O3 CAS:77942-13-3 MolWeight:208 RetIndex:1620
CompName:2',4'-Dimethoxy-3'-methylpropiophenone $$ 1-(2,4-Dimethoxy-3-methylphenyl)-1-propanone # $$

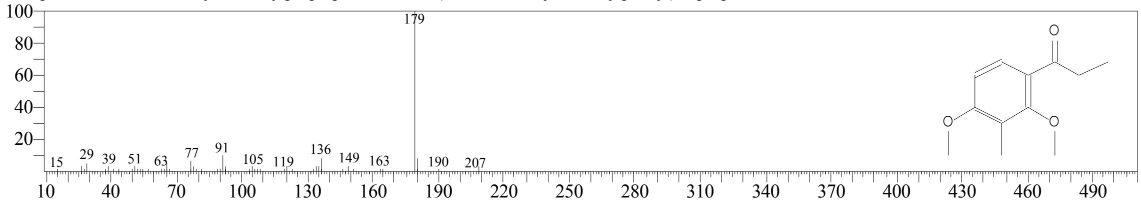

<< Target >>
Line#:15 R.Time:13.140(Scan#:2593) MassPeaks:255
RawMode:Averaged 13.137-13.143(2592-2594) BasePeak:223.15(7962)
BG Mode:Calc. from Peak Group 1 - Event 1 Scan

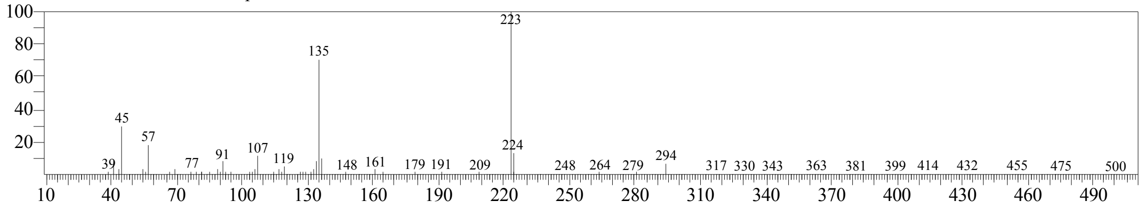

Hit#:1 Entry:125831 Library:NIST14.lib
SI:88 Formula:C18H30O3 CAS:2315-61-9 MolWeight:294 RetIndex:2126
CompName:Ethanol, 2-[2-[4-(1,1,3,3-tetramethylbutyl)phenoxy]ethoxy]- $$ 2-(2-[4-(1,1,3,3-Tetramethylbutyl)phenoxy]ethoxy)ethanol # $$

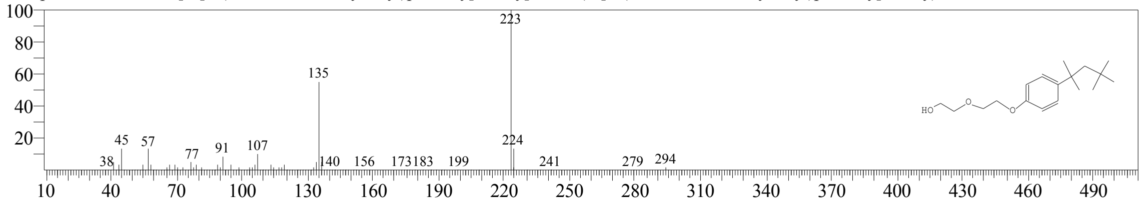

Hit#:2 Entry:77065 Library:NIST14.lib
SI:67 Formula:C14H22O3 CAS:3934-77-8 MolWeight:238 RetIndex:1996
CompName:4,6-di-t-Butylpyrogallol $$ 4,6-di-tert-Butylpyrogallol $$ 4,6-Ditert-butyl-1,2,3-benzenetriol # $$

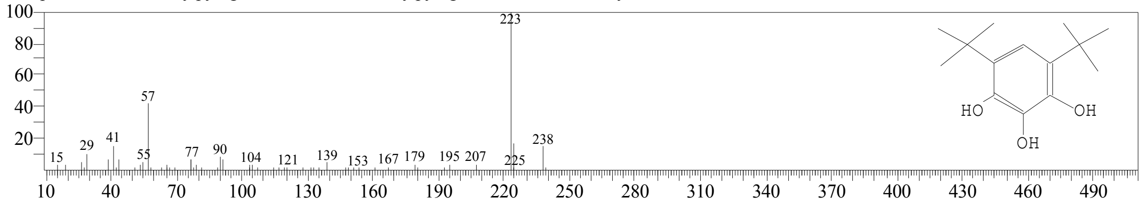

Hit#:3 Entry:219569 Library:NIST14.lib
SI:67 Formula:C23H32O8 CAS:0-00-0 MolWeight:436 RetIndex:2812
CompName:2,9-Dioxabicyclo[3.3.1]non-7-ene-8-methanol, 6-acetoxy-1,4-dimethyl-3-(1,3-dimethyl-6-methoxy-6-oxo-2,4-hexadienyl)-, acetate

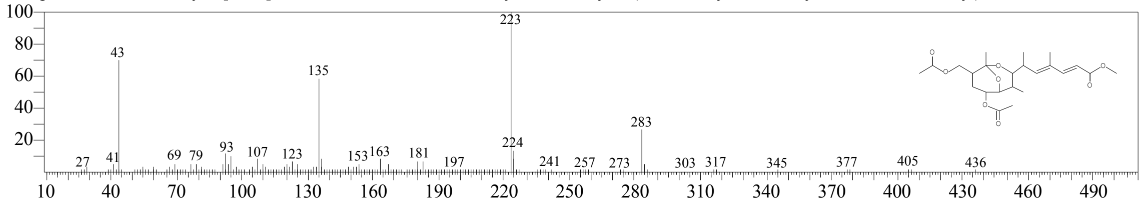

Hit#:4 Entry:99156 Library:NIST14.lib
SI:65 Formula:C16H24O3 CAS:30504-23-5 MolWeight:264 RetIndex:1690
CompName:Butanoic acid, 2-(1-adamantyl)-3-oxo-, ethyl ester $$ Ethyl 2-(1-adamantyl)-3-oxobutanoate # $$

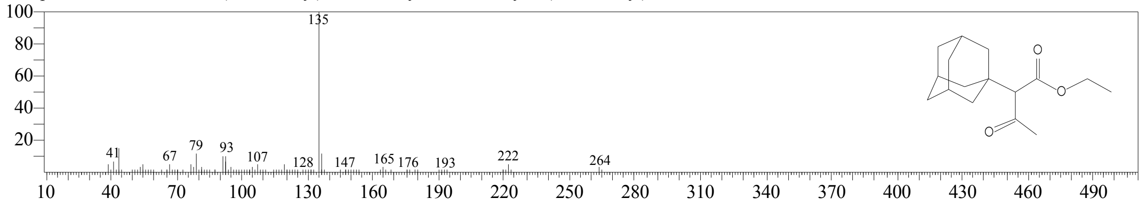

Hit#:5 Entry:77071 Library:NIST14.lib
SI:65 Formula:C14H26OSi CAS:79671-68-4 MolWeight:238 RetIndex:1238
CompName:1-Adamantanemethanol, TMS derivative $$ 1-Trimethylsilyloxymethyladamantane $$ (1-Adamantylmethoxy)(trimethyl)silane # $$

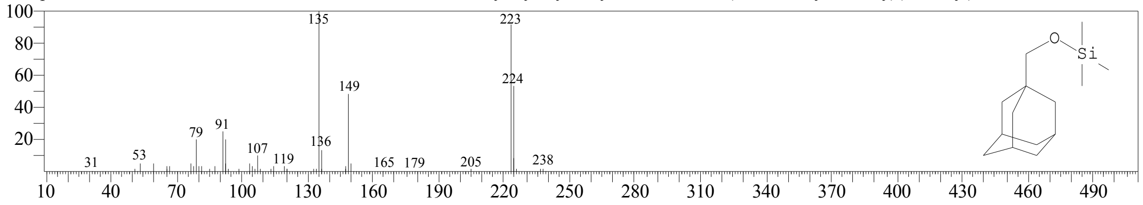

<< Target >>
Line#:16 R.Time:14.370(Scan#:2962) MassPeaks:278
RawMode:Averaged 14.367-14.373(2961-2963) BasePeak:57.10(3977)
BG Mode:Calc. from Peak Group 1 - Event 1 Scan

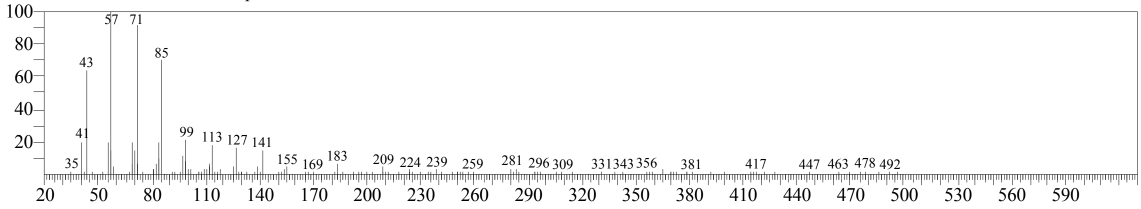

Hit#:1 Entry:237196 Library:NIST14.lib
SI:88 Formula:C30H61I CAS:0-00-0 MolWeight:548 RetIndex:0
CompName:Triacontane, 1-iodo-

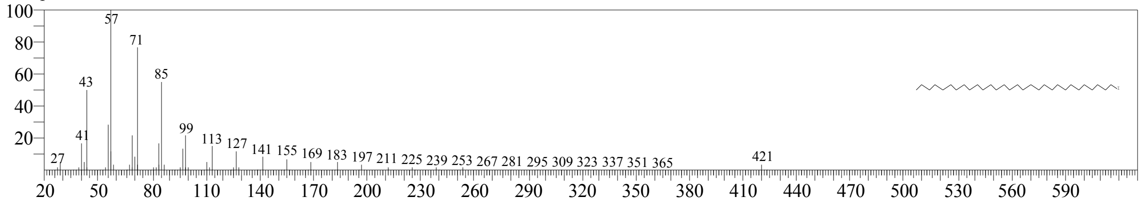

Hit#:2 Entry:239932 Library:NIST14.lib
SI:88 Formula:C44H90 CAS:7098-22-8 MolWeight:618 RetIndex:4395
CompName:Tetratetracontane $$ n-Tetratetracontane $$

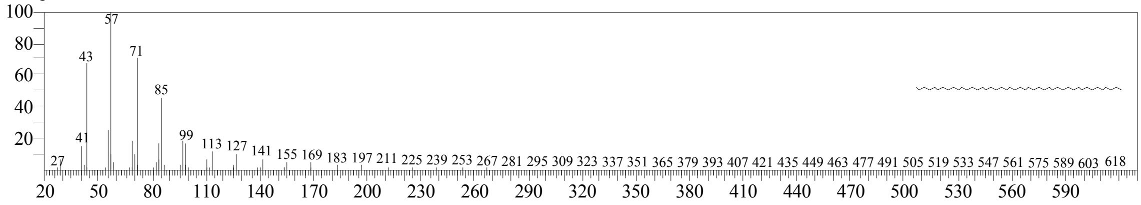

Hit#:3 Entry:209770 Library:NIST14.lib
SI:88 Formula:C29H60 CAS:1560-98-1 MolWeight:408 RetIndex:0
CompName:Octacosane, 2-methyl- $$ 2-Methyloctacosane $$

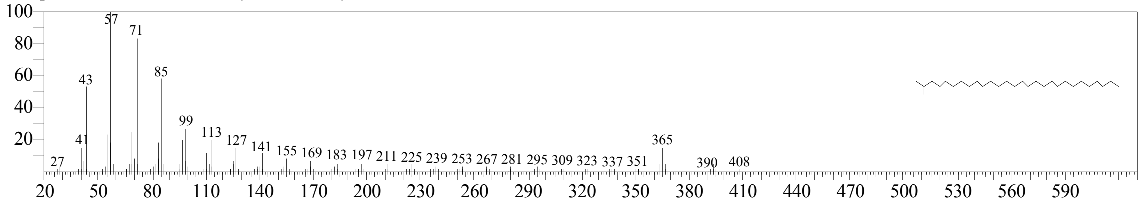

Hit#:4 Entry:238607 Library:NIST14.lib
SI:88 Formula:C32H65I CAS:0-00-0 MolWeight:576 RetIndex:0
CompName:Dotriacontane, 1-iodo-

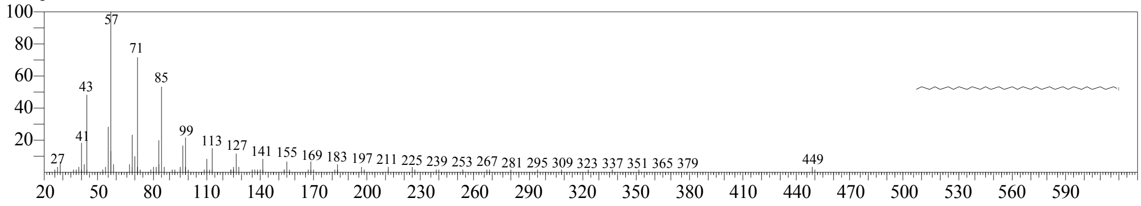

Hit#:5 Entry:209768 Library:NIST14.lib
SI:88 Formula:C29H60 CAS:0-00-0 MolWeight:408 RetIndex:2840
CompName:2-methyloctacosane

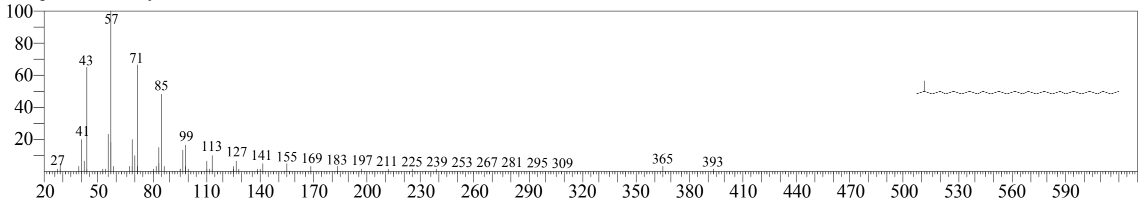

<< Target >>
Line#:17 R.Time:16.117(Scan#:3486) MassPeaks:309
RawMode:Averaged 16.113-16.120(3485-3487) BasePeak:57.05(5146)
BG Mode:Calc. from Peak Group 1 - Event 1 Scan

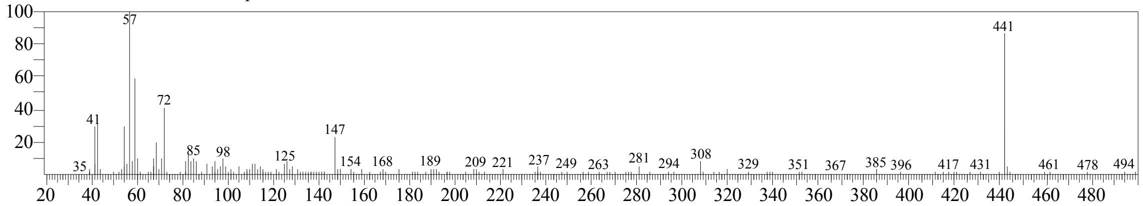

Hit#:1 Entry:196680 Library:NIST14.lib
SI:70 Formula:C24H46O3 CAS:0-00-0 MolWeight:382 RetIndex:0
CompName:Carbonic acid, eicosyl prop-1-en-2-yl ester

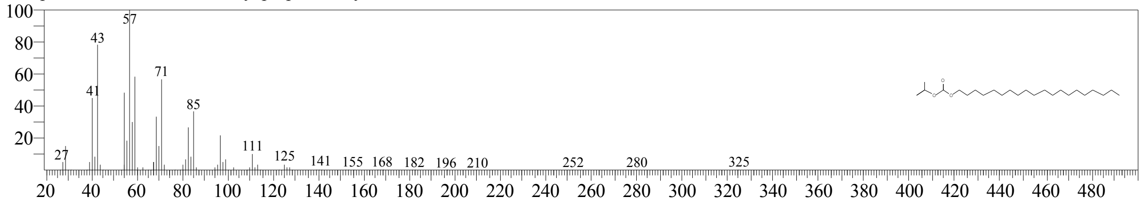

Hit#:2 Entry:177987 Library:NIST14.lib
SI:70 Formula:C22H42O3 CAS:0-00-0 MolWeight:354 RetIndex:0
CompName:Carbonic acid, octadecyl prop-1-en-2-yl ester

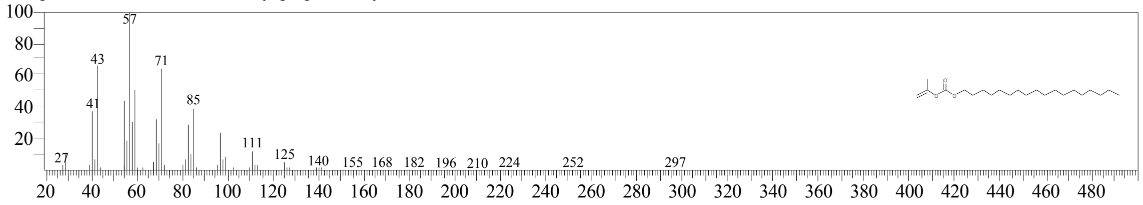

Hit#:3 Entry:166927 Library:NIST14.lib
SI:70 Formula:C21H40O3 CAS:0-00-0 MolWeight:340 RetIndex:0
CompName:Carbonic acid, heptadecyl prop-1-en-2-yl ester

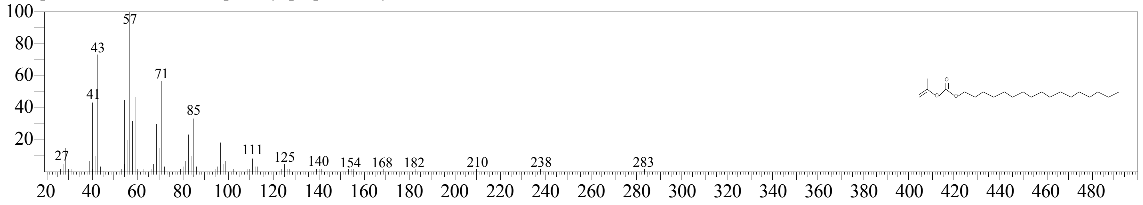

Hit#:4 Entry:154587 Library:NIST14.lib
SI:70 Formula:C20H38O3 CAS:0-00-0 MolWeight:326 RetIndex:0
CompName:Carbonic acid, hexadecyl prop-1-en-2-yl ester

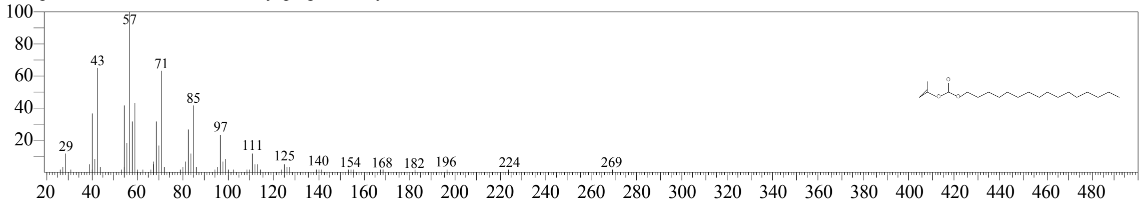

Hit#:5 Entry:142194 Library:NIST14.lib
SI:69 Formula:C19H36O3 CAS:0-00-0 MolWeight:312 RetIndex:0
CompName:Carbonic acid, pentadecyl prop-1-en-2-yl ester

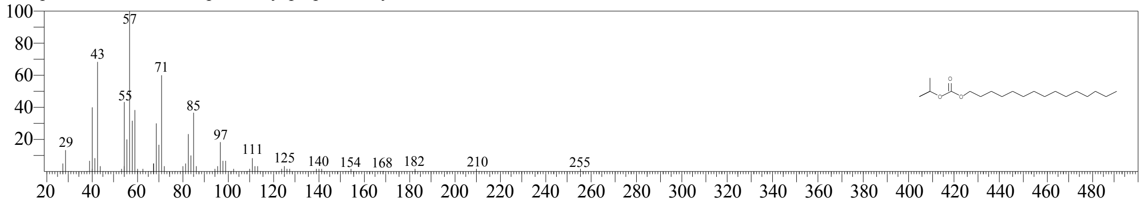

<< Target >>
Line#:18 R.Time:5.557(Scan#:318) MassPeaks:270
RawMode:Averaged 5.553-5.560(317-319) BasePeak:61.00(2649)
BG Mode:Calc. from Peak Group 1 - Event 1 Scan

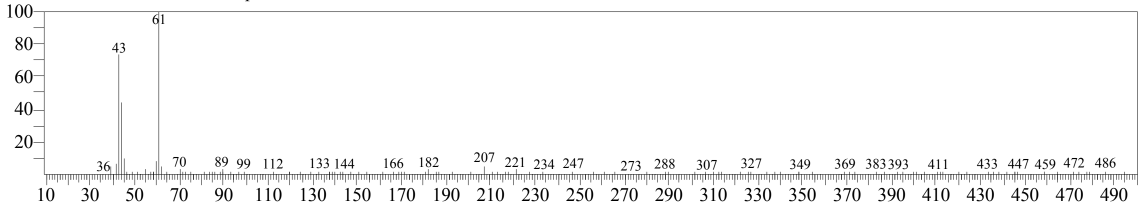

Hit#:1 Entry:1200 Library:NIST14.lib
SI:85 Formula:C3H8O3 CAS:56-81-5 MolWeight:92 RetIndex:967
CompName:Glycerin $$ 1,2,3-Propanetriol $$ Glycerol $$ Glycerine $$ Glyceritol $$ Glycyl alcohol $$ Glyrol $$ Glysanin $$ Osmoglyn $$ Propanetriol $

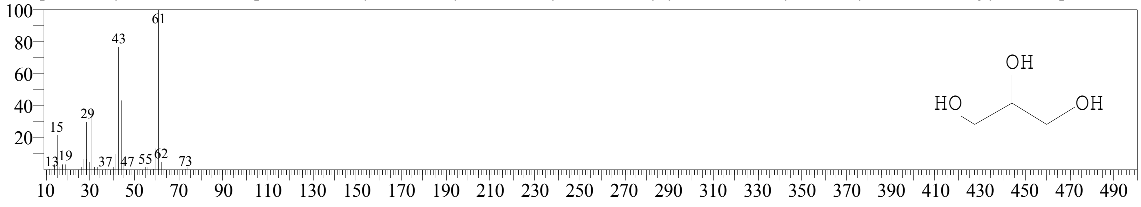

Hit#:2 Entry:1126 Library:NIST14.lib
SI:78 Formula:C3H6O3 CAS:56-82-6 MolWeight:90 RetIndex:913
CompName:Glyceraldehyde $$ dl-Glyceraldehyde $$ Propanal, 2,3-dihydroxy-, (.+/-.)- $$ Glyceraldehyde, dl- $$ Glyceraldehyde, (.+/-.)- $$ DL-Glyceric al

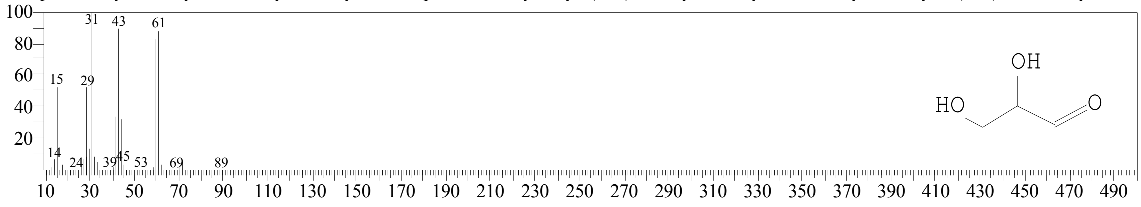

Hit#:3 Entry:23972 Library:NIST14.lib
SI:74 Formula:C6H14O5 CAS:627-82-7 MolWeight:166 RetIndex:1504
CompName:Diglycerol $$ 1,2-Propanediol, 3,3'-oxybis- $$ .alpha.,.alpha.'-Diglycerol $$ Diglycerine $$ 1,2-Propanediol, 3,3'-oxydi- $$ 3,3'-Oxydi-1,2-prop

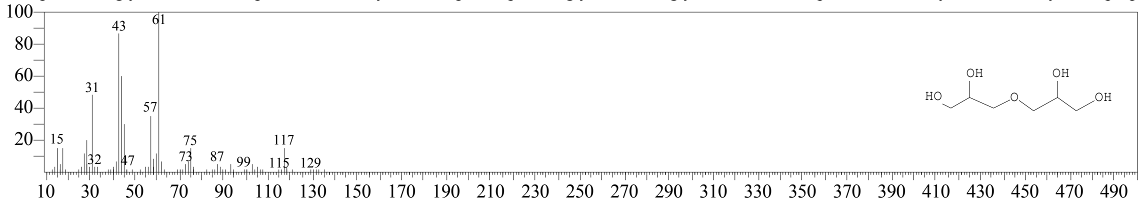

Hit#:4 Entry:5333 Library:NIST14.lib
SI:74 Formula:C5H12O3 CAS:1874-62-0 MolWeight:120 RetIndex:999
CompName:3-Ethoxy-1,2-propanediol $$ 1,2-Propanediol, 3-ethoxy- $$ Glycerol .alpha.-ethyl ether $$ Glycerol .alpha.-monoethyl ether $$ Glycerol 1-ethy

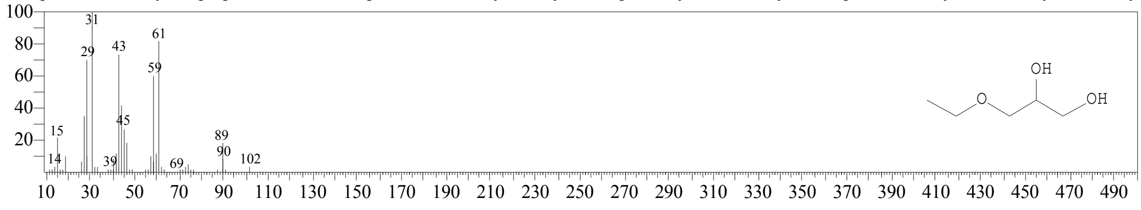

Hit#:5 Entry:5579 Library:NIST14.lib
SI:74 Formula:C4H10O4 CAS:149-32-6 MolWeight:122 RetIndex:1229
CompName:Erythritol $$ 2(R),3(S)-1,2,3,4-Butanetetrol $$ meso-Erythritol $$ 1,2,3,4-Tetrahydroxybutane, (R*,S*)- $$ 1,2,3,4-Butanetetrol, (R*,S*)- $$ E

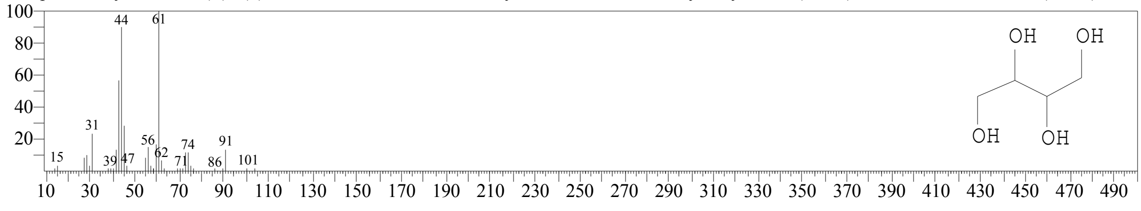

<< Target >>
Line#:19 R.Time:4.700(Scan#:61) MassPeaks:243
RawMode:Averaged 4.697-4.703(60-62) BasePeak:43.00(4302)
BG Mode:Calc. from Peak Group 1 - Event 1 Scan

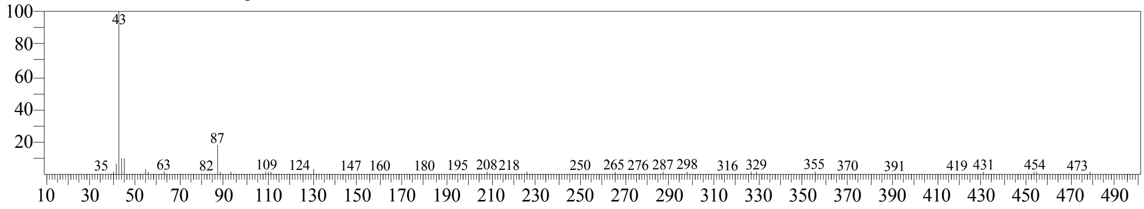

Hit#:1 Entry:7964 Library:NIST14.lib
SI:83 Formula:C6H10O3 CAS:4906-24-5 MolWeight:130 RetIndex:857
CompName:CH3C(O)OCH(CH3)C(O)CH3 $$ 2-Acetoxy-3-butanone $$

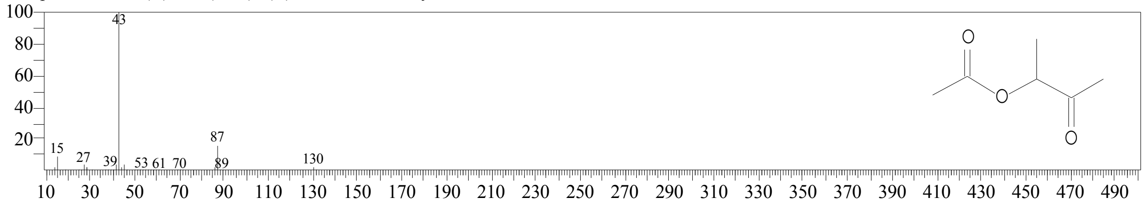

Hit#:2 Entry:13859 Library:NIST14.lib
SI:80 Formula:C6H10O4 CAS:542-10-9 MolWeight:146 RetIndex:888
CompName:1,1-Ethanediol, diacetate $$ Ethylidene acetate $$ Ethylidene diacetate $$ 1,1-Diacetoxyethane $$ 1,1'-Diacetoxy-ethane $$ 1-(Acetyloxy)ethyl

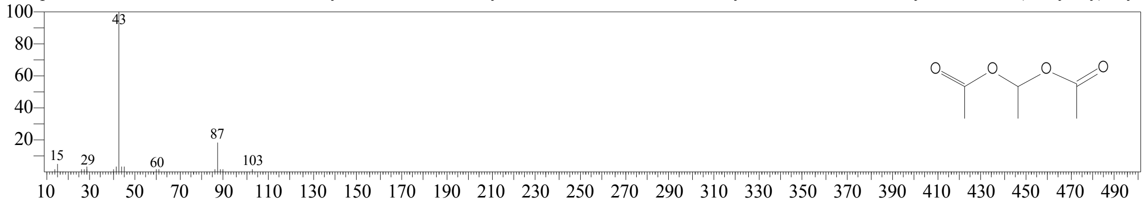

Hit#:3 Entry:972 Library:NIST14.lib
SI:79 Formula:C3H4O3 CAS:127-17-3 MolWeight:88 RetIndex:812
CompName:Propanoic acid, 2-oxo- $$ Pyruvic acid $$ .alpha.-Ketopropionic acid $$ Acetylformic acid $$ BTS $$ Pyroracemic Acid $$ 2-Oxopropanoic ac

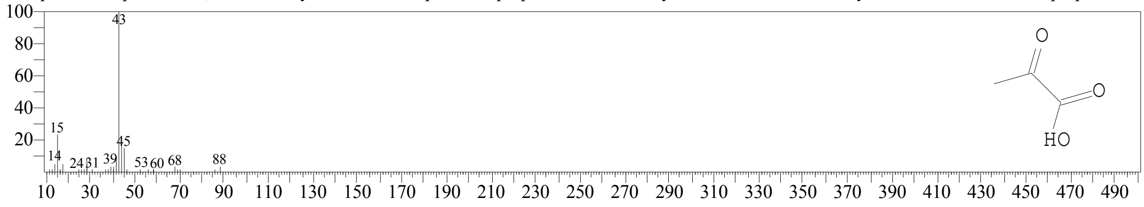

Hit#:4 Entry:39249 Library:NIST14.lib
SI:79 Formula:C8H14O5 CAS:10526-21-3 MolWeight:190 RetIndex:1099
CompName:Ethanol, 1,1'-oxybis-, diacetate $$ Ethanol, 1,1'-oxydi-, diacetate $$ di(1-Acetoxyethyl) ether $$ 1-[1-(Acetyloxy)ethoxy]ethyl acetate # $$

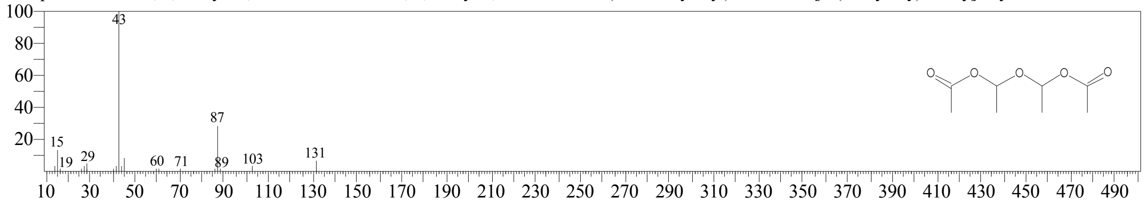

Hit#:5 Entry:29103 Library:NIST14.lib
SI:79 Formula:C8H14O4 CAS:1114-92-7 MolWeight:174 RetIndex:1023
CompName:2,3-Butanediol, diacetate $$ 2-(Acetyloxy)-1-methylpropyl acetate # $$ 2,3-Butanediyl diacetate $$ Butane-2,3-diol, diacetate $$ 2,3-Diacetoxy

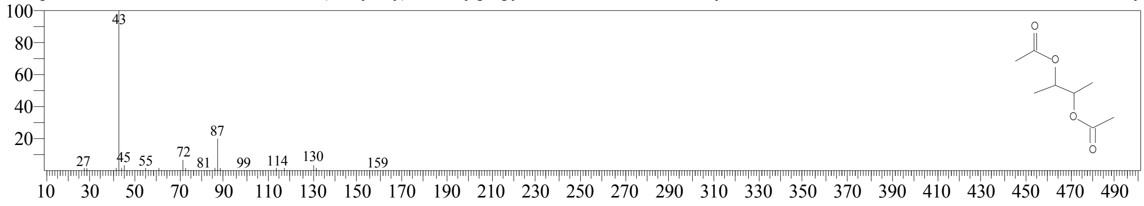

Supplement: Supplementary file 2 [file FSN3-7-2176-s002.pdf]
